# Supplementary material for: Oral resveratrol in adults with knee osteoarthritis: A randomized placebo-controlled trial (ARTHROL)
Source: PLoS Med. 2024 Aug 13;21(8):e1004440. doi: 10.1371/journal.pmed.1004440 (PMC11321588; doi:10.1371/journal.pmed.1004440)
Supplement: S2 Appendix — (DOCX) [file pmed.1004440.s002.docx]

**Appendix 2. Amendments to the original protocol.**

| **No** | **Amendments** | **Protocol version** |
| --- | --- | --- |
| 1 | - Removal and modification of inclusion criteria: inclusion criteria were extended to allow inclusion of patients with Kellgren and Lawrence radiological stage 1 knee osteoarthritis and to allow for earlier management. We also removed the inclusion criterion "No changes in the treatment in the past month" to improve the external validity of the study. - Added clarification of the primary and secondary objectives and endpoints and the selection of study participants. The objectives and endpoints were modified as they were unclear. The participant recruitment strategy was extended to include patients from wards with a diagnosis of "gonarthrosis" in the computerized medical record from 2015 to 2017, with the aim of including more patients and achieving the inclusion targets set. - Modification of the 3 and 6 month follow-up visits in the protocol and information note. It was initially planned that patients would return the questionnaires by mail at the 3 and 6 month visits. Patients will finally be seen in consultation at these follow-up visits, in order to verify compliance with treatment and to retrieve the primary endpoint at 3 months. However, patients will have the possibility to be contacted by phone if they are unable to attend these two visits and to return the questionnaires by mail or email. - Change in the number of patients to be included and addition of information on the information note and consent form. Following the change in the number of patients to be included made at the time of the initial submission of the protocol, the information note had not been rectified. The number of patients included, 164 instead of 160, has therefore been changed in the new version (v2.0) of the information note. A certain amount of information has been added, in particular on the planned collection of drug consumption, on the number of consultations planned per patient, on the investigators' specialty, on the progress of the 3 consultations, on the possible adverse effects of resveratrol and on the possibility of being informed of the study's progress by a newsletter once a month. Finally, the visits at 3 and 6 months became consultations, and the possibility of being contacted by telephone in case of impossibility of going to a consultation was also added | Version 2.0  March 20, 2017 |
| 2 | - Addition of inclusion and non-inclusion criteria: non-inclusion criteria were extended to include patients with systemic steroid therapy and uncontrolled disease that may require systemic steroid therapy, due to possible interactions with the effect of therapy. The possibility of re-screening patients with non-inclusion criteria was also added to improve the external validity of the study. - Modification of some terms considered less appropriate - Description of the statistical analysis made more precise | Version 3.0  July 10, 2017 |
| 3 | - Addition of a non-inclusion criterion: non-inclusion criteria were extended to patients with anticoagulant treatment due to possible interactions with resveratrol via cytochrome. | Version 4.0 du February 18, 2019 |
| 4 | - Extension of the inclusion period by 12 months. - Updating of the serious adverse event notification circuit to the sponsor's vigilance sector. - Addition of anticoagulants as unauthorized treatments following the non-inclusion criterion added by amendment n°3. | Version 5.0  October 14, 2019 |
| 5 | - Extension of the inclusion period by 6 months | Version 6.0  October 13, 2020 |
| 6 | - Extension of the inclusion period by 6 months | Version 7.0  March 23, 2021 |
