# Supplementary material for: Oral resveratrol in adults with knee osteoarthritis: A randomized placebo-controlled trial (ARTHROL)
Source: PLoS Med. 2024 Aug 13;21(8):e1004440. doi: 10.1371/journal.pmed.1004440 (PMC11321588; doi:10.1371/journal.pmed.1004440)
Supplement: S3 Appendix — (DOCX) [file pmed.1004440.s003.docx]

**Appendix 3. Amendments to registration on ClinicalTrials.gov**

| **No** | **Amendments** | **Date** |
| --- | --- | --- |
| 1 | - Modification of one inclusion criterion: "Kellgren and Lawrence X-Ray score 1, 2 or 3" - Suppression of one exclusion criterion: "No changes in the treatment in the past month" | July 20, 2017 |
| 2 | - Addition of two non-inclusion criteria: "Current use of intramuscular, intravenous or oral corticosteroids" and "uncontrolled diseases that may require intramuscular, intravenous or oral corticosteroids" | October 23, 2017 |
| 3 | - Addition of one non-inclusion criterion: "current use of anticoagulants" | June 18, 2019 |
| 4 | - Extension of the study by 12 months | April 2, 2020 |
| 5 | - Extension of the study by 6 months | November 23, 2020 |
| 6 | - Extension of the study by 6 months | June 30, 2021 |
