# Supplementary material for: Oral resveratrol in adults with knee osteoarthritis: A randomized placebo-controlled trial (ARTHROL)
Source: PLoS Med. 2024 Aug 13;21(8):e1004440. doi: 10.1371/journal.pmed.1004440 (PMC11321588; doi:10.1371/journal.pmed.1004440)
Supplement: S4 Appendix — (DOCX) [file pmed.1004440.s004.docx]

**Appendix 4. Demographic and clinical characteristics of participants with missing data on primary efficacy outcome (PEO)**

|  | **Patients with PEO data**  **n=135** | **Patients without PEO data**  **n=7** | **Total**  **n=142** |
| --- | --- | --- | --- |
| Age (years), mean (SD) | 61.5 (9.7) | 60.1 (8.5) | 61.4 (9.6) |
| Women, n (%) | 98 (73) | 3 (43) | 101 (71) |
| Body mass index (kg/m²), mean (SD) | 28.2 (6.2; n=134) | 30.3 (3.9) | 28.3 (6.2; n=141) |
| Higher education, n (%) | 92/135 (68) | 3/7 (43) | 95/142 (67) |
| Employment status, n (%) | | | |
| - Full- or part-time employment | 68 (50) | 4 (57) | 72 (51) |
| - Sick leave | 2 (1) | 1 (14) | 3 (2) |
| - Unable to work | 4 (3) | 0 (0) | 4 (3) |
| - Retired | 61 (45) | 2 (29) | 63 (44) |
| Treatments in the previous 3 months, n (%) | | | |
| - Intra-articular corticoids and/or hyaluronan | 18/133 (14) | 2/7 (29) | 20/140 (14) |
| - Non-opioid oral analgesics | 83/131 (63) | 3/6 (50) | 86/137 (63) |
| - Weak opioid oral analgesics* | 32/124 (26) | 2/7 (29) | 34/131 (26) |
| - Strong opioid oral analgesic* | 1/118 (1) | 1/6 (17) | 2/124 (2) |
| - Oral non-steroidal anti-inflammatory drugs | 58/133 (44) | 3/7 (43) | 61/140 (44) |
| - Symptomatic slow-acting drugs for osteoarthritis | 19/133 (14) | 0/7 (0) | 19/140 (14) |
| - Physiotherapy | 45 (33) | 1 (14) | 46 (32) |
| - Home-based exercises | 57 (42) | 1 (14) | 58 (41) |
| - Foot insoles | 60 (44) | 4 (57) | 64 (45) |
| - Knee brace | 31 (23) | 1 (14) | 32 (23) |
| - Walking aids | 13 (10) | 0 (0) | 13 (9) |
| - Weight management | 50 (37) | 2 (39) | 52 (37) |
| Clinical characteristics, mean (SD) | | | |
| - Knee pain intensity (NRS, 0-100)^§^ | 56.2 (13.7) | 55.7 (9.8) | 56.2 (13.5) |
| - Knee pain duration (years) | 8.5 (8.0; n=134) | 9.5 (11.3) | 8.5 (8.2; n=141) |
| - WOMAC function subscore (0-68)^\|\|^ | 44.3 (16.3) | 44.2 (19.5) | 44.2 (16.4) |
| - Patient global assessment \ (NRS, 0-100)^¶^ | 65.7 (21.3) | 72.9 (18.9) | 66.1 (21.2) |
| X-ray findings in medial or lateral femorotibial or patellofemoral, n (%) | | | |
| - Maximal KL Grade 1 | 23 (17) | 1 (14) | 24 (17) |
| - Maximal KL Grade 2 | 41 (30) | 4 (57) | 45 (32) |
| - Maximal KL Grade 3 | 71 (53) | 2 (29) | 73 (51) |
| KL: Kellgren and Lawrence; NRS: numeric rating scale; SD: standard deviation; WOMAC: Western Ontario and McMaster Universities Osteoarthritis Index  *Weak opioids include codeine, dihydrocodeine, and tramadol. Strong opioids include morphine, diamorphine, fentanyl, buprenorphine, oxymorphone, oxycodone, and hydromorphone  §Higher scores indicate greater pain  ^\|\|^Higher scores indicate more limitations  ^¶^Higher scores indicate better health  n=135 and n=7 per group, respectively (unless indicated otherwise) | | | |
