# Supplementary material for: Oral resveratrol in adults with knee osteoarthritis: A randomized placebo-controlled trial (ARTHROL)
Source: PLoS Med. 2024 Aug 13;21(8):e1004440. doi: 10.1371/journal.pmed.1004440 (PMC11321588; doi:10.1371/journal.pmed.1004440)
Supplement: S5 Appendix — (DOCX) [file pmed.1004440.s005.docx]

**Appendix 5. Sensitivity analysis on primary outcome at 3 months**

| **Change in knee pain (NRS, 0-100), mean (95% CI)** | **Resveratrol**  **n=71** | **Placebo**  **n=71** | **Absolute difference (resveratrol**  **minus placebo) (95% CI)** | **p-value** |
| --- | --- | --- | --- | --- |
| - cLDA (3 and 6 months)* | -15.7 (-21.1 to -10.3) | -15.2 (-20.5 to -9.8) | -0.6 (-8.0 to 6.9) | 0.88 |
| - cLDA (3 months only) | -15.7 (-21.1 to -10.3) | -15.1 (-20.4 to -9.7) | -0.6 (-8.0 to 6.8) | 0.86 |
| - ANCOVA | -12.9 (-18.7 to -7.2) | -12.1 (-17.9 to -6.3) | -0.8 (-8.3 to 6.6) | 0.83 |
| - ANCOVA + worst case scenario | -9.9 (-16.2 to -3.6) | -14.4 (-20.7 to -8.0) | 4.5 (-3.6 to 12.5) | 0.27 |
| - ANCOVA + best case scenario | -15.1 (-21.2 to -8.9) | -9.5 (-15.8 to -3.3) | -5.5 (-13.4 to 2.4) | 0.17 |

ANCOVA: analysis of covariance; CI: confidence interval; cLDA: constrained longitudinal analysis; NRS: numeric rating scale

Worst-case scenario assumes missing NRS knee pain values at 3 months in Resveratrol group had the worst possible value (=100) and those in control group had the best possible value (=0)

Best-case scenario assumes missing knee pain values at 3 months in Resveratrol group had the best possible value (=0) and those in control group had the worst possible value (=100)

*primary analysis
