# Supplementary material for: Oral resveratrol in adults with knee osteoarthritis: A randomized placebo-controlled trial (ARTHROL)
Source: PLoS Med. 2024 Aug 13;21(8):e1004440. doi: 10.1371/journal.pmed.1004440 (PMC11321588; doi:10.1371/journal.pmed.1004440)
Supplement: S6 Appendix — (DOCX) [file pmed.1004440.s006.docx]

**Appendix 6. Co-interventions at 3 and 6 months.**

|  | **Resveratrol**  **n=71** | **Placebo**  **n=71** | **Total**  **n=142** |
| --- | --- | --- | --- |
| **At 3-month follow-up** | | | |
| - Intra-articular corticoids and/or hyaluronan | 5/67 (8) | 6/67 (9) | 11/134 (8) |
| - Non-opioid oral analgesics | 38/67 (57) | 39/64 (61) | 77/131 (59) |
| - Weak opioid oral analgesics* | 12 /62(19) | 14/64 (22) | 26/126 (21) |
| - Strong opioid oral analgesic* | 1/62 (2) | 1/60 (2) | 2/122 (2) |
| - Oral non-steroidal anti-inflammatory drugs | 18/66 (27) | 24/67 (36) | 42/133 (32) |
| - Symptomatic slow-acting drugs for osteoarthritis | 4/66 (6) | 9/67 (13) | 13/133 (10) |
| - Physiotherapy | 13/64 (20) | 14/60 (23) | 27/124 (22) |
| - Home-based exercises | 31/64 (48) | 24/60 (40) | 55/124 (44) |
| - Foot insoles | 26/64 (41) | 24/60 (40) | 50/124 (40) |
| - Knee brace | 14/64 (22) | 7/60 (12) | 21/124 (17) |
| - Walking aids | 10/63 (16) | 9/60 (15) | 19/123 (15) |
| - Weight management | 18/64 (28) | 21/60 (35) | 39/124 (32) |
| **At 6-month follow-up** | | | |
| - Intra-articular corticoids and/or hyaluronan | 7/60 (12) | 5/65 (8) | 12/125 (10) |
| - Non-opioid oral analgesics | 30/59 (51) | 33/63 (52) | 63/122 (52) |
| - Weak opioid oral analgesics | 9/60 (15) | 17/62 (27) | 26/122 (21) |
| - Strong opioid oral analgesic | 1/60 (2) | 1/60 (2) | 2/120 (2) |
| - Oral non-steroidal anti-inflammatory drugs | 15/60 (25) | 20/65 (31) | 25/125 (28) |
| - Symptomatic slow-acting drugs for osteoarthritis | 4/60 (7) | 9/64 (14) | 13/124 (10) |
| - Physiotherapy | 11/58 (19) | 14/63 (22) | 25/121 (21) |
| - Home-based exercises | 29/58 (50) | 24/63 (38) | 53/121 (44) |
| - Foot insoles | 19/58 (33) | 25/63 (40) | 44/121 (36) |
| - Knee brace | 13/58 (22) | 6/63 (10) | 19/121 (16) |
| - Walking aids | 9/58 (16) | 12/63 (19) | 21/121 (17) |
| - Weight management | 19/58 (33) | 17/63 (27) | 36/121 (30) |
| *Weak opioids include codeine, dihydrocodeine, and tramadol. Strong opioids include morphine, diamorphine, fentanyl, buprenorphine, oxymorphone, oxycodone, and hydromorphone  Data are n (%). | | | |
