# Supplementary material for: Oral resveratrol in adults with knee osteoarthritis: A randomized placebo-controlled trial (ARTHROL)
Source: PLoS Med. 2024 Aug 13;21(8):e1004440. doi: 10.1371/journal.pmed.1004440 (PMC11321588; doi:10.1371/journal.pmed.1004440)
Supplement: S1 Method — (PDF) [file pmed.1004440.s007.pdf]

EVOLUTION OF PAIN AT THREE MONTHS BY ORAL RESVERATROL IN PRIMARY KNEE OSTEOARTHRITIS : A MULTICENTER, DOUBLE-BLIND, RANDOMIZED, PLACEBO-CONTROLLED TRIAL

ACRONYM: ARTHROL

Réf projet: **P150938** / ANSM: N° **ID RCB : 2016-A00753-48**

Version N°1.1 of 14/12/2016

Coordinating Investigator / Scientific Director

Prof. François RANNOU, M.D., Ph.D.
Service de Rééducation et de Réadaptation de l'Appareil Locomoteur et des Pathologies du Rachis
Hôpitaux Universitaires Paris Centre - Groupe Hospitalier Cochin
Assistance Publique – Hôpitaux de Paris
27, Rue du Faubourg Saint-Jacques, 75014 Paris, FRANCE
Tel +33 1 58 41 25 35
Fax +33 1 58 41 25 45
francois.rannou@aphp.fr

Dr. Christelle NGUYEN, M.D., Ph.D.
Service de Rééducation et de Réadaptation de l'Appareil Locomoteur et des Pathologies du Rachis
Hôpitaux Universitaires Paris Centre - Groupe Hospitalier Cochin
Assistance Publique – Hôpitaux de Paris
27, Rue du Faubourg Saint-Jacques, 75014 Paris, FRANCE
Tel +33 1 58 41 29 45
Fax +33 1 58 41 28 35
christelle.nguyen2@aphp.fr

Methodologist

Prof. Isabelle Boutron
Centre d'Épidémiologie Clinique
Centre de Recherche Épidémiologie et Statistique Sorbonne Paris Cité (CRESS-UMR1153)

INSERM / Université Paris Descartes
Hôpital Hôtel-Dieu, 1 Place du Parvis Notre-Dame, 75004 Paris, FRANCE
Tel :+33 1 42 34 78 33
isabelle.boutron@htd.aphp.fr

Sponsor

Assistance Publique - Hôpitaux de Paris (AP-HP)
By delegation: Clinical Research and Development Department (DRCD)
Hôpital Saint-Louis
1, Avenue Claude Vellefaux, 75010 Paris, France
Chef de Projet : Sylvie Prieur
Tel + 33 1 44 84 17 92
Fax +33 1 44 84 17 01
sylvie.prieur@aphp.fr

Entity responsible for monitoring research

Unité de Recherche Clinique (URC) Paris Descartes Cochin
GH Cochin – Hôpital Tarnier
89 rue d'Assas
75006 PARIS
Chef de Projet: Alexandra Bruneau
Tel +33 1 58 41 12 13
Fax + 33 1 58 41 11 83
alexandra.bruneau@aphp.fr

Entity responsible for investigational products circuit :

Département Essais cliniques de l'AGEPS
Pharmacist: Dr Blandine Lehmann
Chef de projets : Chrystelle Frappesauce
Tel: +33 1 46 69 14 02

CONTENTS

1 SUMMARY 5

2 SCIENTIFIC JUSTIFICATION FOR THE RESEARCH 8

2.1 HYPOTHESIS FOR THE RESEARCH 8
2.2 DESCRIPTION OF KNOWLEDGE RELATING TO THE HYPOTHESIS FOR THE RESEARCH 9
2.3 JUSTIFICATION OF THE RESEARCH 9
2.4 PRIMARY OBJECTIVE 11
2.5 SECONDARY OBJECTIVES 11

3 PLAN FOR THE RESEARCH 12

3.1 CONCISE DESCRIPTION OF THE PRIMARY AND SECONDARY ASSESSMENT CRITERIA 12
3.2 DESCRIPTION OF RESEARCH METHODOLOGY 13

4 PROCEDURE FOR THE RESEARCH 14

4.1 SELECTION 15
4.2 INCLUSION AND RANDOMIZATION VISIT 15
4.3 FOLLOW-UP SELF-ADMINISTERED QUESTIONNAIRES 16
4.4 END OF RESEARCH 16
4.5 EXPECTED LENGTH OF PARTICIPATION AND DESCRIPTION OF THE CHRONOLOGY AND DURATION OF THE RESEARCH. 17
4.6 TABLE OR DIAGRAM SUMMARISING THE CHRONOLOGY OF THE RESEARCH 17
4.7 DISTINCTION BETWEEN CARE AND RESEARCH 17
4.8 TERMINATION RULES 18

5 ELIGIBILITY CRITERIA 19

5.1 INCLUSION CRITERIA 19
5.2 EXCLUSION CRITERIA 19
5.3 RECRUITMENT METHODS 20

6 EXPERIMENTAL PRODUCTS ADMINISTERED TO RESEARCH PARTICIPANTS 20

6.1 DESCRIPTION OF THE EXPERIMENTAL PRODUCTS 20
6.2 AUTHORISED AND PROHIBITED TREATMENTS (MEDICINAL, NON MEDICINAL, SURGICAL), INCLUDING RESCUE MEDICATIONS 21
6.3 METHODS FOR MONITORING COMPLIANCE WITH THE EXPERIMENTAL PRODUCTS 21

7 ASSESSMENT OF EFFICACY 21

8 STATISTICAL ASPECTS 22

8.1 CALCULATION OF SAMPLE SIZE 22
8.2 DESCRIPTION OF STATISTICAL METHODS TO BE USED 22
8.3 STATISTICAL ANALYSIS PLAN 22

9 SAFETY ASSESSMENT - RISKS AND RESTRICTIONS ADDED BY THE RESEARCH . 23

9.1 DEFINITIONS 23
9.2 THE INVESTIGATOR'S ROLES 23
9.3 SPECIFIC FEATURES OF THE PROTOCOL 24
9.4 PROCEDURES AND DEADLINES FOR NOTIFYING THE SPONSOR 26
9.5 PERIOD FOR NOTIFYING THE SPONSOR 26
9.6 THE SPONSOR'S ROLES 26
9.7 DATA SAFETY MONITORING BOARD (DSMB) 28

10 DATA MANAGEMENT 28

10.1 DATA COLLECTION METHODS 28
10.2 RIGHT TO ACCESS SOURCE DATA AND DOCUMENTS 29
10.3 DATA PROCESSING AND STORAGE OF DOCUMENTS AND DATA 30
10.4 OWNERSHIP OF THE DATA 30

11 QUALITY CONTROL AND ASSURANCE 30
11.1 General organisation 31
11.2 Level of centre monitoring 31
11.3 Quality control 31
11.4 Case Report Form (CRF) 32
11.5 Management of non-compliances 32
11.6 Audits/inspections 32
11.7 Primary investigator's commitment to assume responsibility 33
12 ETHICAL AND LEGAL CONSIDERATIONS 33
12.1 Methods for obtaining information and consent from research participants 33
12.2 Subject prohibited from participating in another research or an exclusion period anticipated after the research, if applicable 34
12.3 Compensation for subjects 34
12.4 Legal obligations 34
12.5 Modifications to the research 35
12.6 Final research report 35
13 FUNDING AND INSURANCE 35
13.1 Funding source 35
13.2 Insurance 35
14 PUBLICATION RULES 36
14.1 Mention of the AP-HP manager (DRCD) in the acknowledgements of the text 36
14.2 Mention of the financier in the acknowledgements of the text 36
15 BIBLIOGRAPHY 36
16 LIST OF ADDENDA 40
16.1 List of Investigators 40
16.2 Annexes 41

1 SUMMARY

Title	Evolution of pain at three months by oral resveratrol in primary knee osteoarthritis: a multicenter, double-blind, randomized, placebo-controlled trial
Acronym	ARTHROL
Coordinating Investigator<br><br><br><br><br><br><br><br><br><br>Scientific Director	Prof. François RANNOU, M.D., Ph.D.<br>Service de Rééducation et de Réadaptation de l'Appareil Locomoteur et des Pathologies du Rachis<br>Hôpitaux Universitaires Paris Centre - Groupe Hospitalier Cochin<br>Assistance Publique – Hôpitaux de Paris<br>27, Rue du Faubourg Saint-Jacques, 75014 Paris, FRANCE<br>Tel +33 1 58 41 25 35<br>Fax +33 1 58 41 25 45<br>francois.rannou@aphp.fr<br><br>Dr. Christelle NGUYEN, M.D., Ph.D.<br>Service de Rééducation et de Réadaptation de l'Appareil Locomoteur et des Pathologies du Rachis<br>Hôpitaux Universitaires Paris Centre, Groupe Hospitalier Cochin<br>Assistance Publique – Hôpitaux de Paris<br>27, Rue du Faubourg Saint-Jacques, 75014 Paris, FRANCE<br>Tel +33 1 58 41 29 45<br>Fax +33 1 58 41 28 35<br>christelle.nguyen2@aphp.fr
Sponsor	Assistance Publique - Hôpitaux de Paris
Scientific justification	OA is the first cause of handicap in individuals over 40 years-old in France. OA physiopathology is driven by local joint inflammation responsible for pain and joint destruction. Experimental studies have shown that resveratrol could modulate pain and inflammation. We hypothesize that resveratrol, in a new formulation developed by the coordinating investigator and his colleagues (INSERM U1124), improving its bioavailability, will decrease pain in patients presenting with primary knee OA.
Primary objective and assessment criterion	Change in mean knee pain in the past 48 hours on an 11-point numeric rating scale (NRS, 0 no pain - 100 maximal pain) at 3 months
Secondary objectives and assessment criteria	• Change in mean knee pain in the past 48 hours on NRS at 6 months<br>• Western Ontario and McMaster Universities Arthritis Index (WOMAC) function subscore at 3 and 6 months<br>• Patient's global assessment of improvement at 3 and 6 months on NRS<br>• Osteoarthritis Research Society International (OARSI) - Outcome Measures in Rheumatology (OMERACT) response at 3 and 6 months<br>• Self-reported analgesics and non-steroidal anti-inflammatory drugs (NSAIDs) consumption and intra-articular corticoid or hyaluronic acid injections since last contact at 3 and 6 months
Experimental design	Multicenter, double-blind, randomized, placebo-controlled trial

Population involved	Patients presenting with symptomatic primary knee OA, fulfilling 1986 American College of Rheumatology (ACR) classification criteria
Inclusion criteria	• Age ≥ 40 years-old<br>• Knee OA fulfilling 1986 ACR criteria<br>• Pain on NRS ≥ 40/100<br>• Symptom duration ≥ 1 month<br>• 4 > Kellgren and Lawrence X-Ray score ≥ 2<br>• No changes in the treatment in the past month<br>• Written consent obtained<br>• Health insurance cover
Non-inclusion criteria	• History of symptomatic crystal or inflammatory arthritis<br>• Knee surgery ≤ 1 year<br>• Knee trauma ≤ 2 months<br>• Knee intra-articular injections ≤ 2 months<br>• Neurologic disorders involving the lower limbs<br>• Inability to speak, write or read French language<br>• Participation to another biomedical research<br>• Contraindication to resveratrol or hypersensitivity to any of its constituents
Investigational product	Resveratrol is a dietary supplement, not a drug. It will be administered orally 2 capsules twice a day for one week then one capsule twice a day for a total duration of 6 months. Resveratrol will be supplied by the industrial partner. The capsules of resveratrol have already been distributed on the French market for several years and the capsules used in this study will be exactly the same as those already available on the French market.
Control group	Placebo of resveratrol will be supplied by the industrial partner. It will present with similar conditioning and taste, and will be administered orally 2 capsules twice a day for one week then one capsule twice a day for a total duration of 6 months. The placebo use in this study will be exactly the same as the one used in a previous PHRC already accepted in neurology (Assistance Publique-Hôpitaux de Paris).
Other procedures added by the research	Not applicable
Risks added by the research	Risk A
Practical procedure	• Day -50: screening of eligible patients<br>• Day 0: inclusion visit, informed consent, inclusion and randomization<br>• Month 3: auto-questionnaire follow-up<br>• Month 6: end of the research
Number of subjects chosen	164
Number of centres	3 tertiary care centers located in France<br>• Cochin Hospital, Paris<br>• Saint-Antoine Hospital, Paris<br>• Clermont-Ferrand Hospital
Research period	• Duration of participation for each patient: 6 months

	• Duration of recruitment: 24 months<br>• Total duration: 30 months
Number of inclusions expected per centre and per month	2.3 patients per centre and per month
Statistical analysis	Analysis will be performed according to the intention-to-treat principle by which each participant will be analyzed in his randomization arm, following a prespecified statistical analytic plan
Funding source	Assistance Publique-Hôpitaux de Paris - Programme Hospitalier de Recherche Clinique en 2015
Data Safety Monitoring Board anticipated	No

2 SCIENTIFIC JUSTIFICATION FOR THE RESEARCH

2.1 Hypothesis for the research

In the Global Burden of Disease Study 2013 designed to assess disability-adjusted life-years (DALYs), which is an indicator of health loss, musculoskeletal disorders were identified among the 5 main contributors to DALYs [1]. Knee osteoarthritis (OA) is one of the most disabling joint disorder in Western countries [2]. OA is the first cause of disability in patients over 40 years-old in France [3]. OA physiopathology is driven by local joint inflammation responsible for pain and joint destruction. There is no efficient structural treatment for OA, but only symptomatic treatments, mainly acetaminophen and NSAIDs, designed to alleviate painful symptoms. Unfortunately, acetaminophen is not very effective with an effect size of 0.10, and recent data highlighted its potential cardiovascular adverse effects [4, 5]. NSAIDs are efficient on pain and inflammation, but their serious cardiovascular and digestive side effects do not support a prescription for long duration. Therefore, an optimized treatment in OA should be efficient on both pain and inflammation, as well as innocuous. Resveratrol is an interesting molecule because of its anti-inflammatory properties [6]. Resveratrol is currently used as a dietary supplement. No serious toxicity has been described in humans so far.

A French PHRC is currently ongoing to address resveratrol positive effects on metabolic syndrome and inflammation: AOR12; METARES study, Prof. Jean-Louis Beaudeux, Pitié-Salpêtrière Hospital, Pierre & Marie Curie University. Resveratrol has also been evaluated in aging, cancer, neurodegenerative diseases, menopausal conditions, and cardiovascular and liver diseases [6]. However, the doses used in these trials were highly variable, and were not adjusted regarding the low bioavailability of current oral formulations.

Drs JF Savouret (INSERM U1124) and Éric Serée (scientific advisor for our project) have recently designed and patented a new formulation of resveratrol, in collaboration with our industrial partner (Laurent Pechère, Yvery Laboratory, Marseille, FRANCE). This new formulation has allowed for the first time a significant increase of resveratrol bioavailability by oral administration in humans, in contrast with former dry powder formulations [7]. The plasmatic peak (30 min. after ingestion) was 10-fold increased, and blood concentration remained at significant levels during several hours. This new resveratrol formulation may allow for a trial with low resveratrol doses (40 mg/day), yet yielding a significant blood level and half-life of resveratrol. In other word, with this new formulation of resveratrol, a putative effect becomes possible via oral administration. To our knowledge, this formulation is unique and no other formulation is able to reach such a high bioavailability.

We hypothesized that the use of resveratrol in this new formulation would significantly decrease mean OA knee pain in the past 48 hours at 3 months, with no adverse effects.

2.2 Description of knowledge relating to the hypothesis for the research

Mediterranean societies have developed the 'Mediterranean diet' under the influence of Greek medicine. This diet consists in a moderate intake of animal meat and fat in combination with high consumption of vegetables, fruits and olive oil. Natural antioxidants, fibers, B vitamins and unsaturated fatty acids are also present, with red wine being the main alcoholic beverage, consumed with meals on a daily basis [8]. The beneficial effect of this diet upon coronary heart disease prevention has been a matter of debate, recently refueled by the controversy over the 'French Paradox'. This concept arose from studies showing the low rate of coronary heart disease in wine-drinking French population compared to other Western populations, despite the presence of elevated risk factors including high animal fat intake, low exercise level, and heavy smoking [9]. This controversy started on epidemiological grounds, further fueled by negative experimental data [10], and eventually lead to the hypothesis that red wine may contain cardioprotective compounds like antioxidant polyphenols.

Red wine contains several polyphenols including phenolic acids, hydroxystilbenes and flavonoids. Trans-resveratrol (3,5,4'-trihydroxystilbene) is the parent compound of a family of hydroxystilbenes existing in cis- and trans- configurations in a variety of spermatophyte plants such as grapevine, peanuts, pine, or Chinese knotweed [10]. Because of its effects on lipids and arachidonic acid metabolisms, and its antioxidant activity, resveratrol has been considered as the major cardioprotective component of red wine. In grapes, resveratrol is produced as an antifungal phytoalexin in response to infection by *Bothrytis Cinerea*. Resveratrol is present in red wine at concentrations ranging from 0.1 to 14.5 mg/L [11]. Several Asian pharmacopoeas describe Chinese knotweed (*Polygonum Cuspidatum*) powder as an anti-inflammatory drug, used in various conditions such as pain, fever, dermatitis, atherosclerosis, hyperlipemia and cancers [12]. **Experimental and clinical data suggest that resveratrol anti-inflammatory and analgesic effects may be of clinical interest as a complementary to conventional treatment in joint diseases. Hypotheses about resveratrol mechanisms of action suggest a modulation of endocrine disruption, apoptosis and oxidative stress.**

2.3 Justification of the research

Even though the research on the effect of dietary polyphenols on human health has developed considerably and controversially in the past decade in such broad fields as cancer, neurodegenerative and cardiovascular diseases, type 2 diabetes mellitus and other metabolic disorders [13], no large clinical data are available to date regarding the efficacy of resveratrol in joint disorders, especially in OA. This lack of trials in joint disorders might be related to controversies associated with the relatively poor results from resveratrol supplementation in other human conditions, particularly with regards to the financial investments involved [14, 15]. However, a larger body of experimental

evidence suggests that resveratrol administered with proper timing and sufficient biodisponibility, might be relevant as a complementary to conventional treatment for joint disorder-related symptoms in humans, in addition to conventional treatments.

***In vitro* findings**

Articular chondrocytes are resistant to resveratrol stimulation at micromolar doses (0.1 to 10 µM), whatever the molecular pathway considered. Several hypotheses can be brought up. Chondrocytes contain high levels of ceruleoplasmin, a copper-containing protein with a strong laccase activity [16]. Laccases are able to disrupt and inactivate resveratrol propylene chain. Chondrocytes also harbour a particular AhR that does not have basal activity and is only able to translocate to the cell *nucleus* upon interleukin (IL)-1β activation (personal data). This apparent insensitivity of chondrocytes to resveratrol *in vitro* due to a lack of AhR basal activity is in contrast with our preliminary clinical observations, showing the potent analgesic activity of resveratrol in patients suffering from a variety of inflammatory joint diseases (personal data). Furthermore, there is growing evidence of resveratrol effects on chondrocytes and synoviocytes.

Resveratrol inhibitory effects on IL-1β and kinases modulation of matrix metalloproteinases (MMP)-1, -3, and -13 expressions in chondrocytes (endochondral cartilage and intervertebral disc) have been reported by several groups [17-21]. In some of these studies, resveratrol doses ranged from 50 to 100 µM. Therefore, an indirect estrogenic effect *via* ER-α activation cannot be excluded [22-25]. Other groups have recently opened a new avenue in cartilage biology research by showing that synoviocytes, more than chondrocytes, might be the real target of resveratrol anti-inflammatory activities. Resveratrol inhibits IL-1β, MMP-3 and phosphorylated Akt expression, either basal or Tumour Necrosis Factor (TNF) α-induced, in a dose-dependent manner, between 6 and 50 µM [26]. Caspase-8 has also been reported to be a target of resveratrol in synoviocytes at high doses (50 µM) [27]. All the authors concluded that resveratrol might have beneficial effects in preventing and treating rheumatoid arthritis (RA). Finally, resveratrol inhibits cell adhesion between monocytes and endothelial cells [28], a mechanism that might be extended to monocyte interactions with chondrocytes. The authors attributed this effect to tyrosine kinase inhibition, the other major effect of resveratrol with AhR activation, although the latter mechanism is also a potent pathway for ICAM-I expression [29].

Pre-clinical findings

Although *in vitro* data strongly support a potent joint protection effect of resveratrol through modulation of inflammation, chondrolysis and angiogenesis, resveratrol health benefits still await in-depth investigation in animal models. Many reports used questionable modes of administration (wine or dry powder form) or erroneous targets, as once pointed out about platelet aggregation [10]. Only few papers have specifically investigated effects of resveratrol in OA animal models.

In a rabbit model of OA, by unilateral anterior cruciate ligament transection, intra-articular injections of resveratrol hampered the progression of cartilage destruction and associated prod-degradative soluble factor production [30, 31]. In Wang's study, intra-articular resveratrol was administered daily for 2 weeks at different dose regimen (50, 20, and 10 µmol/kg). In the groups treated with resveratrol, reduced cartilage lesions, apoptosis rate of chondrocytes and level of nitric oxid in the synovial fluid were observed in a dose-dependent fashion [31]. Consistently, Elmali and colleagues showed a reduction in cartilage destruction scores and loss of matrix proteoglycans in animals injected intra-articularly with 10 µmol/kg resveratrol for 2 weeks compared to DMSO injected animals. Scores of synovial inflammation were comparable between the 2 groups [30]. Most recently, in a mouse model of OA, by destabilization of the medial meniscus, weekly intra-articular injection of resveratrol in the knee was associated with decreased cartilage and subchondral bone changes, along with unchanged type 2 collagen expression, and reduction in iNOS and MMP-13 expressions, activation of SIRT-1 and inhibition of Hypoxia Inducible Factor (HIF)-2α [32]. To our knowledge, no study has reported the effects of oral administration of resveratrol in these models, or the influence of genetic inhibition of AhR in AhR -/-.

In summary, in the field of rheumatic disorders, *in vitro* evidence clearly support anti-inflammatory, anti-catabolic, anti-apoptotic and anti-oxidative properties of resveratrol in various articular cell types including chondrocytes and synoviocytes, along with immunomodulation properties on T and B lymphocytes. Consistently, resveratrol administered intra-articularly has shown joint protective effects in pre-clinical models of OA, mainly mediated by decreased production of pro-inflammatory and pro-degradative soluble factors, as well as modulation of cellular and humoral responses.

In order to take the use of resveratrol to the next step of clinical trials in human joint diseases, we believe that new formulations of resveratrol as developed by our industrial partner, that improve its biodisponibility and safety [7], could be interesting in treating painful symptoms related to OA as a complementary treatment to conventional treatment.

2.4 Primary objective

The primary objective of the study is to assess the impact of **resveratrol** compared to placebo on change in mean knee pain in the past 48 hours at 3 months.

2.5 Secondary objectives

The secondary objectives of the study are to assess the impact of **resveratrol** compared to placebo on:

- Change in mean knee pain in the past 48 hours at 6 months

- WOMAC function subscore at 3 and 6 months
- Patient's global assessment of improvement since last assessment at 3 and 6 months
- OARSI-OMERACT response at 3 and 6 months
- Analgesics and NSAIDs consumption, and intra-articular corticoid or hyaluronic acid injections at 3 and 6 months

3 PLAN FOR THE RESEARCH

3.1 Concise description of the primary and secondary assessment criteria

Primary and secondary assessment criteria core set was selected accordingly with OMERACT recommendations [33] and COMET initiative for phase III clinical trials in knee OA, and includes outcomes for pain, physical function, and patient global assessment of improvement.

Primary assessment criterion

The primary assessment criterion of the study is change from baseline in mean knee pain intensity in the past 48 hours at 3 months. Mean knee pain intensity in the past 48 hours will be defined by the patient, using a self-administered 11-point NRS (0 no pain - 100 maximal pain) (Annex 1).

Secondary assessment criteria

- Change in mean knee pain at 6 months: defined as change from baseline in mean knee pain intensity in the past 48 hours at 6 months, using a self-administered 11-point NRS **(Annex 1)**.
- Mean WOMAC physical function subscore at 3 and 6 months: defined as mean WOMAC physical function subscore at 3 and 6 months [34], using the French version of the questionnaire **(Annex 2).** The WOMAC index is a self-administered, disease specific instrument validated for OA. It consists of 24 items grouped into 3 subscales: pain (5 questions), stiffness (2 questions) and physical function (17 questions) with higher scores indicating greater disease severity [34].
- Patient's global assessment of improvement since last contact at 3 and 6 months, using a self-administered 11-point NRS (0 no improvement - 100 maximal improvement) **(Annex 3)**.
- OARSI-OMERACT response at 3 and 6 months: treatment response will be defined as an improvement in pain or in function ≥ 50% and absolute change ≥ 20, or improvement in at least 2 of the 3 following: 1/ pain ≥ 20% and absolute change ≥ 10, 2/ function assessed by the WOMAC physical function subscore ≥ 20% and absolute change ≥ 10, 3/ patient's global assessment ≥ 20% and absolute change ≥ 10 at 3 and 6 months [35] **(Annex 4).**

- Self-reported analgesics and NSAIDs consumption, and corticoid and hyaluronic intra-articular injections since last contact at 3 and 6 months

3.2 Description of research methodology

Experimental plan. The proposed study is a 2-arm, placebo-controlled, double-blind, multicentre, randomized trial. Patients will be assessed at baseline, 3 and 6 months. They will be randomized in one of two groups:

- Intervention group: 2 capsules of resveratrol each morning and evening for one week, then one capsule morning and evening.
- Control group: placebo in same conditioning, with the same taste, and same way of administration.

Duration of participation for each patient will be 6 months. The study will be planned, executed, analyzed and reported according to CONSORT Statement [38].

Number of centres participating. The proposed study is a national multicenter study. Individuals will be prospectively recruited among in- and outpatients who fulfill the inclusion criteria from 3 French Rheumatology and Rehabilitation Medicine departments located in tertiary care centers with high expertise in OA management.

Identification of the subjects. For this research, participants will be identified as follows: *centre n° (3 numerical positions) – selection order n° of the participant in the research (4 numerical positions) – surname initial - first name initial. This reference is unique and will be used for the entire research period. A randomization number will be assigned during randomization*

Randomization. Patients who meet the inclusion criteria and agreed to participate will be randomly assigned to the resveratrol or placebo group at day 0.

Sequence generation. The randomization sequence will be computer-generated by a statistician of the Centre d'Épidémiologie Clinique. The list will be stratified by centres with variable block sizes.

Implementation. The randomization process will be centralized at the coordinating office (*Unité de Recherche Clinique, Cochin Hospital*), which will have no involvement in the enrollment, follow-up, or assessment of participants. Only the independent statistician of the Centre d'Épidémiologie, the computer programmer at the coordinating office (*Unité de Recherche Clinique, Cochin Hospital*) who will implement the list in the secure electronic case report form (e-CRF), and the YVERY company who will prepare the resveratrol and placebo

capsules according to the randomization list will have access to the randomization list. The YVERY company will label the resveratrol and placebo capsules and send them to each centre for the whole research duration. In each centre, the investigator will blindly deliver the medication to patients enrolled in the study according to their randomization number.

Allocation concealment. The sequence will be concealed by use of a computer interface implemented in the e-CRF (CleanWeb).

Blinding methods and provisions put in place to maintain blindness. Patients, care providers, data collectors, outcome assessors and statisticians will be blinded to the allocated group. The industrial partner will supply *resveratrol and placebo capsules* with strictly identical presentations, treatment administration and clinical monitoring of the experimental products *will be the same in the experimental and control groups.. Resveratrol and placebo capsules* will be prepared according to the randomization list. Labelling will be anonymized by the YVERY company. They will be delivered at once for the whole research duration.

Procedures for breaking the blind, if applicable. The event of having to break the blind is unlikely with the use of a dietary supplement. The blind can be broken only if the investigator deems it necessary for the safe *management of a specific medical condition* of a subject, and whenever possible the medical monitoring methodologist and sponsor should be consulted before breaking the blind. If the blind is broken for any reason during the course of the study, the moment on which the blind was broken and all other relevant information will be documented by the investigative site, and other sponsor designees, as appropriate. The reason for breaking the blind will be indicated and justified in the source documentation and in the eCRF. All subjects who are unblinded while on the study will be followed-up from the moment of unblinding, with the reason for unblinding given. If an *an adverse event (*AE) leads to unblinding, the AE should be given as the reason for unblinding and the AE should also be recorded in the eCRF. Any AEs should be followed until resolution.

Unblinding will be requested for any reason considered essential by the investigating doctor by calling upon:

- the DRCD **in a situation other than an emergency** during the work day and during working hours, addressed to the DRCD's project referent **Tel: +33 1 44 49 59 69**
- the poison centre of Fernand Widal Hospital, in the case of an emergency (see emergency situations requiring unblinding), on weekends, bank holidays, when the DRCD is closed and when unblinding cannot be carried out at the DRCD **Tel: +33 1 40 05 48 48**

4 PROCEDURE FOR THE RESEARCH

4.1 Selection

Participants pre-selection will be performed by the physician among in- and out-patients and by the biomedical research technician on internet or phone among patients who would have replied to announcements made online and using posters displayed in each investigating centres, respectively. No later than 50 days after the pre-selection step, selected participants will be appointed for an inclusion and randomization visit with one of the investigator. Inclusion and exclusion criteria will be checked, the subject will be informed and his consent collected. Specific additional clinical examination, laboratory tests or imaging will not be required for the purpose of the study.

4.2 Inclusion and randomization visit

The inclusion and randomization visit will represent day 0. Inclusion and exclusion criteria will be checked by the investigator or co-investigator, who will be a senior specialist in physical rehabilitation medicine and/or rheumatology, during a consultation dedicated to the biomedical research. Once the patient has agreed to participate and that informed consent is obtained by the investigator, the patient will be enrolled and randomized.

Specific additional clinical examination, laboratory tests or imaging will not be required for the purpose of the study. Information's regarding demographics, medical history, previous and current medications, as well as baseline self-administered auto-questionnaires will be collected:

- Mean knee pain in the past 48 hours on NRS
- WOMAC physical function subscore

Inclusion visit will include :

- Verification of inclusion and non-inclusion criteria
- Patient information and informed consent
- Randomization by eCRF
- Recording of previous and current treatments
- Give to the patient the participating card in a clinical trial **(Annex 6)**
- Give to the patient a pre-filled patient-booklet for compliance
- Dispensation experimental product by the investigator to the patient according the number randomization assigned
- Filling the self-administered questionnaires

**Subjects whose**	**Who informs the subject and**	**When is the subject**	**When is the subject's**

consent is sought	collects their consent?	informed?	consent collected?
Subjects who are participating to the biomedical research	The investigator and co-investigators participating in the study	At the preselection contact and at the inclusion visit	At the inclusion visit

4.3 Follow-up self-administered questionnaires

Follow-up questionnaires will be self-administered by the participants from home and returned by mail to the clinical research technician at 3 months. The following informations will be recorded:

- Mean knee pain in the past 48 hours on NRS
- WOMAC physical function subscore
- Patient's global assessment in improvement since last contact on NRS
- Self-reported analgesics and NSAIDS consumption, and intra-articular corticoid or hyaluronic injections since last contact
- Self-reported adverse events since last contact
- Self-monitored compliance booklet

In case of missing data, the clinical research technician will contact the participant by phone or e-mail to collect the missing data. In order to reduce the amount of missing data and improve compliance to the treatment, reminding newsletters will regularly inform patients of the progression of the study. Compliance will be self-monitored using a pre-filled patient-booklet. No follow-up visit will be required.

4.4 End of research

Follow-up questionnaires will be self-administered by the participants from home and returned by mail to the clinical research technician at 6 months. The following informations will be recorded:

- Mean knee pain in the past 48 hours on NRS
- WOMAC physical function subscore
- Patient's global assessment in improvement since last contact on NRS
- Self-reported analgesics and NSAIDS consumption, and intra-articular corticoid or hyaluronic injections since last contact
- Self-reported adverse events since last contact
- Self-monitored compliance booklet

Patients will continue their usual medical follow-up. No specific changes will be made. Ending a subject's participation will not affect the normal management of the subject's illness in any way. No exclusion period for another biomedical research will be required.

4.5 Expected length of participation and description of the chronology and duration of the research.

Maximum period between selection and inclusion	50 days
Inclusion period	24 months
The included subjects' length of participation, of which:	
• Treatment period:	6 months
• Follow-up period:	6 months
Total research period:	30 months

4.6 Table or diagram summarising the chronology of the research

**Actions**	**Screening**	**Inclusion visit**	**Month 3 ± 10 days**	**Month 6 ± 10 days**
Informed consent		X		
Screening (phone/internet/consultations)	X			
Inclusion/exclusion criteria fulfilment		X		
Randomization		X		
Medical history		X		
Dispensation of experimental products		X		
Self-administered questionnaires<br>- Pain on NRS<br>- WOMAC questionnaire<br>- Global assessment of improvement on NRS		X	X	X
OARSI - OMERACT response			X	X
Compliance			X	X
Co-interventions			X	X
Adverse events			X	X

4.7 Distinction between care and research

Procedures and treatments carried out as part of the research	Procedures and treatments associated with <u>care</u>	Procedures and products added because of <u>the research</u>
**Treatment or Investigational products**	• Analgesics<br>• NSAIDs<br>• Symptomatic slow acting drugs for OA (SYSADOA)<br>• Knee joint injections	Resveratrol or Placebo
**Blood samples**	Not applicable	Not applicable
**Imaging, etc.**	Follow-up imaging (knee X-ray or magnetic resonance imaging)	Not applicable

4.8 Termination rules

Criteria and methods for prematurely terminating the research products. Any subject can withdraw from participating in the research at any time and for any reason. If a subject leaves the research prematurely, data relating to the subject can be used unless an objection was recorded when the subject signed the consent form. The investigator can temporarily or permanently prematurely terminate the research treatment for any reason that affects the subject's safety or which would be in the subject's best interests. If a subject prematurely terminates the research treatment, the subject will still be followed-up, and scheduled follow-up for the research will not be affected. If consent is withdrawn, no data about the subject may be used unless the subject states in writing that he/she does not object. In practice, the subject is excluded from the research. The investigator must: document the reason(s) and collect the assessment criteria when participation in the research ends, if the subject agrees.

Follow-up of the subjects after the premature termination of experimental products. Ending a subject's participation does not affect the normal management of the subject's illness in any way. If there are serious AEs (SAEs),, the investigator must notify the sponsor and monitor the subject until the end of the research. If treatment is stopped prematurely due to a SAE, a SAE notification form will be sent by fax (01 44 84 17 99) to the sponsor. The SAE will be monitored until it is resolved.

Methods for replacing subjects, if applicable. Subjects will not be replaced.

Terminating part or all of the research. AP-HP as sponsor or the Competent Authority (ANSM) can prematurely terminate all or part of the research, temporarily or permanently, in the following situations:

- First of all, if suspected unexpected serious adverse reactions (SUSARs) are seen in an arm receiving the resveratrol or if there is a discrepancy in the serious adverse reactions between the 2 arms, and which require a reassessment of the benefit-risk ratio for the research.
- Likewise, if unexpected facts, new information about the product, in light of which the objectives of the research are unlikely to be achieved.
- If it appears that the inclusion objectives are not met.

If the research is terminated prematurely, the decision and justification will be given by the sponsor, AP-HP, to the Competent Authority (ANSM) and to the CPP within 15 days.

5 ELIGIBILITY CRITERIA

5.1 Inclusion criteria

- Age ≥ 40 years-old
- Knee OA fulfilling 1986 ACR criteria
- Pain on NRS ≥ 40/100
- Symptom duration ≥ 1 month
- 4 > Kellgren and Lawrence X-Ray score ≥ 2
- No changes in the treatment in the past month
- Written consent obtained
- Health insurance cover

5.2 Exclusion criteria

- History of symptomatic crystal or inflammatory arthritis
- Knee surgery ≤ 1 year
- Knee trauma ≤ 2 months
- Knee intra-articular injections ≤ 2 months
- Neurologic disorders involving the lower limbs
- Inability to speak, write or read French language
- Participation to another biomedical research
- Contraindication to resveratrol or hypersensitivity to any of its constituents

5.3 Recruitment methods

Participants will be recruited by the physician among in- and out-patients and by the biomedical research technician on internet or phone among patients who would have replied to announcements made online, medias, and using posters displayed in each investigating centres, respectively.

	Number of subjects
Total number of subjects chosen	*164*
Number of centres	*3*
Inclusion period (months)	*24*
Number of subjects/centre	*54*
**Number of subjects/centre/month**	***2.3***

6 EXPERIMENTAL PRODUCTS ADMINISTERED TO RESEARCH PARTICIPANTS

6.1 Description of the experimental products

- **Resveratrol** is a dietary supplement, not a drug. Pharmaceutical dosage form used will be 20mg capsules. It will be administered orally, 30 minutes before meals with a glass of water with 2 capsules twice a day, for one week, then one capsule twice a day, for a total duration of 6 months. This dietary supplement is well tolerated and no marked toxicity has been reported [39]. Box containing 7 pillboxes of 60 capsules will be provided. Yvery Company will be in charge of labelling and blinding operations. Capsules should be stored in its original packaging at room temperature between 15 to 25°C. These capsules have already been available ont the French market for several years.
- **Placebo** of resveratrol will present with similar conditioning and taste. It will be administered orally, 30 minutes before meals with a glass of water with 2 capsules twice a day, for one week, then one capsule twice a day, for a total duration of 6 months. Box containing 7 pillboxes of 60 capsules will be provided. Yvery Company will be in charge of blinding and labelling operations. Capsules should be stored in its original packaging at room temperature between 15 to 25°C These capsules are already used in a PHRC currently conducted in neurology.

Supply of experimental products:

Yvery will supply patient boxes to the care units.

A stock of patient boxes will be send after the opening visit.

Specific prescription and automatic re supply will be generated by the e CRF

The investigator's brochure indicating the composition and the risks and toxicity of the experimental product will be provided to investigators.

6.2 Authorised and prohibited treatments (medicinal, non medicinal, surgical), including rescue medications

All treatments will be authorised during the study and there will be no prohibited treatments. Rescue medications (analgesics and NSAIDs) and joint injections (hyaluronic and corticosteroids) will be recorded in the CRF and assessed.

6.3 Methods for monitoring compliance with the experimental products

Patients will be asked to keep and return the medication bottles for capsule counts at the 3- and 6-month visits to monitor adherence. However, no specific measures will be taken to enhance adherence.

After accountability by the CRA, patient boxes will be send to Yvery for destruction.

7 ASSESSMENT OF EFFICACY

The primary assessment of efficacy criterion of the study is changes from baseline in mean knee pain intensity in the past 48 hours at 3 months. Mean knee pain intensity in the past 48 hours will be defined by the patient using a self-administered 11-point NRS and recorded at baseline and at 3-and 6-month follow-up **(Annex 1)**.

Secondary assessment of efficacy criteria will include:

- Change in knee pain at 6 months: defined as change from baseline in mean knee pain intensity in the last 48 hours at 6 months, using a self-administered 11-point NRS **(Annex 1)**.
- WOMAC physical function subscore at 3 and 6 months: defined as mean WOMAC physical function subscore at 3 and 6 months [34], using the French version of the questionnaire **(Annex 2).**
- Patient's global assessment of improvement since last assessment at 3 and 6 months assessed on an 11-point NRS (0 no improvement - 100 maximal improvement) **(Annex 3).**

- OARSI-OMERACT response at 3 and 6 months: defined as high improvement in pain or in function ≥ 50% and absolute change ≥ 20, or improvement in at least 2 of the 3 following: 1/ pain ≥ 20% and absolute change ≥ 10, 2/ function assessed by the WOMAC physical function subscore ≥ 20% and absolute change ≥ 10, 3/ patient's global assessment ≥ 20% and absolute change ≥ 10 at 3 and 6 months [35] **(Annex 4).**
- Self-reported analgesics and NSAIDs consumption, and corticoid and hyaluronic acid intra-articular injections since last contact at 3 and 6 months.

8 STATISTICAL ASPECTS

8.1 Calculation of sample size

The sample size is estimated at 164 patients. We have predicted a difference in mean change from baseline of 15 mm on the pain NRS between the experimental and the placebo groups, with a standard deviation of 27 mm, and a power of 90%, corresponding to 69 patients in each arm. Considering a 15% patient lost to follow-up, we have estimated that we will need to enrol a number of 82 patients for each arm. Fifteen points on pain NRS is considered as the minimal clinically perceived difference in pain for patients with knee OA.

8.2 Description of statistical methods to be used

All analyses will be performed on an intent-to-treat basis, in that all patients will be considered in the analysis and will be analyzed in the group to which they had been assigned. For descriptive analyses, qualitative variables will be reported with absolute and relative frequencies, and quantitative variables with median (interquartile range [IQR]). To compare differences in changes in values between the 2 treatment groups for quantitative variables, a constrained longitudinal data analysis will be used. In this model, both the baseline and post-baseline values will be modelled as dependent variables, and the true baseline means will be constrained to be the same for the 2 treatment groups. Hence, this analysis provides an adjustment for the observed baseline difference in estimating the treatment effects. The differences in differences from week 0 will be estimated at each time in each group by the time-by-treatment interaction. Random effects at patient and centre levels will be added to these models. Qualitative outcomes will be analysed using a mixed logistic regression model with a random effect at centre levels. Data analysis will involve use of SAS 9.4 (SAS Institute, Cary, NC). Blinded statisticians will perform the statistical analyses at an independent center (Centre d'Épidémiologie Clinique, Paris Descartes, Hôpital Hôtel-Dieu).

8.3 Statistical analysis plan

The statistical analysis described below will be further detailed in a dedicated Statistical Analysis Plan (SAP) before any analysis is undertaken.

9 SAFETY ASSESSMENT - RISKS AND RESTRICTIONS ADDED BY THE RESEARCH

9.1 Definitions

According to Article R1123-39 of the French Public Health Code

- **AE**
  Any untoward medical occurrence in a clinical trial subject and which does not necessarily have a causal relationship with the clinical trial or with the experimental product.

- **Adverse reaction**
  Any adverse event caused by the clinical trial.

- **SAE or adverse reaction**

Any **adverse event or reaction** which:

- Results in death,
- Is life threatening,
- Requires hospitalization or prolongation of existing hospitalization,
- Results in persistent or significant disability/incapacity,
- Is a congenital anomaly/birth defect,
- Any other event considered as "medically significant ".

- **Unexpected adverse reaction**

An adverse reaction whose nature, severity or outcome is not consistent with the available information about the product(s), act(s) or procedure(s) added by the clinical trial.

According to the notice to sponsors of clinical trials not conducted on health-product (ANSM):

- **New safety issue**

It is any new information about safety:

- That could significantly alter the assessment of the benefit-risk ratio for the clinical trial;
- Or which can lead to a modification the trial documents or can alter the conduct of the clinical trial.

For instance, it can be:

- A clinically significant increase in the frequency of occurrence of an expected serious adverse reaction;
- A premature termination or a temporary interruption due to safety reasons of a trial conducted in another country concerning the same product (procedure or methodology) as the trial conducted France.
- Recommendations from the Data Safety Monitoring Board (DSMB) that may affect the safety of the clinical trial subjects.
- Suspected unexpected serious adverse reaction (SUSAR) occurring in subjects who have completed the trial and which are notified to the sponsor by the investigator as well as the follow-up reports.

9.2 The investigator's roles

The investigator must notify the sponsor, immediately on the day when the sponsor becomes aware, of all the SAE, except those that are listed in the protocol (see. section 9.3) or in the investigator's brochure as not requiring immediate notification.

These SAE are recorded in the "adverse event" section of the CCRF and the investigator must immediately notify the sponsor's Vigilance division (see 9.4).

The investigator's other roles

The investigator must document the SAE as thoroughly as possible and provide the medical diagnosis, if possible using a specific SAE form **(Annex 5)**.

The investigator assesses the severity of the AE:

- *Mild: tolerated by the patient, does not interfere with daily activities*
- *Moderate: sufficiently uncomfortable to affect daily activities*
- *Serious: preventing daily activities*

The investigator must **assess the causality relationship** between the SAEs and the clinical trial.

The method used by the investigator is based on the WHO method (WHO Uppsala Monitoring Centre), and includes the following four causality terms:

- Certain
- Probable/Likely
- Possible
- Unlikely (not excluded)

Their definition is provided in the table below (from WHO-UMC causality categories, version from 17-Apr-2012).

Table WHO-UMC causality categories (extract)

Causality term	Assessment criteria*
**Certain**	· Event or laboratory test abnormality, with plausible time relationship to drug intake<br>· Cannot be explained by disease or other drugs<br>· Response to withdrawal plausible (pharmacologically, pathologically)<br>· Event definitive pharmacologically or phenomenologically (i.e. an objective and specific medical disorder or a recognized pharmacological phenomenon)<br>· Rechallenge satisfactory, if necessary
**Probable / Likely**	· Event or laboratory test abnormality, with reasonable time relationship to drug intake<br>· Unlikely to be attributed to disease or other drugs<br>· Response to withdrawal clinically reasonable<br>· Rechallenge not required
**Possible**	· Event or laboratory test abnormality, with reasonable time relationship to drug intake<br>· Could also be explained by disease or other drugs<br>· Information on drug withdrawal may be lacking or unclear
**Unlikely**	· Event or laboratory test abnormality, with a time to drug intake<br>· that makes a relationship improbable (but not impossible)<br>· Disease or other drugs provide plausible explanations

*All points should be reasonably complied with

9.3 Specific features of the protocol

All serious and non-serious AEs are collected using an open-ended question and must be reported in the dedicated section of the CRF.

SAEs that do not require the investigator to immediately notify the sponsor

These SAEs are only recorded in the "adverse event" section of the eCRF. They include the events associated with:

- Normal and natural evolution of the pathology, for example:
– Scheduled medical visit for the follow-up of knee OA
– Scheduled hospitalization for the routine treatment of knee OA(joint injection, rehabilitation), and not related to a worsening of the condition
– Expected symptoms secondary to knee OA worsening : joint pain, joint effusion, OA flare, walking difficulties, surgical knee joint replacement for OA

- Special circumstances
– Hospitalisation for pre-existing condition
– Hospitalization for intervention or surgery hospitalisation scheduled prior to the research
– Hospital admission for social or administrative purpose
– Admission to the emergency room less than 12 hrs

- AEs likely to be associated with the treatments prescribed as part of the patient's care during the monitoring of the research,
    - AE related to rescue medications (analgesics and NSAIDs)
    - AE related to joint injections (hyaluronic and corticosteroids): increased pain, joint swelling, and mild joint effusion that can last a few days, and skin flush following corticosteroid injections that can last a few hours. Exceptionnally, septic arthritis or allergic reaction.

These adverse reactions should be reported by the investigator to the regional pharmacovigilance center (CRPV) of which it depends.

SAEs that require the investigator to immediately notify the sponsor

The investigator must report all AEs that meet one of the seriousness criteria below:
1- Death
2- Life threatening situation
3- Requiring hospitalisation or prolonging hospitalisation
4- Persistent or significant disability or incapacity
5- Congenital abnormality or birth defect
6- Or any other AE considered "medically significant"

9.4 Procedures and deadlines for notifying the sponsor

Notification of an SAE must initially be provided in a written report using the special form for reporting SAE. The report must be signed by the investigator.

Each item in the form must be completed by the investigator so that the sponsor can carry out the appropriate analysis.

This initial notification must be followed by one or more detailed follow-up report(s), in writing and signed, within a maximum of 8 days in the case of a fatal or life-threatening event and within 15 days for all other cases.

Whenever possible, the investigator will provide the sponsor with any documents that may be useful (medical reports, laboratory test results, results of additional exams, etc.). These documents must be made anonymous. In addition, the documents must include the following: research acronym, number and initials of the subject, nature and date of the SAE.

Any AE will be monitored until fully resolved (stabilisation at a level considered acceptable by the investigator, or return to the previous state) even if the subject has left the trial.

The initial notification, the SAE follow-up reports and all other documents must be sent to the sponsor via fax only to the Vigilance Division of the DRCD, fax No. **01 44 84 17 99**.

For this study using e-CRF:

- the investigator completes the SAE notification form in the e-CRF, validates, prints and signs the form before sending it *via* fax.
- if it is not possible to connect to the e-CRF, the investigator will complete, sign and send the SAE notification form**.** As soon as the connection is restored, the SAE notification form in the e-CRF must be duly completed.

The investigator must comply with all requests from the sponsor for additional information.

For all questions relating to the notification of an AE, the Vigilance Division of the DRCD can be contacted via email: vigilance.drc@aphp.fr

9.5 Period for notifying the sponsor

The investigator must report all SAE that occur in research subjects:

- on the date ofthe first administration of investigational product
- throughout the period during which the participant is monitored, as determined by the research

9.6 The sponsor's roles

The sponsor represented by the Vigilance department continuously assesses the safety of the clinical trial throughout the duration of the clinical trial.

Analysis and declaration of SAEs

The sponsor is responsible for the assessment of the following:

- the **seriousness** of all AEs reported,
- the **causality relationship** between the SAE and the acts/procedures/tests added by the clinical trial,
  All SAEs considered by the investigator and/or the sponsor to be possibly related to the act/procedures/tests/products administered, specific to the clinical trial can be reasonably considered as suspected adverse reaction.
- **Expectedness or unexpectedness** of adverse reactions.

  Any adverse reaction whose nature, severity or outcome is not consistent with the information relative to the acts/procedures/ and or products administered during the clinical study is considered unexpected.

  The assessment of the expected/unexpected nature of an adverse is performed by the sponsor represented by the Vigilance department. This assessment is done according the information described below

**Unlikely AEs related** to resveratrol have been reported: headache, myalgia of the lower extremities, somnolence, epidymitis, dizziness, nasopharyngitis, and erythematous rash. Neprotoxicity was reported in *in vivo* animal studies.
Refer to:<br>- Dossier Technique Investigateur provided to investigators.<br>- Charles-Henry Cottart and al. *Resveratrol bioavailability and toxicity in humans*. Mol. Nutr. Food Res. 2010, 54, 1-10

The sponsor declares any suspected unexpected serious adverse reaction (SUSAR), within the legal deadline, to the Agence nationale de sécurité du médicament et des produits de santé (ANSM, French Health Products Safety Agency) and the concerned Comité de Protection des Personnes (CPP, ethical commitee):

- The initial report must be made within 7 calendar days from the date of receipt of the SAE.
- All complementary information must be reported by the sponsor in the form of follow-up reports, within a period of 7 calendar days from the date of receipt of the complementary information.

The sponsor must inform all the concerned investigators of any data which could affect the safety of the participants.

Special case of double-blinded studies:

After unblinding, if the administered product is the investigational product, the case will be immediately declared as suspected serious unexpected adverse reaction (SUSAR); however, if the administered product is the comparator, the unexpected nature of the adverse reaction will be re-assessed according to the reference document of the comparator found in the protocol. In the exceptional case of a clinical study about a disease with high mortality or morbidity, during the

clinical study application, the sponsor may request to the ANSM a readjustment of the conditions for unblinding and reporting suspected adverse reaction.

Analysis and declaration of other safety data

This relates to any safety data or new safety issue that could significantly alter the assessment of the benefit-risk ratio of the clinical trial, or could lead to modification in the conduct of the study.

The reporting of safety issues to the competent authorities must be carried out by the sponsor without delay and at the latest within seven calendar days following the day of knowledge.
Following the initial reporting of a new safety issue, the sponsor must send follow-up reports for any relevant additional information relative to this safety issue at the latest within seven calendar days following the day of knowledge.

Annual safety report

The sponsor must prepare once yearly throughout the duration of the clinical trial an annual safety report (ASR) that includes:
- an analysis of participants' safety,
- a list of all suspected serious adverse reactions for the concerned trial that occurred in France during the covered period of the report,
- summary tables of all the SAEs which have occurred in the trial concerned.

Special case: for trials that pose minor risks, that have no influence on subjects' medical care, that do not involve the administration of an investigational product or the practice of an experimental act, the safety report is provided in a simplified letter. The simplified letter of the annual safety report contains a list of suspected adverse reactions that occurred during the covered period of the report, a global analysis and an updated assessment of benefits and risks of the clinical trial.

The annual safety report must be submitted within 60 days after the birth date corresponding to the date of inclusion of the first trial subject.

9.7 Data Safety Monitoring Board (DSMB)

The DSMB can be established by the sponsor. Its primary mission is to serve as a committee for monitoring safety data. It can have other missions, such as monitoring efficacy data (especially if the protocol includes interim analyses). The DSMB is mentioned in Article L. 1123-7 of the French Public Health Code.

A Data and Safety Monitoring Board (DSMB) will not be convened for the present biomedical research

10 DATA MANAGEMENT

10.1 Data collection methods

Information required in the research protocol must be collected in the CRF and an explanation must be given by the investigator for each missing data.
Data must be reported in the electronic CRF when they are available, for clinical or para-clinical data. Correction of discordant data on CRF will be asked through queries. In the CRF, the changes in the data will be tracked.

Anonymization of the patients will ensured using a code number and initials, reported on each needed documents for the research, or by erasing nominative data on copies of source documents.

10.2 Right to access source data and documents

Access to data

In accordance with GCPs:
- the sponsor is responsible for obtaining the permission of all parties involved in the research to guarantee direct access to all locations where the research will be carried out, to the source data, to the source documents and the reports, with the goal of quality control and audit by the sponsor
- the investigators will make available to those in charge of monitoring, quality control and audit relating to the biomedical research the documents and personal data strictly necessary for these controls, in accordance with the legislative and regulatory provisions in force (Articles L.1121-3 and R.5121-13 of the French Public Health Code)

Source documents

Source documents are defined as any original document or object that can prove the existence or accuracy of a piece of information or a fact recorded during the research. These documents will be kept for 15 years by the investigator or by the hospital in the case of a hospital medical file.

Data confidentiality

Those responsible for biomedical research quality control (Article L.1121-3 of the French Public Health Code) will take all necessary precautions to ensure the confidentiality of information about the experimental medications, the research, the research subjects and in particular the identity of the subjects and the results obtained.

These individuals, as well as the investigators themselves, are subject to professional secrecy (in accordance with the conditions set out in Articles 226-13 and 226-14 of the Penal Code).
During or after the biomedical research, the data collected about the research subjects and sent to the sponsor by the investigators (or any other specialised parties) will be made non-identifying.
Under no circumstances should the names and addresses of the subjects involved be shown.

The sponsor will ensure that each research subject has given permission in writing for access to personal information about him or her which is strictly necessary for the quality control of the research.

10.3 Data processing and storage of documents and data

Data entry

Data entry will be carried out on an eCRF, filled in on the internet after each visit by the investigator-physicians in each centre. Access to the on-line data entry form by the investigator-physicians will be restricted by an access code and a personal and unique password system for each user. Each investigator will, in addition, have access to a specific profile that attributes or withholds access to certain functions of the system (entering data, or simply viewing the data of the enrolled patient or all the study data, possibility of change and validation by the CRAs, etc.…). Data will be stored on a secure server, with data encrypted during transmission and automatic internal saving of a copy on the server that will host the eCRF.

Data processing (CNIL, the French Data Protection Authority) in France

This research falls under the "Méthodologie de référence" (MR-001) according to the provisions of Article 54, paragraph 5 of modified Law No. 78-17 of 6 January 1978 relating to information technology, data files and privacy. This change was approved in a decision made on 5 January 2006. AP-HP, the research sponsor, has signed a commitment to comply with this "Méthodologie de référence".

Archival

Specific documents for biomedical research will be archived by the investigator and the sponsor for a period of 15 years after the end of the research.

10.4 Ownership of the data

AP-HP is the owner of the data, which cannot be used or disclosed to a third party without its prior approval.

11 QUALITY CONTROL AND ASSURANCE

Each biomedical research project managed by AP-HP is ranked from A to D according to the projected risk incurred by research subjects using the classification of biomedical research sponsored by AP-HP.

11.1 General organisation

The sponsor must be responsible for the safety and respect of those subjects who have agreed to participate in the research. The sponsor must implement a quality assurance system to best monitor the conduct of the research in the investigation centres.

For this purpose, the sponsor shall delegate Clinical Research Associates (CRA) whose primary role is to carry out regular follow-up visits at the research locations, after having carried out initial visits.

The objective of monitoring the research, as defined in the French Good Clinical Practices (BPC section 5.18.1), are to verify that:

- The rights, safety and protection of the research subjects are met,
- The data reported is exact, complete and consistent with the source documents,
- The research is carried out in accordance with the protocol in force, with French GCPs and with the legislative and regulatory provisions in force.

11.2 Level of centre monitoring

In the case of this research, which is considered A risk, the appropriate monitoring level will be determined based on the complexity, the impact and the budget for the research. Thus, the sponsor and the coordinating investigator have agreed on the logistic score and impact, resulting in a research monitoring level to be implemented.

11.3 Quality control

A Clinical Research Associate (CRA) appointed by the sponsor will be responsible for the proper conduct of the research, for collecting and documenting, recording and reporting the data generated in writing, in accordance with the Standard Operating Procedures applied within the DRCD and in accordance with the French Good Clinical Practices as well as with the legislative and regulatory provisions in force.

The investigator and the members of the investigator's team agree to make themselves available during Quality Control visits carried out at regular intervals by the Clinical Research Associate. During these visits, the following elements will be reviewed:

- written consent
- compliance with the research protocol and with the procedures defined therein
- quality of the data collected in the CRF: accuracy, missing data, consistency of the data with the "source" documents (medical files, appointment books, original copies of laboratory results, etc.)
- management of the treatments used

11.4 Case Report Form (CRF)

All information required according to the protocol must be entered in the CRF. The data must be collected as and when they are obtained, and clearly recorded in these CRF. Each missing data item must be coded.

This digital CRF will be implemented in each of the centres thanks to a web-based data collection medium. Investigators will be given a document offering guidance in using this tool.
When the investigators complete the case report via the Internet, the CRA can view the data quickly and remotely. The investigator is responsible for the accuracy, quality and relevance of all the data entered. In addition, the data are immediately verified as they are entered, thanks to consistency checks. Thus, the investigator must validate any changes to the values in the CRF. These modifications will be subject to an audit trail. A justification can be added when applicable, as a comment. A print-out, authenticated (signed and dated) by the investigator, will be requested at the end of the research. The investigator must archive a copy of the authenticated document that was delivered to the sponsor.

11.5 Management of non-compliances

Any events that occur as a result of non-compliance, by the investigator or any other individual involved in conducting the research, with the protocol, with the standard operating procedures, with the good clinical practices or with the legislative and regulatory provisions in force must be noted in a declaration of non-compliance addressed to the sponsor. As a first step, major or critical non-compliances will be reviewed and processed by the DRCD's medical coordinator in order to implement the necessary corrective or preventive actions. Next, the non-compliances will be sent to the Quality - Risk Management Division of the DRCD for verification and analysis. These verifications could result in the investigator in charge of the research location in question being asked for information or could lead to compliance or audit visits.

11.6 Audits/inspections

The investigators agree to accept the quality assurance audits carried out by the sponsor as well as the inspections carried out by the competent authorities. All data, documents and reports may be subject to regulatory audits and inspections. Medical secrecy cannot be invoked in opposition to these audits and inspections.

An audit can be carried out at any time by individuals appointed by the sponsor and who are not associated with the research directors. The objective of the audit is to ensure the quality of the research, the validity of the results and compliance with the legislation and regulations in force.
The individuals who lead and monitor the research agree to comply with the sponsor's requirements and with the competent authority regarding research audits or inspections.

The audit may be applicable to all stages of the research, from the development of the protocol to the publication of the results and the organisation of the data used or produced as part of the research.

11.7 Primary investigator's commitment to assume responsibility

Before starting the research, each investigator will give the sponsor’s representative a copy of his/her personal curriculum vitæ, signed and dated, with his/her number in the RPPS (*Répertoire Partagé des Professionnels de Santé*, Collective Database of Health Professionals).

Each investigator will undertake to comply with the legislation and to carry out the research according to French GCP, adhering to the Declaration of Helsinki terms in force.

The primary investigator at each participating centre will sign a responsibility commitment (standard DRCD document) which will be sent to the sponsor's representative.

The investigators and their employees will sign a delegation of duties form specifying each person's role.

12 ETHICAL AND LEGAL CONSIDERATIONS

12.1 Methods for obtaining information and consent from research participants

In accordance with Article L1122-1-1 of the French Public Health Code, no biomedical research can be carried out on a person without free and informed consent, obtained in writing after the person has been given the information specified in Article L.1122-1 of said Code.

The free and informed consent, in writing, of the subject is obtained by the investigator, or by a doctor representing the investigator, before the inclusion of the subject in the research.
The information sheet and a copy of the consent form signed and dated by the research subject and by the investigator or the doctor representing the investigator, are given to the individual prior to his or her participation in the research.

In addition, the investigator will specify in the research participant's medical file the methods used for obtaining his or her consent as well as the methods used for providing information with the

goal of obtaining their consent. The investigator will retain the original signed and dated copy of the subject's consent form.

12.2 Subject prohibited from participating in another research or an exclusion period anticipated after the research, if applicable

The subject will be prohibited from participating in other biomedical research protocols relating to medications during the whole study duration, in order to avoid interferences with the results of the study. There will be no exclusion period anticipated after the research.

12.3 Compensation for subjects

No compensation is anticipated for the patients/control subjects as compensation for the inconveniences relating to the research.

12.4 Legal obligations

The sponsor's role

Assistance Publique - Hôpitaux de Paris (AP-HP) is the sponsor of this research and by delegation, the Clinical Research and Development Department (DRCD) carries out the research's missions in accordance with Article L.1121-1 of the French Public Health Code. Assistance Publique - Hôpitaux de Paris reserves the right to halt the research at any time for medical or administrative reasons. In this case, notification will be sent to the investigator.

Request for an opinion from the Comité de Protection des Personnes (CPP, ethical review board)

AP-HP, as sponsor, obtains for this biomedical research relating to a medication for human use and prior to starting the research, the favourable opinion of the appropriate CPP, within the scope of its authority and in accordance with the legislative and regulatory provisions in force.

Request for authorisation to ANSM

AP-HP, as sponsor, obtains for this biomedical research relating to a medication for human use and prior to starting the research, authorisation from the ANSM, within the scope of its authority and in accordance with the legislative and regulatory provisions in force.

Commitment to compliance with the MR 001 "Méthodologie de Reference"

AP-HP, the research sponsor, has signed a commitment to comply with this "Méthodologie de reference".

Request for the opinion of the CCTIRS (advisory committee on the processing of research information in the area of health) and request for authorisation from CNIL (French data protection authorities)

As the processing of personal data for this research does not fall under the scope of the MR 001 méthodologie de reference, the sponsor must obtain the opinion of the CCTIRS and the authorisation of the CNIL.

Standard declaration to the CNIL

AP-HP as sponsor of the research will make a standard declaration to the CNIL.

12.5 Modifications to the research

Any substantial modification to the protocol by the coordinating investigator must be sent to the sponsor for approval. After approval is given, the sponsor must obtain, prior to starting the research, a favourable opinion from the CPP and authorisation from the ANSM within the scope of their respective authorities.

The information sheet and the consent form can be revised if necessary; in particular if there is a substantial modification to the research or if adverse reactions occur.

12.6 Final research report

The final biomedical research report referred to in Article R1123-60 of the French Public Health Code is drawn up and signed by the sponsor and the investigator. A summary of the report written according to the competent authority's reference plan will need to be sent to the competent authority and ethical review board within one year after the end of the research, meaning the end of the participation of the last research subject.

13 FUNDING AND INSURANCE

13.1 Funding source

The research is funded by the 2015 Programme Hospitalier de Recherche Clinique (grant n° 15-15-0234 of the French Ministry of Health).

13.2 Insurance

For the duration of the research, the Sponsor will take out an insurance policy covering the sponsor's own civil liability as well as the civil liability of all the doctors involved in carrying out the research. The sponsor will also provide full compensation for all harmful consequences of the research for the research subjects and their beneficiaries, unless the sponsor can prove that the harm is not the fault of the sponsor or any agent. The act of a third party or the voluntary withdrawal of the person who initially consented to participate in the research cannot be invoked against said compensation.

Assistance Publique- Hôpitaux de Paris (AP-HP) has taken out insurance from HDI-GERLING through BIOMEDIC-INSURE for the full research period, covering its own civil liability and that of any agent (doctor or research staff), in accordance with Article L.1121-10 of the French Public Health Code.

14 PUBLICATION RULES

14.1 Mention of the AP-HP manager (DRCD) in the acknowledgements of the text

"The sponsor was Assistance Publique - Hôpitaux de Paris (Département de la Recherche Clinique et du Développement, Clinical Research and Development Department)"

14.2 Mention of the financier in the acknowledgements of the text

The following statement will be included in all publications “The research was funded by a grant from Programme Hospitalier de Recherche Clinique - PHRC 2015 (Ministère de la Santé)”.

This research has been registered on the website http://clinicaltrials.gov: (ClinicalTrials.gov Identifier: NCT02905799; first received: September 14, 2016; last updated: September 16, 2016).

15 BIBLIOGRAPHY

1. DALYs GBD, Collaborators H, Murray CJ, Barber RM, Foreman KJ, Ozgoren AA, et al. Global, regional, and national disability-adjusted life years (DALYs) for 306 diseases and injuries and healthy life expectancy (HALE) for 188 countries, 1990-2013: quantifying the epidemiological transition. Lancet. 2015. doi: 10.1016/S0140-6736(15)61340-X. PubMed PMID: 26321261.
2. Bijlsma JW, Berenbaum F, Lafeber FP. Osteoarthritis: an update with relevance for clinical practice. Lancet. 2011;377(9783):2115-26. doi: 10.1016/S0140-6736(11)60243-2. PubMed PMID: 21684382.

3. Palazzo C, Ravaud JF, Papelard A, Ravaud P, Poiraudeau S. The burden of musculoskeletal conditions. PLoS One. 2014;9(3):e90633. doi: 10.1371/journal.pone.0090633. PubMed PMID: 24595187; PubMed Central PMCID: PMC3942474.

4. Richette P, Latourte A, Frazier A. Safety and efficacy of paracetamol and NSAIDs in osteoarthritis: which drug to recommend? Expert Opin Drug Saf. 2015;14(8):1259-68. doi: 10.1517/14740338.2015.1056776. PubMed PMID: 26134750.

5. Roberts E, Delgado Nunes V, Buckner S, Latchem S, Constanti M, Miller P, et al. Paracetamol: not as safe as we thought? A systematic literature review of observational studies. Ann Rheum Dis. 2015. doi: 10.1136/annrheumdis-2014-206914. PubMed PMID: 25732175.

6. Vang O, Ahmad N, Baile CA, Baur JA, Brown K, Csiszar A, et al. What is new for an old molecule? Systematic review and recommendations on the use of resveratrol. PLoS One. 2011;6(6):e19881. doi: 10.1371/journal.pone.0019881. PubMed PMID: 21698226; PubMed Central PMCID: PMC3116821.

7. Amiot MJ, Romier B, Dao TM, Fanciullino R, Ciccolini J, Burcelin R, et al. Optimization of trans-Resveratrol bioavailability for human therapy. Biochimie. 2013;95(6):1233-8. doi: 10.1016/j.biochi.2013.01.008. PubMed PMID: 23376875.

8. Renaud S, Ruf JC. The French paradox: vegetables or wine. Circulation. 1994;90(6):3118-9. Epub 1994/12/01. PubMed PMID: 7994864.

9. Constant J. Alcohol, ischemic heart disease, and the French paradox. Coronary artery disease. 1997;8(10):645-9. Epub 1998/02/11. PubMed PMID: 9457446.

10. Soleas GJ, Diamandis EP, Goldberg DM. Resveratrol: a molecule whose time has come? And gone? Clinical biochemistry. 1997;30(2):91-113. Epub 1997/03/01. PubMed PMID: 9127691.

11. Fremont L. Biological effects of resveratrol. Life sciences. 2000;66(8):663-73. Epub 2000/02/19. PubMed PMID: 10680575.

12. Jang M, Cai L, Udeani GO, Slowing KV, Thomas CF, Beecher CW, et al. Cancer chemopreventive activity of resveratrol, a natural product derived from grapes. Science (New York, NY). 1997;275(5297):218-20. Epub 1997/01/10. PubMed PMID: 8985016.

13. Smoliga JM, Baur JA, Hausenblas HA. Resveratrol and health--a comprehensive review of human clinical trials. Mol Nutr Food Res. 2011;55(8):1129-41. doi: 10.1002/mnfr.201100143. PubMed PMID: 21688389.

14. Cottart CH, Nivet-Antoine V, Beaudeux JL. Is resveratrol an imposter? Mol Nutr Food Res. 2015;59(1):7. doi: 10.1002/mnfr.201570005. PubMed PMID: 25558005.

15. Visioli F. The resveratrol fiasco. Pharmacol Res. 2014;90:87. doi: 10.1016/j.phrs.2014.08.003. PubMed PMID: 25180457.

16. Yang FM, Friedrichs WE, Cupples RL, Bonifacio MJ, Sanford JA, Horton WA, et al. Human ceruloplasmin. Tissue-specific expression of transcripts produced by alternative splicing. J Biol Chem. 1990;265(18):10780-5. PubMed PMID: 2355023.

17. Dave M, Attur M, Palmer G, Al-Mussawir HE, Kennish L, Patel J, et al. The antioxidant resveratrol protects against chondrocyte apoptosis via effects on mitochondrial polarization and ATP production. Arthritis Rheum. 2008;58(9):2786-97. doi: 10.1002/art.23799. PubMed PMID: 18759268.

18. Li X, Phillips FM, An HS, Ellman M, Thonar EJ, Wu W, et al. The action of resveratrol, a phytoestrogen found in grapes, on the intervertebral disc. Spine (Phila Pa 1976). 2008;33(24):2586-95. doi: 10.1097/BRS.0b013e3181883883. PubMed PMID: 19011540; PubMed Central PMCID: PMC2905225.

19. Malemud CJ. Inhibitors of stress-activated protein/mitogen-activated protein kinase pathways. Curr Opin Pharmacol. 2007;7(3):339-43. doi: 10.1016/j.coph.2006.11.012. PubMed PMID: 17398158.

20. Mengshol JA, Vincenti MP, Coon CI, Barchowsky A, Brinckerhoff CE. Interleukin-1 induction of collagenase 3 (matrix metalloproteinase 13) gene expression in chondrocytes requires p38, c-Jun N-terminal kinase, and nuclear factor kappaB: differential regulation of collagenase 1 and collagenase 3. Arthritis Rheum. 2000;43(4):801-11. doi: 10.1002/1529-0131(200004)43:4<801::AID-ANR10>3.0.CO;2-4. PubMed PMID: 10765924.

21. Shakibaei M, Mobasheri A, Buhrmann C. Curcumin synergizes with resveratrol to stimulate the MAPK signaling pathway in human articular chondrocytes in vitro. Genes Nutr. 2011;6(2):171-9. doi: 10.1007/s12263-010-0179-5. PubMed PMID: 21484156; PubMed Central PMCID: PMC3092909.

22. Csaki C, Mobasheri A, Shakibaei M. Synergistic chondroprotective effects of curcumin and resveratrol in human articular chondrocytes: inhibition of IL-1beta-induced NF-kappaB-mediated inflammation and apoptosis. Arthritis Res Ther. 2009;11(6):R165. doi: 10.1186/ar2850. PubMed PMID: 19889203; PubMed Central PMCID: PMC3003513.

23. Lei M, Liu SQ, Liu YL. Resveratrol protects bone marrow mesenchymal stem cell derived chondrocytes cultured on chitosan-gelatin scaffolds from the inhibitory effect of interleukin-1beta. Acta Pharmacol Sin. 2008;29(11):1350-6. doi: 10.1111/j.1745-7254.2008.00880.x. PubMed PMID: 18954530.

24. Liu FC, Hung LF, Wu WL, Chang DM, Huang CY, Lai JH, et al. Chondroprotective effects and mechanisms of resveratrol in advanced glycation end products-stimulated chondrocytes. Arthritis Res Ther. 2010;12(5):R167. doi: 10.1186/ar3127. PubMed PMID: 20825639; PubMed Central PMCID: PMC2990994.

25. Shakibaei M, Csaki C, Nebrich S, Mobasheri A. Resveratrol suppresses interleukin-1beta-induced inflammatory signaling and apoptosis in human articular chondrocytes: potential for use as a novel nutraceutical for the treatment of osteoarthritis. Biochem Pharmacol. 2008;76(11):1426-39. doi: 10.1016/j.bcp.2008.05.029. PubMed PMID: 18606398.

26. Tian J, Chen JW, Gao JS, Li L, Xie X. Resveratrol inhibits TNF-alpha-induced IL-1beta, MMP-3 production in human rheumatoid arthritis fibroblast-like synoviocytes via modulation of

PI3kinase/Akt pathway. Rheumatol Int. 2013;33(7):1829-35. doi: 10.1007/s00296-012-2657-0. PubMed PMID: 23328930.

27. Byun HS, Song JK, Kim YR, Piao L, Won M, Park KA, et al. Caspase-8 has an essential role in resveratrol-induced apoptosis of rheumatoid fibroblast-like synoviocytes. Rheumatology (Oxford). 2008;47(3):301-8. doi: 10.1093/rheumatology/kem368. PubMed PMID: 18276737.

28. Ferrero ME, Bertelli AE, Fulgenzi A, Pellegatta F, Corsi MM, Bonfrate M, et al. Activity in vitro of resveratrol on granulocyte and monocyte adhesion to endothelium. Am J Clin Nutr. 1998;68(6):1208-14. PubMed PMID: 9846848.

29. Vorderstrasse BA, Steppan LB, Silverstone AE, Kerkvliet NI. Aryl hydrocarbon receptor-deficient mice generate normal immune responses to model antigens and are resistant to TCDD-induced immune suppression. Toxicol Appl Pharmacol. 2001;171(3):157-64. doi: 10.1006/taap.2000.9122. PubMed PMID: 11243915.

30. Elmali N, Esenkaya I, Harma A, Ertem K, Turkoz Y, Mizrak B. Effect of resveratrol in experimental osteoarthritis in rabbits. Inflamm Res. 2005;54(4):158-62. doi: 10.1007/s00011-004-1341-6. PubMed PMID: 15883738.

31. Wang J, Gao JS, Chen JW, Li F, Tian J. Effect of resveratrol on cartilage protection and apoptosis inhibition in experimental osteoarthritis of rabbit. Rheumatol Int. 2012;32(6):1541-8. doi: 10.1007/s00296-010-1720-y. PubMed PMID: 21327438.

32. Li W, Cai L, Zhang Y, Cui L, Shen G. Intra-articular resveratrol injection prevents osteoarthritis progression in a mouse model by activating SIRT1 and thereby silencing HIF-2alpha. J Orthop Res. 2015;33(7):1061-70. doi: 10.1002/jor.22859. PubMed PMID: 25737402.

33. Bellamy N, Kirwan J, Boers M, Brooks P, Strand V, Tugwell P, et al. Recommendations for a core set of outcome measures for future phase III clinical trials in knee, hip, and hand osteoarthritis. Consensus development at OMERACT III. The Journal of rheumatology. 1997;24(4):799-802. PubMed PMID: 9101522.

34. Bellamy N, Buchanan WW, Goldsmith CH, Campbell J, Stitt LW. Validation study of WOMAC: a health status instrument for measuring clinically important patient relevant outcomes to antirheumatic drug therapy in patients with osteoarthritis of the hip or knee. The Journal of rheumatology. 1988;15(12):1833-40. PubMed PMID: 3068365.

35. Pham T, van der Heijde D, Altman RD, Anderson JJ, Bellamy N, Hochberg M, et al. OMERACT-OARSI initiative: Osteoarthritis Research Society International set of responder criteria for osteoarthritis clinical trials revisited. Osteoarthritis and cartilage / OARS, Osteoarthritis Research Society. 2004;12(5):389-99. doi: 10.1016/j.joca.2004.02.001. PubMed PMID: 15094138.

36. Ware J, Jr., Kosinski M, Keller SD. A 12-Item Short-Form Health Survey: construction of scales and preliminary tests of reliability and validity. Medical care. 1996;34(3):220-33. PubMed PMID: 8628042.

37. Conrozier T, Mathieu P, Vignon E, Piperno M, Rinaudo M. Differences in the osteoarthritic synovial fluid composition and rheology between patients with or without flare: a pilot study. Clinical and experimental rheumatology. 2012;30(5):729-34. PubMed PMID: 22935334.
38. Schulz KF, Altman DG, Moher D, Group C. CONSORT 2010 statement: updated guidelines for reporting parallel group randomised trials. Bmj. 2010;340:c332. doi: 10.1136/bmj.c332. PubMed PMID: 20332509; PubMed Central PMCID: PMC2844940.
39. Cottart CH, Nivet-Antoine V, Laguillier-Morizot C, Beaudeux JL. Resveratrol bioavailability and toxicity in humans. Mol Nutr Food Res. 2010;54(1):7-16. doi: 10.1002/mnfr.200900437. PubMed PMID: 20013887.

16 LIST OF ADDENDA

16.1 List of Investigators

Address of the research location	Title	First name Surname	Telephone / e-mail / Fax
Service de Rééducation et de Réadaptation de l'Appareil Locomoteur et des Pathologies du Rachis<br>Hôpitaux Universitaires Paris Centre<br>Groupe Hospitalier Cochin<br>27, Rue du Faubourg Saint-Jacques<br>75014 Paris, FRANCE	MD, PhD	Prof. François Rannou	Tel: +33158412535<br>francois.rannou@aphp.fr<br>Fax: +33158412545
Service de Rhumatologie<br>Hôpitaux Universitaires Est Parisien<br>Groupe Hospitalier Saint-Antoine<br>184, Rue du Faubourg Saint-Antoine<br>75012 Paris, FRANCE	MD, PhD	Prof. Francis Berenbaum	Tel: +33149282520<br>francis.berenbaum@aphp.fr<br>Fax: +33149282513
Service de Médecine Physique et de Réadaptation<br>Université d'Auvergne<br>CHU Clermont-Ferrand<br>CHU Hôpital Nord<br>61, Rue de Chateaugay<br>BP 30056, 63118 Cébazat, FRANCE	MD, PhD	Prof. Emmanuel Coudeyre	Tel: +33473750900<br>ecoudeyre@chu-clermontferrand.fr<br>Fax: +33473750901

16.2 Annexes

Annex 1. Pain numeric scale

Échelle numérique pour l'auto-évaluation de la douleur

Cette échelle numérique a pour but d'évaluer **la douleur ressentie au niveau du genou** au cours de **ces dernières 48h**. Pour cela, il suffit de cocher une case sur l'échelle selon la douleur ressentie.

- Évaluation de la douleur **au niveau du genou** au cours de **ces dernières 48h** :

Aucune douleur **Douleur insupportable**

☐	☐	☐	☐	☐	☐	☐	☐	☐	☐	☐
**0**	**10**	**20**	**30**	**40**	**50**	**60**	**70**	**80**	**90**	**100**

Annex 2. French version of the WOMAC physical function subscore questionnaire [34]

Pour chacune des activités suivantes, veuillez préciser les difficultés éprouvées en raison de votre articulation souffrante, **au cours des dernières 48 heures**.

SECTION FONCTION						SECTION PERSONNALISÉE
Quelle est l'importance de la difficulté que vous éprouvez à :	**Aucune**<br>**0**	**Minime**<br>**1**	**Modérée**<br>**2**	**Sévère**<br>**3**	**Très sévère**<br>**4**	**Cochez les 5 Items choisis par le patient**
1. Descendre les escaliers	❑	❑	❑	❑	❑	❑
2. Monter les escaliers	❑	❑	❑	❑	❑	❑
3. Vous relever de la position assise	❑	❑	❑	❑	❑	❑
4. Vous tenir debout	❑	❑	❑	❑	❑	❑
5. Vous pencher en avant	❑	❑	❑	❑	❑	❑
6. Marcher en terrain plat	❑	❑	❑	❑	❑	❑
7. Entrer et sortir d'une voiture	❑	❑	❑	❑	❑	❑
8. Faire vos courses	❑	❑	❑	❑	❑	❑
9. Enfiler vos collants ou vos chaussettes	❑	❑	❑	❑	❑	❑
10. Sortir du lit	❑	❑	❑	❑	❑	❑
11. Enlever vos collants ou vos chaussettes	❑	❑	❑	❑	❑	❑
12. Vous étendre sur le lit	❑	❑	❑	❑	❑	❑
13. Entrer ou sortir d'une baignoire	❑	❑	❑	❑	❑	❑
14. Vous asseoir	❑	❑	❑	❑	❑	❑
15. Vous asseoir et vous relever des toilettes	❑	❑	❑	❑	❑	❑
16. Faire le ménage « à fond » de voter domicile	❑	❑	❑	❑	❑	❑
17. Faire l'entretien quotidien de votre domicile	❑	❑	❑	❑	❑	❑
Somme de chaque colonne	**0**	|__|__| entre 0 et 17	|__|__| entre 0 et 34	|__|__| entre 0 et 51	|__|__| entre 0 et 68	|__|__| entre 0 et 20
Total	|__|__| entre 0 et 68					|__|__| entre 0 et 20
Score	= (Total/17)*25<br>|__|__|__| (0-100)					= (Total/5)*25<br>|__|__|__| (0-100)

Annex 3. Eleven-point numeric rating scale for patient's global assessment of improvement

Échelle numérique pour l'auto-évaluation de l'amélioration des symptômes

Cette échelle numérique a pour but d'évaluer **l'amélioration au niveau du genou** depuis votre dernière évaluation. Pour cela, il suffit de cocher une case sur l'échelle selon l'amélioration ressentie.

- Évaluation de l'amélioration **au niveau du genou** depuis votre dernière évaluation :

Depuis votre dernière évaluation, comment jugez-vous l'amélioration au niveau du genou ?

Aucune amélioration **Amélioration maximale**

☐	☐	☐	☐	☐	☐	☐	☐	☐	☐	☐
**0**	**10**	**20**	**30**	**40**	**50**	**60**	**70**	**80**	**90**	**100**

Annex 4. OARSI-OMERACT set of responder criteria [35]

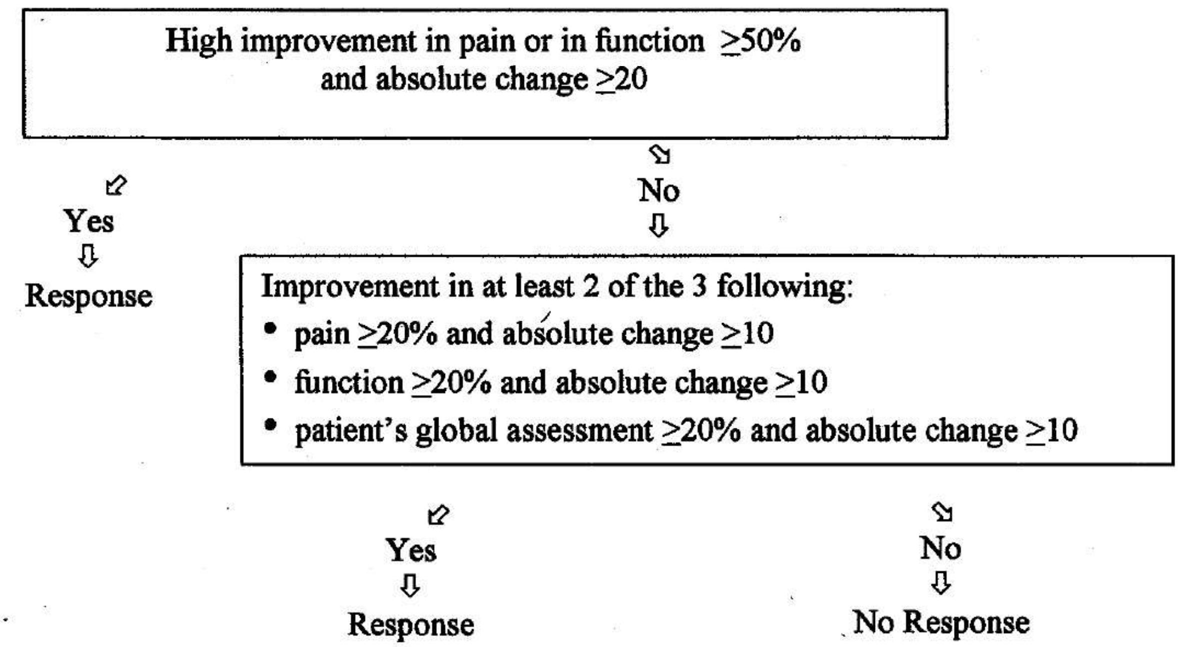

Annex 5. SAE Form

Direction de l'Organisation Médicale et des relations avec les Universités (DOMU)<br><br>Département de la Recherche Clinique et du Développement (DRCD)	**Formulaire de notification d'un Evènement Indésirable Grave (EIG) survenant au cours d'une Recherche Biomédicale ne portant pas sur un produit de santé**	**PARTIE RESERVEE AU PROMOTEUR**<br><br>**REFERENCE VIGILANCE :**<br><br>Référence GED : REC-DTYP-0271

Dès la prise de connaissance de l'EIG par l'investigateur, ce formulaire doit être dûment complété (3 pages), signé et retourné sans délai au secteur Vigilance du DRCD-Siège par télécopie au +33 (0)1 44 84 17 99

Notification initiale ☐ **Suivi d'EIG** ☐ **N° du suivi** |__|__|

**1. Identification de la recherche**	
**Acronyme : ARTHROL**	**Date de notification :** |__|__| |__|__| |_2_|_0_|__|__| (jj mm aaaa)
**Code de la Recherche : P150938**<br>**Risque : A**	**Date de prise de connaissance de l'EIG par l'investigateur :** |__|__| |__|__| |_2_|_0_|__|__| (jj mm aaaa)

Titre complet de la Recherche Biomédicale :

EVOLUTION OF PAIN AT THREE MONTHS BY ORAL RESVERATROL IN PRIMARY KNEE OSTEOARTHRITIS : A MULTICENTER, DOUBLE-BLIND, RANDOMIZED, PLACEBO-CONTROLLED TRIAL

**2. Identification du centre investigateur**	
Nom de l'établissement : ....<br>Ville et code postal : ....<br>Service :	Investigateur (nom/prénom) : ....<br>Tél : .... Fax : ....

**3. Identification et antécédents de la personne se prêtant à la recherche**		
Référence de la personne : |__|__|__| - |__|__|__|__|__| - |__| - |__| (n° centre - n° ordre de sélection - initiale nom - initiale prénom)		**Antécédents médicaux-chirurgicaux/familiaux pertinents pour l'évaluation du cas (joindre un CRH le cas échéant) :** ....
Sexe : ☐ M ☐ F	Date de naissance : |__|__| |__|__| |__|__|__|__| (jj mm aaaa)	
Poids : |__|__|__| kg<br>Taille : |__|__|__| cm	Age : |__|__|__|ans	
Date de signature du consentement : |__|__| |__|__| |_2_|_0_|__|__| (jj mm aaaa)		
Date de randomisation : |__|__| |__|__| |_2_|_0_|__|__| (jj mm aaaa)		N° traitement ou N° randomisation :

**4. Procédures et actes ajoutés par la recherche** *(ex. : biopsies, IRM … Barrer l'encadré si procédures et actes non réalisés)*	Date de réalisation *(jj/mm/aaaa)*	Chronologie	
		Avant la survenue de l'EIG	Après la survenue de l'EIG
RESVERATROL ou Placebo	|__|__| |__|__| |_2_|_0_|__|__|	☐	☐
	|__|__| |__|__| |_2_|_0_|__|__|	☐	☐
....	|__|__| |__|__| |_2_|_0_|__|__|	☐	☐
....	|__|__| |__|__| |_2_|_0_|__|__|	☐	☐

5. Médicament(s) concomitant(s) au moment de l'EIG, à l'exclusion de ceux utilisés pour traiter l'événement indésirable

(compléter le tableau ci-après et si nécessaire l'annexe relative aux médicaments concomitants ou barrer l'encadré si non applicable)

➪ **Annexe jointe au présent formulaire :** ☐ Oui ☐ Non

Acronyme : Erreur ! Source du renvoi introuvable.**ARTHROL**

Référence de la personne se prêtant à la recherche : |__|__|__| - |__|__|__|__|__| - |__| - |__|
n° centre - n° ordre de sélection - initiale nom - initiale prénom

PARTIE RESERVEE AU PROMOTEUR

REFERENCE VIGILANCE :

Référence GED : **Erreur ! Source du renvoi introuvable.**

Nom commercial (de préférence) ou Dénomination Commune Internationale	Voie[1]	Posologie (préciser l'unité ex : mg/j)	Dates d'administration *(du jj/mm/aa au jj/mm/aa)*	En cours[2]	Indication	***Action prise***<br>*0 : poursuite sans modification de la posologie*<br>*1 : arrêt*<br>*2 : diminution de la posologie*<br>*3 : augmentation de la posologie*<br>*4 : ne sais pas*	***Causalité de l'EIG***<br>*0 : non lié au médicament*<br>*1 : lié au médicament*<br>*2 : ne sais pas*
			du |__|__| |__|__| |__|__| au |__|__| |__|__| |__|__|	☐			
			du |__|__| |__|__| |__|__| au |__|__| |__|__| |__|__|	☐			

(1) Voie d'administration : VO=voie orale ; IM=Intramusculaire ; IV=intraveineuse ; SC=sous-cutanée ou autre (à préciser) (2) En cours au moment de la survenue de l'EIG

6. Evènement indésirable grave [EIG]

Diagnostic : ☐ Définitif ☐ Provisoire

....

....

Date de survenue des premiers symptômes : |__|__| |__|__| |_2_|_0_|__|__|

Préciser lesquels ....

....

Organe(s) concerné(s) :

....

....

Date d'apparition de l'EIG :

|__|__| |__|__| |_2_|_0_|__|__|
jj mm aaaa

Heure de survenue : |__||__| hh |__||__| min

☐ *donnée manquante*

Délai entre la date de procédure/acte ajouté par la recherche et la date de survenue de l'EIG :

|__||__| / |__||__| |__||__|
jj hh min

Critères de gravité :

☐ Nécessite ou prolonge l'hospitalisation :

du |__|__| |__|__| |_2_|_0_|__|__|

au |__|__| |__|__| |_2_|_0_|__|__| ☐ *en cours*

☐ Décès

☐ Mise en jeu du pronostic vital

☐ Incapacité ou handicap important ou durable

☐ Anomalie ou malformation congénitale

☐ Autre(s) critère(s) médicalement significatif(s), préciser :

....

....

Degré de sévérité :

☐ Léger ☐ Modéré ☐ Sévère

L'évènement a-t-il conduit à une interruption de la procédure/acte ajouté par la recherche ?

☐ Non ☐ Oui Date : |__|__| |__|__| |_2_|_0_|__|__| ☐ Non applicable

L'arrêt de la procédure/acte ajouté par la recherche a été :

○ Provisoire ○ Définitif

Le cas échéant, date de reprise de la procédure/acte ajouté par la recherche :

|__|__| |__|__| |_2_|_0_|__|__| ☐ Non applicable

Récidive de l'EIG après reprise de la procédure/acte ajouté par la recherche :

○ Non ○ Oui Date : |__|__| |__|__| |_2_|_0_|__|__| ☐ Non applicable

Des mesures symptomatiques ont-elles été prises ?

☐ Non ☐ Oui Date : |__|__| |__|__| |_2_|_0_|__|__|

Préciser : ....

....

L'évènement a-t-il conduit à une levée d'insu ?

☐ Non ☐ Oui Date : |__|__| |__|__| |_2_|_0_|__|__| Résultat : ……………………………

Evolution de l'événement

☐ **Décès**

○ sans relation avec l'EIG

○ en relation avec l'EIG

Date : |__|__| |__|__| |_2_|_0_|__|__|
jj mm aaaa

☐ **Sujet non encore rétabli**, préciser :

○ Etat stable

○ Aggravation

○ Amélioration

☐ **Résolu :**

○ sans séquelles

○ avec séquelles, préciser lesquelles :

....

....

Date : |__|__| |__|__| |_2_|_0_|__|__|
jj mm aaaa

|__|__| |__|__|
hh min

☐ **Evolution inconnue**

PARTIE RESERVEE AU PROMOTEUR

REFERENCE VIGILANCE :

Référence GED : **Erreur ! Source du renvoi introuvable. Erreur ! Source du renvoi introuvable.**

7. Autre(s) étiologie(s) envisagée(s)

☐ Non ☐ Oui Si oui, préciser : ……………………………………………………………………………………
……
……

8. Examen(s) complémentaire(s) réalisé(s)

☐ Non ☐ Oui Si oui, préciser date, nature et résultats : [joindre les bilans anonymisés] ……………………
……
……
……

9. Selon l'investigateur, l'événement indésirable grave est (plusieurs cases possibles)

Lié à la recherche biomédicale :

☐ Oui : ○ **à la (aux) procédure(s)/acte(s) de la recherche biomédicale : resveratrol ou placebo**
☐ Relation certaine ☐ Relation probable ☐ Relation possible ☐ Relation improbable (non exclu)

☐ Non : ○ **à la progression de la maladie faisant l'objet de la recherche :** arthrose du genou
○ **à un (ou plusieurs) médicament(s) concomitant(s) administré(s), le(s)quel(s) :** ……………………
○ **à une maladie intercurrente, laquelle :** ……………………………………………………
○ **autre, préciser :** ……………………………………………………………………

Notificateur	Investigateur	Tampon du service :
Nom et fonction :	Nom :	
Signature :	Signature :	

Annex 6. Patient Card

CARTE PATIENT

Merci de garder cette carte en permanence avec vous

Nom :…………………………………… Prénom : …………………………..

Code d'identification : Code d'identification Patient

___ ___ ___ / ___ ___ ___ ___ / ___ ___

N° centre / N° d'inclusion/ Initiales Nom Prénom

Je participe à la recherche *: ARTHROL*
Evaluation des effets du Resveratrol per os dans l'arthrose du genou

dont le promoteur est l'Assistance Publique – Hôpitaux de Paris

Je reçois le traitement suivant : ***Resvératrol* ou *Placebo***

A la dose de : 80 mg (***2 gélules 2 fois par jour) pendant 1 semaine***

Puis 40 mg (1 gélule 2 fois par jour) pendant 6 mois

Date de début de traitement : __ __ /__ __ /__ __ __ __

N° de traitement reçu = __ __ __ __

CARTE PATIENT

Je suis suivi(e) par le Dr…………………………………………

A l'Hôpital ……………………………………………..…………..

☎……………………………………………………………..

En cas de nécessité de connaître votre traitement en urgence, votre médecin peut contacter le Centre Anti-Poison de l'hôpital Fernand Widal, à Paris

☎: 01 40 05 48 48

Annex 7. Communication

Arthrose du genou : l'hôpital Cochin recrute des patients pour une étude clinique

Vous avez une arthrose du genou douloureuse, confirmée par une radiographie, et êtes âgé de plus de 40 ans ? Le Professeur François Rannou, adjoint au Chef du Service de Rééducation et Réadaptation de l'Appareil Locomoteur et des Pathologies du Rachis, lance début 2017 une étude clinique sur l'arthrose du genou. 164 patients doivent être inclus dans cette recherche. Dans le cadre de cette étude, les patients bénéficieront d'une consultation médicale et recevront pendant 6 mois un complément nutritionnel ou le placebo. Si vous souhaitez y participer, contactez-nous au : *adresse générique aphp*

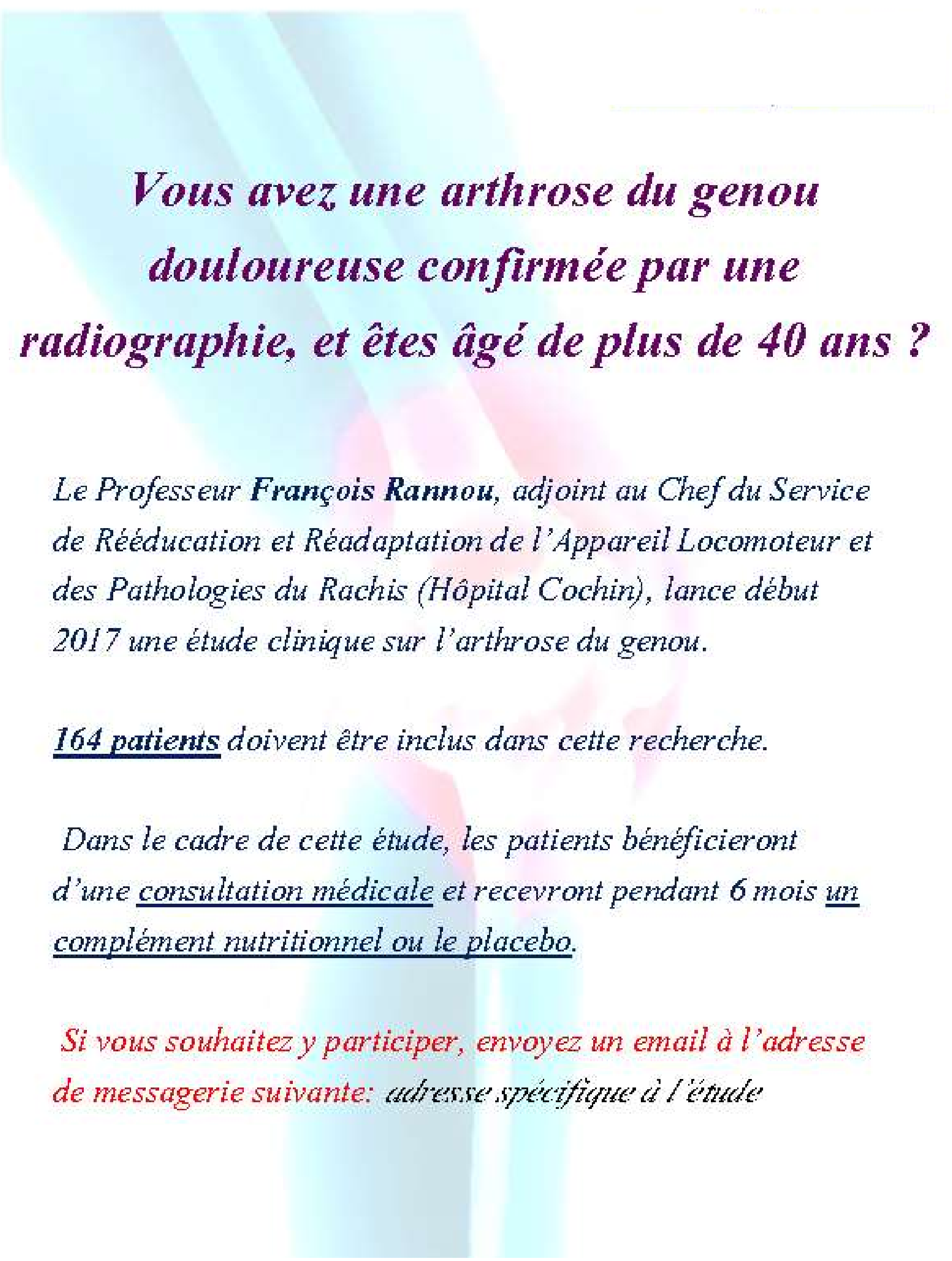

Annexe 4 : affiche destinée au recrutement patient

EVOLUTION OF PAIN AT THREE MONTHS BY ORAL RESVERATROL IN PRIMARY KNEE OSTEOARTHRITIS : A MULTICENTER, DOUBLE-BLIND, RANDOMIZED, PLACEBO-CONTROLLED TRIAL

ACRONYM: ARTHROL

Réf projet: **P150938** / ANSM: N° **ID RCB : 2016-A01310-51**

Version N°7.0 of 23/03/2021

Coordinating Investigator / Scientific Director

Prof. François RANNOU, M.D., Ph.D.
Service de Rééducation et de Réadaptation de l'Appareil Locomoteur et des Pathologies du Rachis
Hôpitaux Universitaires Paris Centre - Groupe Hospitalier Cochin
Assistance Publique – Hôpitaux de Paris
27, Rue du Faubourg Saint-Jacques, 75014 Paris, FRANCE
Tel +33 1 58 41 25 35
Fax +33 1 58 41 25 45
francois.rannou@aphp.fr

Dr. Christelle NGUYEN, M.D., Ph.D.
Service de Rééducation et de Réadaptation de l'Appareil Locomoteur et des Pathologies du Rachis
Hôpitaux Universitaires Paris Centre - Groupe Hospitalier Cochin
Assistance Publique – Hôpitaux de Paris
27, Rue du Faubourg Saint-Jacques, 75014 Paris, FRANCE
Tel +33 1 58 41 29 45
Fax +33 1 58 41 28 35
christelle.nguyen2@aphp.fr

Methodologist

Prof. Isabelle Boutron
Centre d'Épidémiologie Clinique
Centre de Recherche Épidémiologie et Statistique Sorbonne Paris Cité (CRESS-UMR1153)

INSERM / Université Paris Descartes
Hôpital Hôtel-Dieu, 1 Place du Parvis Notre-Dame, 75004 Paris, FRANCE
Tel :+33 1 42 34 78 33
isabelle.boutron@.aphp.fr

Sponsor

Assistance Publique - Hôpitaux de Paris (AP-HP)
By delegation: Clinical Research and Innovation Department (DRCI)
Hôpital Saint-Louis
1, Avenue Claude Vellefaux, 75010 Paris, France
Chef de Projet : Sylvie PRIEUR
Tel + 33 1 44 84 17 92
Fax +33 1 44 84 17 01
sylvie.prieur@aphp.fr

Entity responsible for monitoring research

Unité de Recherche Clinique (URC) Paris Descartes Necker Cochin
GH Cochin – Hôpital Tarnier
89 rue d'Assas
75006 PARIS
Chef de Projet: Laetitia PEAUDECERF
Tel +33 1 58 41 12 13
Fax + 33 1 58 41 11 83
laetitia.peaudecerf@aphp.fr

Entity responsible for investigational products circuit :

Département Essais cliniques de l'AGEPS
Pharmacist: Dr Blandine Lehmann
Chef de projets : Kevin CARDET
Tel: +33 1 46 69 14 02

CONTENTS

1 SUMMARY 5

2 SCIENTIFIC JUSTIFICATION FOR THE RESEARCH 8

2.1 HYPOTHESIS FOR THE RESEARCH 8
2.2 DESCRIPTION OF KNOWLEDGE RELATING TO THE HYPOTHESIS FOR THE RESEARCH 9
2.3 JUSTIFICATION OF THE RESEARCH 9
2.4 PRIMARY OBJECTIVE 11
2.5 SECONDARY OBJECTIVES 11

3 PLAN FOR THE RESEARCH 12

3.1 CONCISE DESCRIPTION OF THE PRIMARY AND SECONDARY ASSESSMENT CRITERIA 12
3.2 DESCRIPTION OF RESEARCH METHODOLOGY 13

4 PROCEDURE FOR THE RESEARCH 15

4.1 SELECTION 15
4.2 INCLUSION AND RANDOMIZATION VISIT 15
4.3 3-MONTH ASSESSMENT 17
4.4 6-MONTH ASSESSMENT AND END OF RESEARCH 17
4.5 EXPECTED LENGTH OF PARTICIPATION AND DESCRIPTION OF THE CHRONOLOGY AND DURATION OF THE RESEARCH. 18
4.6 TABLE OR DIAGRAM SUMMARISING THE CHRONOLOGY OF THE RESEARCH 18
4.7 DISTINCTION BETWEEN CARE AND RESEARCH 19
4.8 TERMINATION RULES 20

5 ELIGIBILITY CRITERIA 21

5.1 INCLUSION CRITERIA 21
5.2 EXCLUSION CRITERIA 21
5.3 RECRUITMENT METHODS 22

6 EXPERIMENTAL PRODUCTS ADMINISTERED TO RESEARCH PARTICIPANTS 22

6.1 DESCRIPTION OF THE EXPERIMENTAL PRODUCTS 22
6.2 AUTHORISED AND PROHIBITED TREATMENTS (MEDICINAL, NON MEDICINAL, SURGICAL), INCLUDING RESCUE MEDICATIONS 23
6.3 METHODS FOR MONITORING COMPLIANCE WITH THE EXPERIMENTAL PRODUCTS 23

7 ASSESSMENT OF EFFICACY 23

8 STATISTICAL ASPECTS 24

8.1 CALCULATION OF SAMPLE SIZE 24
8.2 DESCRIPTION OF STATISTICAL METHODS TO BE USED 24
8.3 STATISTICAL ANALYSIS PLAN 25

9 SAFETY ASSESSMENT - RISKS AND RESTRICTIONS ADDED BY THE RESEARCH .. 25

9.1 DEFINITIONS 25
9.2 THE INVESTIGATOR'S ROLES 26
9.3 SPECIFIC FEATURES OF THE PROTOCOL 28
9.4 PROCEDURES AND DEADLINES FOR NOTIFYING THE SPONSOR 29
9.5 PERIOD FOR NOTIFYING THE SPONSOR 30
9.6 THE SPONSOR'S ROLES 30
9.7 DATA SAFETY MONITORING BOARD (DSMB) 32

10 DATA MANAGEMENT 32

10.1 DATA COLLECTION METHODS 32
10.2 RIGHT TO ACCESS SOURCE DATA AND DOCUMENTS 33
10.3 DATA PROCESSING AND STORAGE OF DOCUMENTS AND DATA 33
10.4 OWNERSHIP OF THE DATA 34

11 QUALITY CONTROL AND ASSURANCE 34
11.1 GENERAL ORGANISATION 34
11.2 LEVEL OF CENTRE MONITORING 35
11.3 QUALITY CONTROL 35
11.4 CASE REPORT FORM (CRF) 35
11.5 MANAGEMENT OF NON-COMPLIANCES 36
11.6 AUDITS/INSPECTIONS 36
11.7 PRIMARY INVESTIGATOR'S COMMITMENT TO ASSUME RESPONSIBILITY 36
12 ETHICAL AND LEGAL CONSIDERATIONS 37
12.1 METHODS FOR OBTAINING INFORMATION AND CONSENT FROM RESEARCH PARTICIPANTS 37
12.2 SUBJECT PROHIBITED FROM PARTICIPATING IN ANOTHER RESEARCH OR AN EXCLUSION PERIOD ANTICIPATED AFTER THE RESEARCH, IF APPLICABLE 37
12.3 COMPENSATION FOR SUBJECTS 37
12.4 LEGAL OBLIGATIONS 38
12.5 MODIFICATIONS TO THE RESEARCH 38
12.6 FINAL RESEARCH REPORT 39
13 FUNDING AND INSURANCE 39
13.1 FUNDING SOURCE 39
13.2 INSURANCE 39
14 PUBLICATION RULES 39
14.1 MENTION OF THE AP-HP MANAGER (DRCI) IN THE ACKNOWLEDGEMENTS OF THE TEXT 40
14.2 MENTION OF THE FINANCIER IN THE ACKNOWLEDGEMENTS OF THE TEXT 40
15 BIBLIOGRAPHY 40
16 LIST OF ADDENDA 43
16.1 LIST OF INVESTIGATORS 43
16.2 ANNEXES 45

1 SUMMARY

Title	Evolution of pain at three months by oral resveratrol in primary knee osteoarthritis: a multicenter, double-blind, randomized, placebo-controlled trial
Acronym	ARTHROL
Coordinating Investigator<br><br><br><br><br><br><br><br><br><br>Scientific Director	Prof. François RANNOU, M.D., Ph.D.<br>Service de Rééducation et de Réadaptation de l'Appareil Locomoteur et des Pathologies du Rachis<br>Hôpitaux Universitaires Paris Centre - Groupe Hospitalier Cochin<br>Assistance Publique – Hôpitaux de Paris<br>27, Rue du Faubourg Saint-Jacques, 75014 Paris, FRANCE<br>Tel +33 1 58 41 25 35<br>Fax +33 1 58 41 25 45<br>francois.rannou@aphp.fr<br><br>Dr. Christelle NGUYEN, M.D., Ph.D.<br>Service de Rééducation et de Réadaptation de l'Appareil Locomoteur et des Pathologies du Rachis<br>Hôpitaux Universitaires Paris Centre, Groupe Hospitalier Cochin<br>Assistance Publique – Hôpitaux de Paris<br>27, Rue du Faubourg Saint-Jacques, 75014 Paris, FRANCE<br>Tel +33 1 58 41 29 45<br>Fax +33 1 58 41 28 35<br>christelle.nguyen2@aphp.fr
Sponsor	Assistance Publique - Hôpitaux de Paris
Scientific justification	OA is the first cause of handicap in individuals over 40 years-old in France. OA physiopathology is driven by local joint inflammation responsible for pain and joint destruction. Experimental studies have shown that resveratrol could modulate pain and inflammation. We hypothesize that resveratrol, in a new formulation developed by the coordinating investigator and his colleagues (INSERM U1124), improving its bioavailability, will decrease pain in patients presenting with primary knee OA.
Primary objective and assessment criterion	The primary objective of the study is to assess the efficacy on mean knee pain in the previous 48 hrs of oral resveratrol compared to placebo in patients with knee OA at 3 months.<br>The assessment criterion is the mean change from baseline in mean knee pain in the previous 48 hours on a self-administered 11-point pain numeric rating scale (NRS, 0 no pain - 100 maximal pain) at 3 months
Secondary objectives and assessment criteria	The secondary objectives of the study are to assess the efficacy of oral resveratrol compared to placebo in patients with knee OA mean knee pain in the previous 48 hrs at 6 months, and function, patient's global assessment, response to treatment and medication (intra-articular injections of corticosteroids or hyaluronic acid, analgesics and non-steroidal anti-inflammatory drugs [NSAIDs]) sparing effect at 3 and 6 months. Assessment criteria are:

	• the mean change from baseline in mean knee pain in the previous 48 hrs on a self-administered 11-point pain NRS at 6 months<br>• the mean change from baseline in the function subscore of the self-administered Western Ontario and McMaster Universities Arthritis Index (WOMAC) questionnaire at 3 and 6 monthsthe mean change from baseline in patient's global assessment at 3 and 6 months on a self-administered 11-point global assessment NRS (0 worst possible - 100 best possible)<br>• the percentage of Osteoarthritis Research Society International (OARSI) - Outcome Measures in Rheumatology (OMERACT) responders at 3 and 6 months<br>• self-reported number of intra-articular injections of corticosteroids or hyaluronic acid since last contact at 3 and 6 months<br>Self-reported consumption of analgesics (non-opioid, weak and strong opioids) since last contact using a self-administered 4-class scale (never; several times a month; several times a week; daily) at 3 and 6 months<br>• self-reported consumption of NSAIDs since last contact using a self-administered 4-class scale at 3 and 6 months
Experimental design	Multicenter, double-blind, randomized, placebo-controlled trial
Population involved	Patients presenting with symptomatic primary knee OA, fulfilling 1986 American College of Rheumatology (ACR) classification criteria
• Inclusion criteria	• Age ≥ 40 years-old<br>• Knee OA fulfilling 1986 ACR criteria<br>• Pain on an 11-point NRS ≥ 40/100<br>• Symptom duration ≥ 1 month<br>• 4 > Kellgren and Lawrence X-Ray score ≥ 1<br>• Written consent obtained<br>• Health insurance cover<br>• Patients excluded for temporary reasons can be rescreened.
• Non-inclusion criteria	• History of symptomatic crystal or inflammatory arthritis<br>• Knee surgery ≤ 1 year<br>• Knee trauma ≤ 2 months<br>• Knee intra-articular injections ≤ 2 months<br>• Current use of intramuscular, intravenous or oral corticosteroids<br>• Current use of anticoagulants<br>• Uncontrolled diseases that may require intramuscular, intravenous or oral corticosteroids<br>• Neurologic disorders involving the lower limbs<br>• Inability to speak, write or read French language<br>• Participation to another biomedical research<br>• Contraindication to resveratrol or hypersensitivity to any of its constituents
Investigational product	Resveratrol is a dietary supplement, not a drug. It will be administered orally 2 caplets twice a day for one week then one capsule twice a day for a total duration of 6 months. Resveratrol will be supplied by the industrial partner. The caplets of resveratrol have already been distributed on the

	French market for several years and the caplets used in this study will be exactly the same as those already available on the French market.
Control group	Placebo of resveratrol will be supplied by the industrial partner. It will present with similar conditioning and taste, and will be administered orally 2 caplets twice a day for one week then one capsule twice a day for a total duration of 6 months. The placebo use in this study will be exactly the same as the one used in a previous PHRC already accepted in neurology (Assistance Publique-Hôpitaux de Paris).
Other procedures added by the research	Not applicable
Risks added by the research	Risk A
Practical procedure	• Recruitment of eligible patients by advertisement and among in- and outpatients of the investigating centers<br>• Day 0: inclusion visit, informed consent, inclusion and randomization<br>• Month 3: follow-up visit<br>• Month 6: follow-up visit and end of the research
Number of subjects chosen	164
Number of centres	3 tertiary care centers located in France<br>• Cochin Hospital, Paris<br>• Saint-Antoine Hospital, Paris<br>• Clermont-Ferrand Hospital
Research period	•
Number of inclusions expected per centre and per month	1.1 patients per centre and per month
Statistical analysis	Analysis will be performed according to the intention-to-treat principle by which each participant will be analyzed in his randomization arm, following a prespecified statistical analytic plan
Funding source	Assistance Publique-Hôpitaux de Paris - Programme Hospitalier de Recherche Clinique en 2015
Data Safety Monitoring Board anticipated	No

2 SCIENTIFIC JUSTIFICATION FOR THE RESEARCH

2.1 Hypothesis for the research

In the Global Burden of Disease Study 2013 designed to assess disability-adjusted life-years (DALYs), which is an indicator of health loss, musculoskeletal disorders were identified among the 5 main contributors to DALYs [1]. Knee osteoarthritis (OA) is one of the most disabling joint disorder in Western countries [2]. OA is the first cause of disability in patients over 40 years-old in France [3]. OA physiopathology is driven by local joint inflammation responsible for pain and joint destruction. There is no efficient structural treatment for OA, but only symptomatic treatments, mainly acetaminophen and NSAIDs, designed to alleviate painful symptoms. Unfortunately, acetaminophen is not very effective with an effect size of 0.10, and recent data highlighted its potential cardiovascular adverse effects [4, 5]. NSAIDs are efficient on pain and inflammation, but their serious cardiovascular and digestive side effects do not support a prescription for long duration. Therefore, an optimized treatment in OA should be efficient on both pain and inflammation, as well as innocuous. Resveratrol is an interesting molecule because of its anti-inflammatory properties [6]. Resveratrol is currently used as a dietary supplement. No serious toxicity has been described in humans so far.

A French PHRC is currently ongoing to address resveratrol positive effects on metabolic syndrome and inflammation: AOR12; METARES study, Prof. Jean-Louis Beaudeux, Pitié-Salpêtrière Hospital, Pierre & Marie Curie University. Resveratrol has also been evaluated in aging, cancer, neurodegenerative diseases, menopausal conditions, and cardiovascular and liver diseases [6]. However, the doses used in these trials were highly variable, and were not adjusted regarding the low bioavailability of current oral formulations.

Drs JF Savouret (INSERM U1124) and Éric Serée (scientific advisor for our project) have recently designed and patented a new formulation of resveratrol, in collaboration with our industrial partner (patent n° WO 2012/007252, Laurent Pechère, Yvery Laboratory, Marseille, FRANCE). This new formulation has allowed for the first time a significant increase of resveratrol bioavailability by oral administration in humans, in contrast with former dry powder formulations [7]. The plasmatic peak (30 min. after ingestion) was 10-fold increased, and blood concentration remained at significant levels during several hours. This new resveratrol formulation may allow for a trial with low resveratrol doses (40 mg/day), yet yielding a significant blood level and half-life of resveratrol. In other word, with this new formulation of resveratrol, a putative effect becomes possible via oral administration. To our knowledge, this formulation is unique and no other formulation is able to reach such a high bioavailability.

We hypothesized that the use of resveratrol in this new formulation would significantly decrease mean OA knee pain in the previous 48 hours at 3 months, with minimal adverse effects.

2.2 Description of knowledge relating to the hypothesis for the research

Mediterranean societies have developed the 'Mediterranean diet' under the influence of Greek medicine. This diet consists in a moderate intake of animal meat and fat in combination with high consumption of vegetables, fruits and olive oil. Natural antioxidants, fibers, B vitamins and unsaturated fatty acids are also present, with red wine being the main alcoholic beverage, consumed with meals on a daily basis [8]. The beneficial effect of this diet upon coronary heart disease prevention has been a matter of debate, recently refueled by the controversy over the 'French Paradox'. This concept arose from studies showing the low rate of coronary heart disease in wine-drinking French population compared to other Western populations, despite the presence of elevated risk factors including high animal fat intake, low exercise level, and heavy smoking [9]. This controversy started on epidemiological grounds, further fueled by negative experimental data [10], and eventually lead to the hypothesis that red wine may contain cardioprotective compounds like antioxidant polyphenols.

Red wine contains several polyphenols including phenolic acids, hydroxystilbenes and flavonoids. Trans-resveratrol (3,5,4'-trihydroxystilbene) is the parent compound of a family of hydroxystilbenes existing in cis- and trans- configurations in a variety of spermatophyte plants such as grapevine, peanuts, pine, or Chinese knotweed [10]. Because of its effects on lipids and arachidonic acid metabolisms, and its antioxidant activity, resveratrol has been considered as the major cardioprotective component of red wine. In grapes, resveratrol is produced as an antifungal phytoalexin in response to infection by *Bothrytis Cinerea*. Resveratrol is present in red wine at concentrations ranging from 0.1 to 14.5 mg/L [11]. Several Asian pharmacopoeas describe Chinese knotweed (*Polygonum Cuspidatum*) powder as an anti-inflammatory drug, used in various conditions such as pain, fever, dermatitis, atherosclerosis, hyperlipemia and cancers [12]. **Experimental and clinical data suggest that resveratrol anti-inflammatory and analgesic effects may be of clinical interest as a complementary to conventional treatment in joint diseases. Hypotheses about resveratrol mechanisms of action suggest a modulation of endocrine disruption, apoptosis and oxidative stress.**

2.3 Justification of the research

Even though the research on the effect of dietary polyphenols on human health has developed considerably and controversially in the past decade in such broad fields as cancer, neurodegenerative and cardiovascular diseases, type 2 diabetes mellitus and other metabolic disorders [13], no large clinical data are available to date regarding the efficacy of resveratrol in joint disorders, especially in OA. This lack of trials in joint disorders might be related to controversies associated with the relatively poor results from resveratrol supplementation in other human conditions, particularly with

regards to the financial investments involved [14, 15]. However, a larger body of experimental evidence suggests that resveratrol administered with proper timing and sufficient biodisponibility, might be relevant as a complementary to conventional treatment for joint disorder-related symptoms in humans, in addition to conventional treatments.

***In vitro* findings**

Articular chondrocytes are resistant to resveratrol stimulation at micromolar doses (0.1 to 10 µM), whatever the molecular pathway considered. Several hypotheses can be brought up. Chondrocytes contain high levels of ceruleoplasmin, a copper-containing protein with a strong laccase activity [16]. Laccases are able to disrupt and inactivate resveratrol propylene chain. Chondrocytes also harbour a particular AhR that does not have basal activity and is only able to translocate to the cell *nucleus* upon interleukin (IL)-1β activation (personal data). This apparent insensitivity of chondrocytes to resveratrol *in vitro* due to a lack of AhR basal activity is in contrast with our preliminary clinical observations, showing the potent analgesic activity of resveratrol in patients suffering from a variety of inflammatory joint diseases (personal data). Furthermore, there is growing evidence of resveratrol effects on chondrocytes and synoviocytes.

Resveratrol inhibitory effects on IL-1β and kinases modulation of matrix metalloproteinases (MMP)-1, -3, and -13 expressions in chondrocytes (endochondral cartilage and intervertebral disc) have been reported by several groups [17-21]. In some of these studies, resveratrol doses ranged from 50 to 100 µM. Therefore, an indirect estrogenic effect *via* ER-α activation cannot be excluded [22-25]. Other groups have recently opened a new avenue in cartilage biology research by showing that synoviocytes, more than chondrocytes, might be the real target of resveratrol anti-inflammatory activities. Resveratrol inhibits IL-1β, MMP-3 and phosphorylated Akt expression, either basal or Tumour Necrosis Factor (TNF) α-induced, in a dose-dependent manner, between 6 and 50 µM [26]. Caspase-8 has also been reported to be a target of resveratrol in synoviocytes at high doses (50 µM) [27]. All the authors concluded that resveratrol might have beneficial effects in preventing and treating rheumatoid arthritis (RA). Finally, resveratrol inhibits cell adhesion between monocytes and endothelial cells [28], a mechanism that might be extended to monocyte interactions with chondrocytes. The authors attributed this effect to tyrosine kinase inhibition, the other major effect of resveratrol with AhR activation, although the latter mechanism is also a potent pathway for ICAM-I expression [29].

Pre-clinical findings

Although *in vitro* data strongly support a potent joint protection effect of resveratrol through modulation of inflammation, chondrolysis and angiogenesis, resveratrol health benefits still await in-depth investigation in animal models. Many reports used questionable modes of administration (wine

or dry powder form) or erroneous targets, as once pointed out about platelet aggregation [10]. Only few papers have specifically investigated effects of resveratrol in OA animal models.

In a rabbit model of OA, by unilateral anterior cruciate ligament transection, intra-articular injections of resveratrol hampered the progression of cartilage destruction and associated prod-degradative soluble factor production [30, 31]. In Wang's study, intra-articular resveratrol was administered daily for 2 weeks at different dose regimen (50, 20, and 10 µmol/kg). In the groups treated with resveratrol, reduced cartilage lesions, apoptosis rate of chondrocytes and level of nitric oxid in the synovial fluid were observed in a dose-dependent fashion [31]. Consistently, Elmali and colleagues showed a reduction in cartilage destruction scores and loss of matrix proteoglycans in animals injected intra-articularly with 10 µmol/kg resveratrol for 2 weeks compared to DMSO injected animals. Scores of synovial inflammation were comparable between the 2 groups [30]. Most recently, in a mouse model of OA, by destabilization of the medial meniscus, weekly intra-articular injection of resveratrol in the knee was associated with decreased cartilage and subchondral bone changes, along with unchanged type 2 collagen expression, and reduction in iNOS and MMP-13 expressions, activation of SIRT-1 and inhibition of Hypoxia Inducible Factor (HIF)-2α [32]. To our knowledge, no study has reported the effects of oral administration of resveratrol in these models, or the influence of genetic inhibition of AhR in AhR -/-.

In summary, in the field of rheumatic disorders, *in vitro* evidence clearly support anti-inflammatory, anti-catabolic, anti-apoptotic and anti-oxidative properties of resveratrol in various articular cell types including chondrocytes and synoviocytes, along with immunomodulation properties on T and B lymphocytes. Consistently, resveratrol administered intra-articularly has shown joint protective effects in pre-clinical models of OA, mainly mediated by decreased production of pro-inflammatory and pro-degradative soluble factors, as well as modulation of cellular and humoral responses.

In order to take the use of resveratrol to the next step of clinical trials in human joint diseases, we believe that new formulations of resveratrol as developed by our industrial partner, that improve its biodisponibility and safety [7], could be interesting in treating painful symptoms related to OA as a complementary treatment to conventional treatment.

2.4 Primary objective

The primary objective of the study is to assess the impact on mean knee pain in the previous 48 hrs of oral resveratrol compared to placebo in patients with knee OA at 3 months.

2.5 Secondary objectives

The secondary objectives of the study are to assess the impact of oral resveratrol compared to placebo in patients with knee OA on mean knee pain in the previous 48 hrs at 6 months, and function, patient's global assessment, response to treatment and medication (intra-articular injections of corticosteroids or hyaluronic acid, analgesics and non-steroidal anti-inflammatory drugs [NSAIDs]) sparing effect at 3 and 6 months.

3 PLAN FOR THE RESEARCH

3.1 Concise description of the primary and secondary assessment criteria

Primary and secondary assessment criteria core set was selected accordingly with OMERACT recommendations [33] and COMET initiative for phase III clinical trials in knee OA, and includes outcomes for pain, physical function, and patient global assessment of improvement.

Primary assessment criterion

The primary assessment criterion of the study is the mean change from baseline in mean knee pain in the previous 48 hrs on a self-administered 11-point pain numeric rating scale (NRS, 0 no pain - 100 maximal pain) at 3 months (Annex 1).

Secondary assessment criteria

Secondary assessment criteria are:

- The mean change from baseline in mean knee pain in the previous 48 hrs on a self-administered 11-point pain NRS at 6 months
- The mean change from baseline in the function subscore of the self-administered Western Ontario and McMaster Universities Arthritis Index (WOMAC) questionnaire at 3 and 6 months using the French version of the questionnaire **(Annex 2).** The WOMAC index is a self-administered, disease specific instrument validated for OA. It consists of 24 items grouped into 3 subscales: pain (5 questions), stiffness (2 questions) and physical function (17 questions) with higher scores indicating greater disease severity [34].
- The mean change from baseline in patient's global assessment at 3 and 6 months on a self-administered 11-point global assessment NRS (0 worst possible - 100 best possible) **(Annex 3).**
- The percentage of Osteoarthritis Research Society International (OARSI) - Outcome Measures in Rheumatology (OMERACT) responders at 3 and 6 months. Response to treatment will be defined as an improvement in pain (assessed by an 11-point pain NRS) or in function (assessed by the WOMAC function subscore) ≥ 50% and absolute change ≥ 20, or improvement in at least 2 of the 3

following: 1/ pain ≥ 20% and absolute change ≥ 10, 2/ function ≥ 20% and absolute change ≥ 10, 3/ patient's global assessment (assessed by an 11-point global assessment NRS) ≥ 20% and absolute change ≥ 10 [35] **(Annex 4).**

- The self-reported number of intra-articular injections of corticosteroids or hyaluronic acid since last contact at 3 and 6 months **(Annex 5)**
- The self-reported consumption of analgesics (non-opioid, weak and strong opioids) since last contact using a self-administered 4-class scale (never; several times a month; several times a week; daily) at 3 and 6 months **(Annex 5)**
- The self-reported consumption NSAIDs since last contact using a self-administered 4-class scale at 3 and 6 months **(Annex 5)**

3.2 Description of research methodology

Experimental plan. The proposed study is a 2-arm, placebo-controlled, double-blind, multicentre, randomized trial. Patients will be assessed at baseline, 3 and 6 months. They will be randomized in one of two groups:

- Intervention group: 2 caplets of resveratrol (40 mg) twice a day for one week then one capsule (20 mg) twice a day administered orally for a total duration of 6 months. Resveratrol will be supplied by the industrial partner. The caplets of resveratrol have already been distributed on the French market for several years and the caplets used in this study will be exactly the same as those already available on the French market.
- Control group: 2 caplets of placebo twice a day for one week then one capsule twice a day administered orally for a total duration of 6 months. The placebo of resveratrol will be supplied by the industrial partner. Placebo will present in same conditioning, with the same taste, and same way of administration.

Duration of participation for each patient will be 6 months. The study will be planned, executed, analyzed and reported according to CONSORT Statement [38].

Number of centres participating. The proposed study is a national multicenter study. Individuals will be prospectively recruited among in- and outpatients who fulfill the inclusion criteria from 3 French Rheumatology and Rehabilitation Medicine departments located in tertiary care centers with high expertise in OA management and through advertising on the internet, in the medias (newspapers, health magazines) and using posters displayed in each investigating centres. In addition, the computerized medical records of each investigating center will be searched from 2015 to 2017, and patients for whom the key words "knee osteoarthritis" were recorded will be invited to participate in the study by phone by the biomedical research technician.

Identification of the subjects. For this research, participants will be identified as follows: *centre n° (3 numerical positions) – selection order n° of the participant in the research (4 numerical positions) – surname initial - first name initial. This reference is unique and will be used for the entire research period. A randomization number will be assigned during randomization*

Randomization. Patients who meet the inclusion criteria and agreed to participate will be randomly assigned to the resveratrol or placebo group at day 0.

Sequence generation. The randomization sequence will be computer-generated by a statistician of the Centre d'Épidémiologie Clinique. The list will be stratified by centres with variable block sizes.

Implementation. The randomization process will be centralized at the coordinating office (*Unité de Recherche Clinique, Cochin Hospital*), which will have no involvement in the enrollment, follow-up, or assessment of participants. Only the independent statistician of the Centre d'Épidémiologie Clinique, the computer programmer at the coordinating office (*Unité de Recherche Clinique, Cochin Hospital*) who will implement the list in the secure electronic case report form (e-CRF), and the YVERY company who will prepare the resveratrol and placebo caplets according to the randomization list will have access to the randomization list. The YVERY company will label the resveratrol and placebo caplets and send them to each centre for the whole research duration. In each centre, the investigator will blindly deliver the medication to patients enrolled in the study according to their randomization number.

Allocation concealment. The sequence will be concealed by use of a computer interface implemented in the e-CRF (CleanWeb).

Blinding methods and provisions put in place to maintain blindness. Patients, care providers, data collectors, outcome assessors and statisticians will be blinded to the allocated group. The industrial partner will supply *resveratrol and placebo caplets* with strictly identical presentations, treatment administration and clinical monitoring of the experimental products *will be the same in the experimental and control groups. Resveratrol and placebo caplets* will be prepared according to the randomization list. Labelling will be anonymized by the YVERY company. They will be delivered at once for the whole research duration.

Procedures for breaking the blind, if applicable. The event of having to break the blind is unlikely with the use of a dietary supplement. The blind can be broken only if the investigator deems it necessary for the safe *management of a specific medical condition* of a subject, and whenever possible

the medical monitoring methodologist and sponsor should be consulted before breaking the blind. If the blind is broken for any reason during the course of the study, the moment on which the blind was broken and all other relevant information will be documented by the investigative site, and other sponsor designees, as appropriate. The reason for breaking the blind will be indicated and justified in the source documentation and in the eCRF. All subjects who are unblinded while on the study will be followed-up from the moment of unblinding, with the reason for unblinding given. If an *an adverse event (*AE) leads to unblinding, the AE should be given as the reason for unblinding and the AE should also be recorded in the eCRF. Any AEs should be followed until resolution.

Unblinding will be requested for any reason considered essential by the investigating doctor by calling upon:

- the DRCI **in a situation other than an emergency** during the work day and during working hours, addressed to the DRCI 's project referent **Tel: +33 1 44 49 59 69**
- the poison centre of Fernand Widal Hospital, in the case of an emergency (see emergency situations requiring unblinding), on weekends, bank holidays, when the DRCI is closed and when unblinding cannot be carried out at the DRCI **Tel: +33 1 40 05 48 48**

4 PROCEDURE FOR THE RESEARCH

4.1 Selection

Patients will be prospectively recruited by the treating physician among in- and out-patients and by advertising on the internet, in the medias (newspapers, health magazines) and using posters **(Annex 6)** displayed in each investigating centres. If a patient is interested in participating to the study, he will have to contact the biomedical research technician by phone or email (contact details provided in the advertisement). The biomedical research technician will check for eligibility criteria by phone, then, if appropriate, will appoint the patient within the month for a face-to-face baseline visit with one of the investigator, who will be a senior specialist in physical rehabilitation medicine and/or in rheumatology. In addition, the computerized medical records of each investigating center will be searched from 2015 to 2017, and patients for whom the key words “knee osteoarthritis” were recorded will be invited to participate in the study by phone by the biomedical research technician.

4.2 Inclusion and randomization visit

- Inclusion and non-inclusion criteria will be checked by the investigator during the face-to-face baseline visit. The patient will be informed and his consent collected. The patient will then be enrolled and randomized. Specific additional clinical examination, laboratory tests or imaging will not be required for the purpose of the study. Information’s regarding demographics,

medical history, previous and current medications will be recorded in the eCRF. Baseline values for assessment criteria will be collected:

- mean knee pain in the previous 48 hrs on a self-administered 11-point pain NRS
- function using the function subscore of the self-administered WOMAC questionnaire
- patient's global assessment on a self-administered 11-point global assessment NRS
- self-reported number of intra-articular injections of corticosteroids or hyaluronic acid in the previous 3 months
- self-reported consumption of analgesics in the previous 3 months using a self-administered 4-class scale
- self-reported consumption of NSAIDs in the previous 3 months using a self-administered 4-class scale
- self-reported consumption of symptomatic slow acting drugs for OA (SYSADOA) in the previous 3 months using a self-administered 4-class scale

Data regarding self-administered questionnaires will be entered in the eCRF by the biomedical research technician.

Overall, inclusion visit will include:

- Verification of inclusion and non-inclusion criteria
- Patient information and informed consent
- Randomization by eCRF
- Recording of the demographics, medical history, previous and current medications in the eCRF
- Collection of baseline values for assessment criteria (pain, function and patient's global assessment)
- Dispensation of experimental products by the investigator to the patient according the number randomization assigned
- Patients will be given a participating card in a clinical trial **(Annex 7)**
- Patients will be asked to keep and return the medication bottles for capsule counts at the 3- and 6-month visits to monitor adherence. However, no specific measures will be taken to enhance adherence

**Subjects whose consent is sought**	**Who informs the subject and collects their consent?**	**When is the subject informed?**	**When is the subject's consent collected?**
Subjects who are participating to the	The investigator and co-investigators participating in	At the preselection contact and at the	At the inclusion visit

biomedical research	the study	inclusion visit	

4.3 3-month assessment

Patients will be assessed by the investigator during a face-to-face visit. 3-month values for assessment criteria will be collected:

- mean knee pain in the previous 48 hrs on a self-administered 11-point pain NRS
- function using the function subscore of the self-administered WOMAC questionnaire
- patient's global assessment on a self-administered 11-point global assessment NRS
- self-reported number of intra-articular injections of corticosteroids or hyaluronic acid since last contact
- self-reported consumption of analgesics since last contact using a self-administered 4-class scale
- self-reported consumption of NSAIDs since last contact using a self-administered 4-class scale
- self-reported consumption of SYSADOA since last contact using a self-administered 4-class scale

Data regarding self-administered questionnaires will be entered in the eCRF by the biomedical research technician.

In addition, the investigator will record AEs since last contact by asking an open-ended question ("Did you have any adverse events since last contact?"), the capsule counts in the medication bottles and non-pharmacological co-interventions using a checklist. In the event that the patient missed his appointment, self-administered questionnaires can be returned by mail or email to the biomedical research technician and AEs and capsule counts collected by phone by the biomedical research technician. In order to reduce the amount of missing data, reminding newsletters sent by mail or email will inform patients of the progression of the study once a month.

4.4 6-month assessment and end of research

Patients will be assessed by the investigator during a face-to-face visit. 6-month values for assessment criteria will be collected:

- mean knee pain in the previous 48 hrs on a self-administered 11-point pain NRS
- function using the function subscore of the self-administered WOMAC questionnaire
- patient's global assessment on a self-administered 11-point global assessment NRS

- self-reported number of intra-articular injections of corticosteroids or hyaluronic acid since last contact
- self-reported consumption of analgesics since last contact using a self-administered 4-class scale
- self-reported consumption of NSAIDs since last contact using a self-administered 4-class scale
- self-reported consumption of SYSADOA since last contact using a self-administered 4-class scale

Data regarding self-administered questionnaires will be entered in the eCRF by the biomedical research technician.

In addition, the investigator will record AEs since last contact by asking an open-ended question ("Did you have any adverse events since last contact?"), the capsule counts in the medication bottles and non-pharmacological co-interventions using a checklist. In the event that the patient missed his appointment, self-administered questionnaires can be returned by mail or email to the biomedical research technician and AEs and capsule counts collected by phone by the biomedical research technician. In order to reduce the amount of missing data, reminding newsletters sent by mail or email will inform patients of the progression of the study once a month.

At the end of the research, patients will be advised to continue their usual medical follow-up with their treating physician. No specific changes will be made. Ending a subject's participation will not affect the normal management of the subject's illness in any way. No exclusion period for another biomedical research will be required.

4.5 Expected length of participation and description of the chronology and duration of the research.

Maximum period between recruitment and inclusion	< 1 month
Inclusion period	48months
The included subjects' length of participation, of which:	
• Treatment period:	6 months
• Follow-up period:	6 months
Total research period:	54months

4.6 Table or diagram summarising the chronology of the research

**Actions**	**Screening**	**Inclusion visit**	**Month 3<br>± 10 days**	**Month 6<br>± 10 days**
Informed consent		X		
Recruitment by phone by the research technician<br>Computerized medical records	X			
Inclusion/exclusion criteria fulfilment		X		
Randomization		X		
Baseline characteristics<br>Medical history<br>Current and previous treatments		X<br>X<br>X		
Dispensation of experimental products		X		
<u>Assessment criteria</u><br>Pain on an 11-point pain NRS<br>Function using the WOMAC function subscale<br>Patient's global assessment on an 11-point global assessment NRS<br>Number of intra-articular injections by self-reporting<br>Analgesics consumption on a 4-class scale<br>NSAIDs consumption on a 4-class scale<br>SYSADOA consumption on a 4-class scale<br>OARSI-OMERACT response (pain, function and patient's global assessment)		 <br>X<br>X<br><br>X<br><br>X<br><br>X<br>X<br>X<br>X	 <br>X<br>X<br><br>X<br><br>X<br><br>X<br>X<br>X<br>X	 <br>X<br>X<br><br>X<br><br>X<br><br>X<br>X<br>X<br>X
<u>Collected data</u><br>Adherence (capsule counts)<br>AEs (open-ended question)<br>Non-pharmacological co-interventions (checklist)			 X<br>X<br>X	 X<br>X<br>X

4.7 Distinction between care and research

Procedures and treatments carried out as part of the research	Procedures and treatments associated with care	Procedures and products added because of the research
**Treatment or Investigational products**	• Analgesics<br>• NSAIDs<br>• Symptomatic slow acting drugs for OA (SYSADOA)<br>• Knee joint injections (corticosteroids or hyaluronic acid)	Resveratrol or Placebo
**Blood samples**	Not applicable	Not applicable
**Imaging, etc.**	Knee X-ray	Not applicable

4.8 Termination rules

Criteria and methods for prematurely terminating the research products. Any subject can withdraw from participating in the research at any time and for any reason. If a subject leaves the research prematurely, data relating to the subject can be used unless an objection was recorded when the subject signed the consent form. The investigator can temporarily or permanently prematurely terminate the research treatment for any reason that affects the subject's safety or which would be in the subject's best interests. If a subject prematurely terminates the research treatment, the subject will still be followed-up, and scheduled follow-up for the research will not be affected. If consent is withdrawn, no data about the subject may be used unless the subject states in writing that he/she does not object. In practice, the subject is excluded from the research. The investigator must: document the reason(s) and collect the assessment criteria when participation in the research ends, if the subject agrees.

Follow-up of the subjects after the premature termination of experimental products. Ending a subject's participation does not affect the normal management of the subject's illness in any way. If there are serious AEs (SAEs), the investigator must notify the sponsor and monitor the subject until the end of the research. If treatment is stopped prematurely due to a SAE, a SAE notification form will be sent by email (eig-vigilance.drc@aphp.fr) to the sponsor. It should be noted that it is possible to send SAE reports to the Safety Department by fax to +33 (0)1 44 84 17 99 only in the event of a failed attempt to send the SAE report by email (in order to avoid duplication).
. The SAE will be monitored until it is resolved.

Methods for replacing subjects, if applicable. Subjects will not be replaced.

Terminating part or all of the research. AP-HP as sponsor or the Competent Authority (ANSM) can prematurely terminate all or part of the research, temporarily or permanently, in the following situations:

- First of all, if suspected unexpected serious adverse reactions (SUSARs) are seen in an arm receiving the resveratrol or if there is a discrepancy in the serious adverse reactions between the 2 arms, and which require a reassessment of the benefit-risk ratio for the research.
- Likewise, if unexpected facts, new information about the product, in light of which the objectives of the research are unlikely to be achieved.
- If it appears that the inclusion objectives are not met.

If the research is terminated prematurely, the decision and justification will be given by the sponsor, AP-HP, to the Competent Authority (ANSM) and to the CPP within 15 days.

5 ELIGIBILITY CRITERIA

5.1 Inclusion criteria

- Age ≥ 40 years-old
- Knee OA fulfilling 1986 ACR criteria
- Pain on an 11-point NRS ≥ 40/100
- Symptom duration ≥ 1 month
- 4 > Kellgren and Lawrence X-Ray score ≥ 1
- Written consent obtained
- Health insurance cover

Patients excluded for temporary reasons can be rescreened.

5.2 Exclusion criteria

- History of symptomatic crystal or inflammatory arthritis
- Knee surgery ≤ 1 year
- Knee trauma ≤ 2 months
- Knee intra-articular injections ≤ 2 months
- Current use of intramuscular, intravenous or oral corticosteroids
- Current use of anticoagulants
- Uncontrolled diseases that may require intramuscular, intravenous or oral corticosteroids
- Neurologic disorders involving the lower limbs

- Inability to speak, write or read French language
- Participation to another biomedical research
- Contraindication to resveratrol or hypersensitivity to any of its constituents

5.3 Recruitment methods

Patients will be prospectively recruited by the treating physician among in- and out-patients and by advertising on the internet, in the medias (newspapers, health magazines) and using posters **(Annex 6)** displayed in each investigating centres. If a patient is interested in participating to the study, he will have to contact the biomedical research technician by phone or email (contact details provided in the advertisement). The biomedical research technician will check for eligibility criteria by phone, then, if appropriate, will appoint the patient within the month for a face-to-face baseline visit with one of the investigator, who will be a senior specialist in physical rehabilitation medicine and/or in rheumatology. In addition, the computerized medical records of each investigating center will be searched from 2015 to 2017, and patients for whom the key words “knee osteoarthritis” were recorded will be invited to participate in the study by phone by the biomedical research technician.

	Number of subjects
Total number of subjects chosen	*164*
Number of centres	*3*
Inclusion period (months)	*48*
Number of subjects/centre	*54.7*
**Number of subjects/centre/month**	***1.1***

6 EXPERIMENTAL PRODUCTS ADMINISTERED TO RESEARCH PARTICIPANTS

6.1 Description of the experimental products

- **Resveratrol** is a dietary supplement, not a drug. Pharmaceutical dosage form used will be 20mg caplets. It will be administered orally, 30 minutes before meals with a glass of water with 2 caplets twice a day, for one week, then one capsule twice a day, for a total duration of 6 months. This dietary supplement is well tolerated and no marked toxicity has been reported [39]. Box containing 7 pillboxes of 60 caplets will be provided. Yvery Company will be in charge of

labelling and blinding operations. Caplets should be stored in its original packaging at room temperature between 15 to 25°C. These caplets have already been available ont the French market for several years.

- **Placebo** of resveratrol will present with similar conditioning and taste. It will be administered orally, 30 minutes before meals with a glass of water with 2 caplets twice a day, for one week, then one capsule twice a day, for a total duration of 6 months. Box containing 7 pillboxes of 60 caplets will be provided. Yvery Company will be in charge of blinding and labelling operations. Caplets should be stored in its original packaging at room temperature between 15 to 25°C. These caplets are already used in a PHRC currently conducted in neurology.

Supply of experimental products:
Yvery will supply patient boxes to the care units.
A stock of patient boxes will be send after the opening visit.
Specific prescription and automatic re supply will be generated by the e CRF

The investigator's brochure indicating the composition and the risks and toxicity of the experimental product will be provided to investigators.

6.2 Authorised and prohibited treatments (medicinal, non medicinal, surgical), including rescue medications

It is not allowed to take any other anticoagulants and resverathrol than the ones provided in the study. All other treatments prescribed are authorised. . Rescue medications (analgesics and NSAIDs) and joint injections (hyaluronic and corticosteroids) will be recorded in the eCRF and assessed.

6.3 Methods for monitoring compliance with the experimental products

Patients will be asked to keep and return the medication bottles for capsule counts at the 3- and 6-month visits to monitor adherence. However, no specific measures will be taken to enhance adherence.

After accountability by the CRA, patient boxes will be sent to Yvery for destruction.

7 ASSESSMENT OF EFFICACY

The primary objective of the study is to assess the impact on mean knee pain in the previous 48 hrs of oral resveratrol compared to placebo in patients with knee OA at 3 months. The assessment criterion is the mean change from baseline in mean knee pain in the previous 48 hrs on a self-administered 11-point pain NRS at 3 months. **(Annex 1)**.

The secondary objectives of the study are to assess the impact of oral resveratrol compared to placebo in patients with knee OA on mean knee pain in the previous 48 hrs at 6 months, and function, patient's global assessment, response to treatment and medication (intra-articular injections of corticosteroids or hyaluronic acid, analgesics and non-steroidal anti-inflammatory drugs [NSAIDs]) sparing effect at 3 and 6 months. Secondary assessment criteria are:

- The mean change from baseline in mean knee pain in the previous 48 hrs on a self-administered 11-point pain NRS at 6 months **(Annex 1)**
- The mean change from baseline in WOMAC function subscore at 3 and 6 months using the French version of the questionnaire **(Annex 2).**
- The mean change from baseline in patient's global assessment at 3 and 6 months on a self-administered 11-point global assessment NRS **(Annex 3).**
- The percentage of OARSI-OMERACT responders at 3 and 6 months **(Annex 4).**
- The self-reported number of intra-articular injections of corticosteroids or hyaluronic acid since last contact at 3 and 6 months **(Annex 5)**
- The self-reported consumption of analgesics (non-opioid, weak and strong opioids) since last contact using a self-administered 4-class scale at 3 and 6 months **(Annex 5)**
- The self-reported consumption of NSAIDs since last contact using a self-administered 4-class scale at 3 and 6 months **(Annex 5)**

8 STATISTICAL ASPECTS

8.1 Calculation of sample size

The sample size is estimated at 164 patients. We have predicted a difference in mean change from baseline of 15 points on the pain NRS between the experimental and the placebo groups, with a standard deviation of 27 points, and a power of 90%, corresponding to 69 patients in each arm. Considering a 15% patient lost to follow-up, we have estimated that we will need to enrol a number of 82 patients for each arm. Fifteen points on pain NRS is considered as the minimal clinically perceived difference in pain for patients with knee OA.

8.2 Description of statistical methods to be used

All analyses will be performed on an intent-to-treat basis, in that all patients will be considered in the analysis and will be analyzed in the group to which they had been assigned. For descriptive analyses, qualitative variables will be reported with absolute and relative frequencies, and quantitative variables with median (interquartile range [IQR]). To compare differences in changes in values

between the 2 treatment groups for quantitative variables, a constrained longitudinal data analysis will be used. In this model, both the baseline and post-baseline values will be modelled as dependent variables (the constrained longitudinal data analysis model assumes that both the baseline and post-baseline measurements are jointly multivariate normally distributed because the baseline value is treated as part of the response vector). The true baseline means will be constrained to be the same for the 2 treatment groups. Hence, this analysis provides an adjustment for the observed baseline difference in estimating the treatment effects. Random effects at patient and centre levels will be added to these models. Results are expressed as differences in mean change from baseline with 95% CI at 3 months and 6 months. The constrained longitudinal data analysis model can include all randomized subjects with a baseline or post-baseline value. Such methods based on maximum likelihood are consistent under the missing-at-random assumption. Qualitative outcomes will be analysed using a mixed logistic regression model with a random effect at centre levels. Data analysis will involve use of SAS 9.4 (SAS Institute, Cary, NC). Blinded statisticians will perform the statistical analyses at an independent center (Centre d'Épidémiologie Clinique, Paris Descartes, Hôpital Hôtel-Dieu).

8.3 Statistical analysis plan

The statistical analysis described below will be further detailed in a dedicated Statistical Analysis Plan (SAP) before any analysis is undertaken.

9 SAFETY ASSESSMENT - RISKS AND RESTRICTIONS ADDED BY THE RESEARCH

9.1 Definitions

According to Article R1123-39 of the French Public Health Code

- **AE**

  Any untoward medical occurrence in a clinical trial subject and which does not necessarily have a causal relationship with the clinical trial or with the experimental product.

- **Adverse reaction**

  Any adverse event caused by the clinical trial.

- **SAE or adverse reaction**

Any **adverse event or reaction** which:

- Results in death,

- Is life threatening,
- Requires hospitalization or prolongation of existing hospitalization,
- Results in persistent or significant disability/incapacity,
- Is a congenital anomaly/birth defect,
- Any other event considered as “medically significant “.

- **Unexpected adverse reaction**

An adverse reaction whose nature, severity or outcome is not consistent with the available information about the product(s), act(s) or procedure(s) added by the clinical trial.

According to the notice to sponsors of clinical trials not conducted on health-product (ANSM):

- **New safety issue**

It is any new information about safety:

- That could significantly alter the assessment of the benefit-risk ratio for the clinical trial;
- Or which can lead to a modification the trial documents or can alter the conduct of the clinical trial.

For instance, it can be:

- A clinically significant increase in the frequency of occurrence of an expected serious adverse reaction;
- A premature termination or a temporary interruption due to safety reasons of a trial conducted in another country concerning the same product (procedure or methodology) as the trial conducted France.
- Recommendations from the Data Safety Monitoring Board (DSMB) that may affect the safety of the clinical trial subjects.
- Suspected unexpected serious adverse reaction (SUSAR) occurring in subjects who have completed the trial and which are notified to the sponsor by the investigator as well as the follow-up reports.

9.2 The investigator’s roles

The investigator must notify the sponsor, immediately on the day when the sponsor becomes aware, of all the SAE, except those that are listed in the protocol (see. section 9.3) or in the investigator's brochure as not requiring immediate notification.

These SAE are recorded in the "adverse event" section of the CCRF and the investigator must immediately notify the sponsor's Vigilance division (see 9.4).

The investigator’s other roles

The investigator must document the SAE as thoroughly as possible and provide the medical diagnosis, if possible using a specific SAE form **(Annex 8)**.

The investigator assesses the severity of the AE:

- *Mild: tolerated by the patient, does not interfere with daily activities*
- *Moderate: sufficiently uncomfortable to affect daily activities*
- *Serious: preventing daily activities*

The investigator must **assess the causality relationship** between the SAEs and the clinical trial.

The method used by the investigator is based on the WHO method (WHO Uppsala Monitoring Centre), and includes the following four causality terms:

- Certain
- Probable/Likely
- Possible
- Unlikely (not excluded)

Their definition is provided in the table below (from WHO-UMC causality categories, version from 17-Apr-2012).

Table WHO-UMC causality categories (extract)

**Causality term**	Assessment criteria*
**Certain**	· Event or laboratory test abnormality, with plausible time relationship to drug intake<br>· Cannot be explained by disease or other drugs<br>· Response to withdrawal plausible (pharmacologically, pathologically)<br>· Event definitive pharmacologically or phenomenologically (i.e. an objective and specific medical disorder or a recognized pharmacological phenomenon)<br>· Rechallenge satisfactory, if necessary
**Probable / Likely**	· Event or laboratory test abnormality, with reasonable time relationship to drug intake<br>· Unlikely to be attributed to disease or other drugs<br>· Response to withdrawal clinically reasonable<br>· Rechallenge not required
**Possible**	· Event or laboratory test abnormality, with reasonable time relationship to drug intake<br>· Could also be explained by disease or other drugs<br>· Information on drug withdrawal may be lacking or unclear
**Unlikely**	· Event or laboratory test abnormality, with a time to drug intake<br>· that makes a relationship improbable (but not impossible)

	· Disease or other drugs provide plausible explanations

*All points should be reasonably complied with

9.3 Specific features of the protocol

All serious and non-serious AEs are collected using an open-ended question and must be reported in the dedicated section of the CRF.

SAEs that do not require the investigator to immediately notify the sponsor

These SAEs are only recorded in the "adverse event" section of the eCRF. They include the events associated with:

- Normal and natural evolution of the pathology, for example:
- – Scheduled medical visit for the follow-up of knee OA
- – Scheduled hospitalization for the routine treatment of knee OA(joint injection, rehabilitation), and not related to a worsening of the condition
- – Expected symptoms secondary to knee OA worsening : joint pain, joint effusion, OA flare, walking difficulties, surgical knee joint replacement for OA

- Special circumstances
- – Hospitalisation for pre-existing condition
- – Hospitalization for intervention or surgery hospitalisation scheduled prior to the research
- – Hospital admission for social or administrative purpose
- – Admission to the emergency room less than 12 hrs

- AEs likely to be associated with the treatments prescribed as part of the patient's care during the monitoring of the research,
  - o AE related to rescue medications (analgesics and NSAIDs)
  - o AE related to joint injections (hyaluronic and corticosteroids): increased pain, joint swelling, and mild joint effusion that can last a few days, and skin flush following corticosteroid injections that can last a few hours. Exceptionally, septic arthritis or allergic reaction.

These adverse reactions should be reported by the investigator to the regional pharmacovigilance center (CRPV) of which it depends.

SAEs that require the investigator to immediately notify the sponsor

The investigator must report all AEs that meet one of the seriousness criteria below:

1- Death

2- Life threatening situation

3- Requiring hospitalisation or prolonging hospitalisation

4- Persistent or significant disability or incapacity

5- Congenital abnormality or birth defect

6- Or any other AE considered "medically significant"

9.4 Procedures and deadlines for notifying the sponsor

Notification of an SAE must initially be provided in a written report using the special form for reporting SAE. The report must be signed by the investigator.

Each item in the form must be completed by the investigator so that the sponsor can carry out the appropriate analysis.

This initial notification must be followed by one or more detailed follow-up report(s), in writing and signed, within a maximum of 8 days in the case of a fatal or life-threatening event and within 15 days for all other cases.

Whenever possible, the investigator will provide the sponsor with any documents that may be useful (medical reports, laboratory test results, results of additional exams, etc.). These documents must be made anonymous. In addition, the documents must include the following: research acronym, number and initials of the subject, nature and date of the SAE.

Any AE will be monitored until fully resolved (stabilisation at a level considered acceptable by the investigator, or return to the previous state) even if the subject has left the trial.

The initial notification, the SAE follow-up reports and all other documents must be sent to the sponsor via email to the Vigilance Division of the DRCI (eig-vigilance.drc@aphp.fr). It should be noted that it is possible to send SAE reports to the Safety Department by fax to +33 (0)1 44 84 17 99 only in the event of a failed attempt to send the SAE report by email (in order to avoid duplication).

For this study using e-CRF:

- the investigator completes the SAE notification form in the e-CRF, validates, prints and signs the form before sending it *via* fax.

- if it is not possible to connect to the e-CRF, the investigator will complete, sign and send the SAE notification form**.** As soon as the connection is restored, the SAE notification form in the e-CRF must be duly completed.

The investigator must comply with all requests from the sponsor for additional information.

For all questions relating to the notification of an AE, the Vigilance Division of the DRCI can be contacted via email: vigilance.drc@aphp.fr

9.5 Period for notifying the sponsor

The investigator must report all SAE that occur in research subjects:

- on the date of the first administration of investigational product
- throughout the period during which the participant is monitored, as determined by the research

9.6 The sponsor's roles

The sponsor represented by the Vigilance department continuously assesses the safety of the clinical trial throughout the duration of the clinical trial.

Analysis and declaration of SAEs

The sponsor is responsible for the assessment of the following:

- the **seriousness** of all AEs reported,
- the **causality relationship** between the SAE and the acts/procedures/tests added by the clinical trial,
  All SAEs considered by the investigator and/or the sponsor to be possibly related to the act/procedures/tests/products administered, specific to the clinical trial can be reasonably considered as suspected adverse reaction.
- **Expectedness or unexpectedness** of adverse reactions.

  Any adverse reaction whose nature, severity or outcome is not consistent with the information relative to the acts/procedures/ and or products administered during the clinical study is considered unexpected.

  The assessment of the expected/unexpected nature of an adverse is performed by the sponsor represented by the Vigilance department. This assessment is done according the information described below

**Unlikely AEs related** to resveratrol have been reported: headache, myalgia of the lower extremities, somnolence, epidymitis, dizziness, nasopharyngitis, and erythematous rash. Nephrotoxicity was reported in *in vivo* animal studies.
Refer to:<br>- Dossier Technique Investigateur provided to investigators.<br>- Charles-Henry Cottart and al. *Resveratrol bioavailability and toxicity in humans*. Mol. Nutr. Food Res. 2010, 54, 1-10

The sponsor declares any suspected unexpected serious adverse reaction (SUSAR), within the legal deadline, to the Agence nationale de sécurité du médicament et des produits de santé (ANSM, French Health Products Safety Agency) and the concerned Comité de Protection des Personnes (CPP, ethical committee):

- The initial report must be made within 7 calendar days from the date of receipt of the SAE.
- All complementary information must be reported by the sponsor in the form of follow-up reports, within a period of 7 calendar days from the date of receipt of the complementary information.

The sponsor must inform all the concerned investigators of any data which could affect the safety of the participants.

Special case of double-blinded studies:

After unblinding, if the administered product is the investigational product, the case will be immediately declared as suspected serious unexpected adverse reaction (SUSAR); however, if the administered product is the comparator, the unexpected nature of the adverse reaction will be re-assessed according to the reference document of the comparator found in the protocol. In the exceptional case of a clinical study about a disease with high mortality or morbidity, during the clinical study application, the sponsor may request to the ANSM a readjustment of the conditions for unblinding and reporting suspected adverse reaction.

Analysis and declaration of other safety data

This relates to any safety data or new safety issue that could significantly alter the assessment of the benefit-risk ratio of the clinical trial, or could lead to modification in the conduct of the study.

The reporting of safety issues to the competent authorities must be carried out by the sponsor without delay and at the latest within seven calendar days following the day of knowledge.

Following the initial reporting of a new safety issue, the sponsor must send follow-up reports for any relevant additional information relative to this safety issue at the latest within seven calendar days following the day of knowledge.

Annual safety report

The sponsor must prepare once yearly throughout the duration of the clinical trial an annual safety report (ASR) that includes:

- an analysis of participants' safety,

- a list of all suspected serious adverse reactions for the concerned trial that occurred in France during the covered period of the report,
- summary tables of all the SAEs which have occurred in the trial concerned.

Special case: for trials that pose minor risks, that have no influence on subjects' medical care, that do not involve the administration of an investigational product or the practice of an experimental act, the safety report is provided in a simplified letter. The simplified letter of the annual safety report contains a list of suspected adverse reactions that occurred during the covered period of the report, a global analysis and an updated assessment of benefits and risks of the clinical trial.

The annual safety report must be submitted within 60 days after the birth date corresponding to the date of inclusion of the first trial subject.

9.7 Data Safety Monitoring Board (DSMB)

The DSMB can be established by the sponsor. Its primary mission is to serve as a committee for monitoring safety data. It can have other missions, such as monitoring efficacy data (especially if the protocol includes interim analyses). The DSMB is mentioned in Article L. 1123-7 of the French Public Health Code.

A Data and Safety Monitoring Board (DSMB) will not be convened for the present biomedical research

10 DATA MANAGEMENT

10.1 Data collection methods

Information required in the research protocol must be collected in the CRF and an explanation must be given by the investigator for each missing data.
Data must be reported in the electronic CRF when they are available, for clinical or para-clinical data. Correction of discordant data on CRF will be asked through queries. In the CRF, the changes in the data will be tracked.

Anonymization of the patients will ensured using a code number and initials, reported on each needed documents for the research, or by erasing nominative data on copies of source documents.

10.2 Right to access source data and documents

Access to data

In accordance with GCPs:

- the sponsor is responsible for obtaining the permission of all parties involved in the research to guarantee direct access to all locations where the research will be carried out, to the source data, to the source documents and the reports, with the goal of quality control and audit by the sponsor
- the investigators will make available to those in charge of monitoring, quality control and audit relating to the biomedical research the documents and personal data strictly necessary for these controls, in accordance with the legislative and regulatory provisions in force (Articles L.1121-3 and R.5121-13 of the French Public Health Code)

Source documents

Source documents are defined as any original document or object that can prove the existence or accuracy of a piece of information or a fact recorded during the research. These documents will be kept for 15 years by the investigator or by the hospital in the case of a hospital medical file.

Data confidentiality

Those responsible for biomedical research quality control (Article L.1121-3 of the French Public Health Code) will take all necessary precautions to ensure the confidentiality of information about the experimental medications, the research, the research subjects and in particular the identity of the subjects and the results obtained.

These individuals, as well as the investigators themselves, are subject to professional secrecy (in accordance with the conditions set out in Articles 226-13 and 226-14 of the Penal Code).

During or after the biomedical research, the data collected about the research subjects and sent to the sponsor by the investigators (or any other specialised parties) will be made non-identifying.

Under no circumstances should the names and addresses of the subjects involved be shown.

The sponsor will ensure that each research subject has given permission in writing for access to personal information about him or her which is strictly necessary for the quality control of the research.

10.3 Data processing and storage of documents and data

Data entry

Data entry will be carried out on an eCRF, filled in on the internet after each visit by the investigator-physicians in each centre. Access to the on-line data entry form by the investigator-physicians will be restricted by an access code and a personal and unique password system for each

user. Each investigator will, in addition, have access to a specific profile that attributes or withholds access to certain functions of the system (entering data, or simply viewing the data of the enrolled patient or all the study data, possibility of change and validation by the CRAs, etc.…). Data will be stored on a secure server, with data encrypted during transmission and automatic internal saving of a copy on the server that will host the eCRF.

Data processing (CNIL, the French Data Protection Authority) in France

This research falls under the "Méthodologie de référence" (MR-001) according to the provisions of Article 54, paragraph 5 of modified Law No. 78-17 of 6 January 1978 relating to information technology, data files and privacy. This change was approved in a decision made on 5 January 2006. AP-HP, the research sponsor, has signed a commitment to comply with this "Méthodologie de référence".

Archival

Specific documents for biomedical research will be archived by the investigator and the sponsor for a period of 15 years after the end of the research.

10.4 Ownership of the data

AP-HP is the owner of the data, which cannot be used or disclosed to a third party without its prior approval.

11 QUALITY CONTROL AND ASSURANCE

Each biomedical research project managed by AP-HP is ranked from A to D according to the projected risk incurred by research subjects using the classification of biomedical research sponsored by AP-HP.

11.1 General organisation

The sponsor must be responsible for the safety and respect of those subjects who have agreed to participate in the research. The sponsor must implement a quality assurance system to best monitor the conduct of the research in the investigation centres.

For this purpose, the sponsor shall delegate Clinical Research Associates (CRA) whose primary role is to carry out regular follow-up visits at the research locations, after having carried out initial visits.

The objective of monitoring the research, as defined in the French Good Clinical Practices (BPC section 5.18.1), are to verify that:

- The rights, safety and protection of the research subjects are met,

- The data reported is exact, complete and consistent with the source documents,
- The research is carried out in accordance with the protocol in force, with French GCPs and with the legislative and regulatory provisions in force.

11.2 Level of centre monitoring

In the case of this research, which is considered A risk, the appropriate monitoring level will be determined based on the complexity, the impact and the budget for the research. Thus, the sponsor and the coordinating investigator have agreed on the logistic score and impact, resulting in a research monitoring level to be implemented.

11.3 Quality control

A Clinical Research Associate (CRA) appointed by the sponsor will be responsible for the proper conduct of the research, for collecting and documenting, recording and reporting the data generated in writing, in accordance with the Standard Operating Procedures applied within the DRCI and in accordance with the French Good Clinical Practices as well as with the legislative and regulatory provisions in force.

The investigator and the members of the investigator's team agree to make themselves available during Quality Control visits carried out at regular intervals by the Clinical Research Associate. During these visits, the following elements will be reviewed:

- written consent
- compliance with the research protocol and with the procedures defined therein
- quality of the data collected in the CRF: accuracy, missing data, consistency of the data with the "source" documents (medical files, appointment books, original copies of laboratory results, etc.)
- management of the treatments used

11.4 Case Report Form (CRF)

All information required according to the protocol must be entered in the CRF. The data must be collected as and when they are obtained, and clearly recorded in these CRF. Each missing data item must be coded.

This digital CRF will be implemented in each of the centres thanks to a web-based data collection medium. Investigators will be given a document offering guidance in using this tool.

When the investigators complete the case report via the Internet, the CRA can view the data quickly and remotely. The investigator is responsible for the accuracy, quality and relevance of all the data

entered. In addition, the data are immediately verified as they are entered, thanks to consistency checks. Thus, the investigator must validate any changes to the values in the CRF. These modifications will be subject to an audit trail. A justification can be added when applicable, as a comment. A print-out, authenticated (signed and dated) by the investigator, will be requested at the end of the research. The investigator must archive a copy of the authenticated document that was delivered to the sponsor.

11.5 Management of non-compliances

Any events that occur as a result of non-compliance, by the investigator or any other individual involved in conducting the research, with the protocol, with the standard operating procedures, with the good clinical practices or with the legislative and regulatory provisions in force must be noted in a declaration of non-compliance addressed to the sponsor. As a first step, major or critical non-compliances will be reviewed and processed by the DRCI 's medical coordinator in order to implement the necessary corrective or preventive actions. Next, the non-compliances will be sent to the Quality - Risk Management Division of the DRCI for verification and analysis. These verifications could result in the investigator in charge of the research location in question being asked for information or could lead to compliance or audit visits.

11.6 Audits/inspections

The investigators agree to accept the quality assurance audits carried out by the sponsor as well as the inspections carried out by the competent authorities. All data, documents and reports may be subject to regulatory audits and inspections. Medical secrecy cannot be invoked in opposition to these audits and inspections.

An audit can be carried out at any time by individuals appointed by the sponsor and who are not associated with the research directors. The objective of the audit is to ensure the quality of the research, the validity of the results and compliance with the legislation and regulations in force.

The individuals who lead and monitor the research agree to comply with the sponsor's requirements and with the competent authority regarding research audits or inspections.

The audit may be applicable to all stages of the research, from the development of the protocol to the publication of the results and the organisation of the data used or produced as part of the research.

11.7 Primary investigator's commitment to assume responsibility

Before starting the research, each investigator will give the sponsor’s representative a copy of his/her personal curriculum vitæ, signed and dated, with his/her number in the RPPS (*Répertoire Partagé des Professionnels de Santé*, Collective Database of Health Professionals).

Each investigator will undertake to comply with the legislation and to carry out the research according to French GCP, adhering to the Declaration of Helsinki terms in force.

The primary investigator at each participating centre will sign a responsibility commitment (standard DRCI document) which will be sent to the sponsor's representative.

The investigators and their employees will sign a delegation of duties form specifying each person's role.

12 ETHICAL AND LEGAL CONSIDERATIONS

12.1 Methods for obtaining information and consent from research participants

In accordance with Article L1122-1-1 of the French Public Health Code, no biomedical research can be carried out on a person without free and informed consent, obtained in writing after the person has been given the information specified in Article L.1122-1 of said Code.

The free and informed consent, in writing, of the subject is obtained by the investigator, or by a doctor representing the investigator, before the inclusion of the subject in the research.
The information sheet and a copy of the consent form signed and dated by the research subject and by the investigator or the doctor representing the investigator, are given to the individual prior to his or her participation in the research.

In addition, the investigator will specify in the research participant's medical file the methods used for obtaining his or her consent as well as the methods used for providing information with the goal of obtaining their consent. The investigator will retain the original signed and dated copy of the subject's consent form.

12.2 Subject prohibited from participating in another research or an exclusion period anticipated after the research, if applicable

The subject will be prohibited from participating in other biomedical research protocols relating to medications during the whole study duration, in order to avoid interferences with the results of the study. There will be no exclusion period anticipated after the research.

12.3 Compensation for subjects

No compensation is anticipated for the patients/control subjects as compensation for the inconveniences relating to the research.

12.4 Legal obligations

The sponsor's role

Assistance Publique - Hôpitaux de Paris (AP-HP) is the sponsor of this research and by delegation, the Clinical Research and Innovation Department (DRCI) carries out the research's missions in accordance with Article L.1121-1 of the French Public Health Code. Assistance Publique - Hôpitaux de Paris reserves the right to halt the research at any time for medical or administrative reasons. In this case, notification will be sent to the investigator.

Request for an opinion from the Comité de Protection des Personnes (CPP, ethical review board)

AP-HP, as sponsor, obtains for this biomedical research relating to a medication for human use and prior to starting the research, the favourable opinion of the appropriate CPP, within the scope of its authority and in accordance with the legislative and regulatory provisions in force.

Request for authorisation to ANSM

AP-HP, as sponsor, obtains for this biomedical research relating to a medication for human use and prior to starting the research, authorisation from the ANSM, within the scope of its authority and in accordance with the legislative and regulatory provisions in force.

Procedures for Data Protection regulation

The computer file used for this research is implemented in accordance with the French regulation (the modified French Data Protection Act) and the European regulation (General Data Protection Regulation – GDPR).

This research is part of the "reference methodology for the processing of personal data implemented in the context of health research" (Modified MR-001).

12.5 Modifications to the research

Any substantial modification to the protocol by the coordinating investigator must be sent to the sponsor for approval. After approval is given, the sponsor must obtain, prior to starting the research, a favourable opinion from the CPP and authorisation from the ANSM within the scope of their respective authorities.

The information sheet and the consent form can be revised if necessary; in particular if there is a substantial modification to the research or if adverse reactions occur.

12.6 Final research report

The final biomedical research report referred to in Article R1123-60 of the French Public Health Code is drawn up and signed by the sponsor and the investigator. A summary of the report written according to the competent authority's reference plan will need to be sent to the competent authority and ethical review board within one year after the end of the research, meaning the end of the participation of the last research subject.

13 FUNDING AND INSURANCE

13.1 Funding source

The research is funded by the 2015 Programme Hospitalier de Recherche Clinique (grant n° 15-15-0234 of the French Ministry of Health).

13.2 Insurance

For the duration of the research, the Sponsor will take out an insurance policy covering the sponsor's own civil liability as well as the civil liability of all the doctors involved in carrying out the research. The sponsor will also provide full compensation for all harmful consequences of the research for the research subjects and their beneficiaries, unless the sponsor can prove that the harm is not the fault of the sponsor or any agent. The act of a third party or the voluntary withdrawal of the person who initially consented to participate in the research cannot be invoked against said compensation.

Assistance Publique- Hôpitaux de Paris (AP-HP) has taken out insurance from HDI-GERLING through BIOMEDIC-INSURE for the full research period, covering its own civil liability and that of any agent (doctor or research staff), in accordance with Article L.1121-10 of the French Public Health Code.

14 PUBLICATION RULES

14.1 Mention of the AP-HP manager (DRCI) in the acknowledgements of the text

"The sponsor was Assistance Publique - Hôpitaux de Paris (Département de la Recherche Clinique et de l'Innovation, Clinical Research and Innovation Department)"

14.2 Mention of the financier in the acknowledgements of the text

The following statement will be included in all publications "The research was funded by a grant from Programme Hospitalier de Recherche Clinique - PHRC 2015 (Ministère de la Santé)".

This research has been registered on the website http://clinicaltrials.gov: (ClinicalTrials.gov Identifier: NCT02905799; first received: September19, 2016; last updated: February04,2020).

15 BIBLIOGRAPHY

1. DALYs GBD, Collaborators H, Murray CJ, Barber RM, Foreman KJ, Ozgoren AA, et al. Global, regional, and national disability-adjusted life years (DALYs) for 306 diseases and injuries and healthy life expectancy (HALE) for 188 countries, 1990-2013: quantifying the epidemiological transition. Lancet. 2015. doi: 10.1016/S0140-6736(15)61340-X. PubMed PMID: 26321261.
2. Bijlsma JW, Berenbaum F, Lafeber FP. Osteoarthritis: an update with relevance for clinical practice. Lancet. 2011;377(9783):2115-26. doi: 10.1016/S0140-6736(11)60243-2. PubMed PMID: 21684382.
3. Palazzo C, Ravaud JF, Papelard A, Ravaud P, Poiraudeau S. The burden of musculoskeletal conditions. PLoS One. 2014;9(3):e90633. doi: 10.1371/journal.pone.0090633. PubMed PMID: 24595187; PubMed Central PMCID: PMC3942474.
4. Richette P, Latourte A, Frazier A. Safety and efficacy of paracetamol and NSAIDs in osteoarthritis: which drug to recommend? Expert Opin Drug Saf. 2015;14(8):1259-68. doi: 10.1517/14740338.2015.1056776. PubMed PMID: 26134750.
5. Roberts E, Delgado Nunes V, Buckner S, Latchem S, Constanti M, Miller P, et al. Paracetamol: not as safe as we thought? A systematic literature review of observational studies. Ann Rheum Dis. 2015. doi: 10.1136/annrheumdis-2014-206914. PubMed PMID: 25732175.
6. Vang O, Ahmad N, Baile CA, Baur JA, Brown K, Csiszar A, et al. What is new for an old molecule? Systematic review and recommendations on the use of resveratrol. PLoS One. 2011;6(6):e19881. doi: 10.1371/journal.pone.0019881. PubMed PMID: 21698226; PubMed Central PMCID: PMC3116821.
7. Amiot MJ, Romier B, Dao TM, Fanciullino R, Ciccolini J, Burcelin R, et al. Optimization of trans-Resveratrol bioavailability for human therapy. Biochimie. 2013;95(6):1233-8. doi: 10.1016/j.biochi.2013.01.008. PubMed PMID: 23376875.

8. Renaud S, Ruf JC. The French paradox: vegetables or wine. Circulation. 1994;90(6):3118-9. Epub 1994/12/01. PubMed PMID: 7994864.
9. Constant J. Alcohol, ischemic heart disease, and the French paradox. Coronary artery disease. 1997;8(10):645-9. Epub 1998/02/11. PubMed PMID: 9457446.
10. Soleas GJ, Diamandis EP, Goldberg DM. Resveratrol: a molecule whose time has come? And gone? Clinical biochemistry. 1997;30(2):91-113. Epub 1997/03/01. PubMed PMID: 9127691.
11. Fremont L. Biological effects of resveratrol. Life sciences. 2000;66(8):663-73. Epub 2000/02/19. PubMed PMID: 10680575.
12. Jang M, Cai L, Udeani GO, Slowing KV, Thomas CF, Beecher CW, et al. Cancer chemopreventive activity of resveratrol, a natural product derived from grapes. Science (New York, NY). 1997;275(5297):218-20. Epub 1997/01/10. PubMed PMID: 8985016.
13. Smoliga JM, Baur JA, Hausenblas HA. Resveratrol and health--a comprehensive review of human clinical trials. Mol Nutr Food Res. 2011;55(8):1129-41. doi: 10.1002/mnfr.201100143. PubMed PMID: 21688389.
14. Cottart CH, Nivet-Antoine V, Beaudeux JL. Is resveratrol an imposter? Mol Nutr Food Res. 2015;59(1):7. doi: 10.1002/mnfr.201570005. PubMed PMID: 25558005.
15. Visioli F. The resveratrol fiasco. Pharmacol Res. 2014;90:87. doi: 10.1016/j.phrs.2014.08.003. PubMed PMID: 25180457.
16. Yang FM, Friedrichs WE, Cupples RL, Bonifacio MJ, Sanford JA, Horton WA, et al. Human ceruloplasmin. Tissue-specific expression of transcripts produced by alternative splicing. J Biol Chem. 1990;265(18):10780-5. PubMed PMID: 2355023.
17. Dave M, Attur M, Palmer G, Al-Mussawir HE, Kennish L, Patel J, et al. The antioxidant resveratrol protects against chondrocyte apoptosis via effects on mitochondrial polarization and ATP production. Arthritis Rheum. 2008;58(9):2786-97. doi: 10.1002/art.23799. PubMed PMID: 18759268.
18. Li X, Phillips FM, An HS, Ellman M, Thonar EJ, Wu W, et al. The action of resveratrol, a phytoestrogen found in grapes, on the intervertebral disc. Spine (Phila Pa 1976). 2008;33(24):2586-95. doi: 10.1097/BRS.0b013e3181883883. PubMed PMID: 19011540; PubMed Central PMCID: PMC2905225.
19. Malemud CJ. Inhibitors of stress-activated protein/mitogen-activated protein kinase pathways. Curr Opin Pharmacol. 2007;7(3):339-43. doi: 10.1016/j.coph.2006.11.012. PubMed PMID: 17398158.
20. Mengshol JA, Vincenti MP, Coon CI, Barchowsky A, Brinckerhoff CE. Interleukin-1 induction of collagenase 3 (matrix metalloproteinase 13) gene expression in chondrocytes requires p38, c-Jun N-terminal kinase, and nuclear factor kappaB: differential regulation of collagenase 1 and collagenase 3. Arthritis Rheum. 2000;43(4):801-11. doi: 10.1002/1529-0131(200004)43:4<801::AID-ANR10>3.0.CO;2-4. PubMed PMID: 10765924.
21. Shakibaei M, Mobasheri A, Buhrmann C. Curcumin synergizes with resveratrol to stimulate the MAPK signaling pathway in human articular chondrocytes in vitro. Genes Nutr. 2011;6(2):171-9.

doi: 10.1007/s12263-010-0179-5. PubMed PMID: 21484156; PubMed Central PMCID: PMC3092909.

22. Csaki C, Mobasheri A, Shakibaei M. Synergistic chondroprotective effects of curcumin and resveratrol in human articular chondrocytes: inhibition of IL-1beta-induced NF-kappaB-mediated inflammation and apoptosis. Arthritis Res Ther. 2009;11(6):R165. doi: 10.1186/ar2850. PubMed PMID: 19889203; PubMed Central PMCID: PMC3003513.

23. Lei M, Liu SQ, Liu YL. Resveratrol protects bone marrow mesenchymal stem cell derived chondrocytes cultured on chitosan-gelatin scaffolds from the inhibitory effect of interleukin-1beta. Acta Pharmacol Sin. 2008;29(11):1350-6. doi: 10.1111/j.1745-7254.2008.00880.x. PubMed PMID: 18954530.

24. Liu FC, Hung LF, Wu WL, Chang DM, Huang CY, Lai JH, et al. Chondroprotective effects and mechanisms of resveratrol in advanced glycation end products-stimulated chondrocytes. Arthritis Res Ther. 2010;12(5):R167. doi: 10.1186/ar3127. PubMed PMID: 20825639; PubMed Central PMCID: PMC2990994.

25. Shakibaei M, Csaki C, Nebrich S, Mobasheri A. Resveratrol suppresses interleukin-1beta-induced inflammatory signaling and apoptosis in human articular chondrocytes: potential for use as a novel nutraceutical for the treatment of osteoarthritis. Biochem Pharmacol. 2008;76(11):1426-39. doi: 10.1016/j.bcp.2008.05.029. PubMed PMID: 18606398.

26. Tian J, Chen JW, Gao JS, Li L, Xie X. Resveratrol inhibits TNF-alpha-induced IL-1beta, MMP-3 production in human rheumatoid arthritis fibroblast-like synoviocytes via modulation of PI3kinase/Akt pathway. Rheumatol Int. 2013;33(7):1829-35. doi: 10.1007/s00296-012-2657-0. PubMed PMID: 23328930.

27. Byun HS, Song JK, Kim YR, Piao L, Won M, Park KA, et al. Caspase-8 has an essential role in resveratrol-induced apoptosis of rheumatoid fibroblast-like synoviocytes. Rheumatology (Oxford). 2008;47(3):301-8. doi: 10.1093/rheumatology/kem368. PubMed PMID: 18276737.

28. Ferrero ME, Bertelli AE, Fulgenzi A, Pellegatta F, Corsi MM, Bonfrate M, et al. Activity in vitro of resveratrol on granulocyte and monocyte adhesion to endothelium. Am J Clin Nutr. 1998;68(6):1208-14. PubMed PMID: 9846848.

29. Vorderstrasse BA, Steppan LB, Silverstone AE, Kerkvliet NI. Aryl hydrocarbon receptor-deficient mice generate normal immune responses to model antigens and are resistant to TCDD-induced immune suppression. Toxicol Appl Pharmacol. 2001;171(3):157-64. doi: 10.1006/taap.2000.9122. PubMed PMID: 11243915.

30. Elmali N, Esenkaya I, Harma A, Ertem K, Turkoz Y, Mizrak B. Effect of resveratrol in experimental osteoarthritis in rabbits. Inflamm Res. 2005;54(4):158-62. doi: 10.1007/s00011-004-1341-6. PubMed PMID: 15883738.

31. Wang J, Gao JS, Chen JW, Li F, Tian J. Effect of resveratrol on cartilage protection and apoptosis inhibition in experimental osteoarthritis of rabbit. Rheumatol Int. 2012;32(6):1541-8. doi: 10.1007/s00296-010-1720-y. PubMed PMID: 21327438.
32. Li W, Cai L, Zhang Y, Cui L, Shen G. Intra-articular resveratrol injection prevents osteoarthritis progression in a mouse model by activating SIRT1 and thereby silencing HIF-2alpha. J Orthop Res. 2015;33(7):1061-70. doi: 10.1002/jor.22859. PubMed PMID: 25737402.
33. Bellamy N, Kirwan J, Boers M, Brooks P, Strand V, Tugwell P, et al. Recommendations for a core set of outcome measures for future phase III clinical trials in knee, hip, and hand osteoarthritis. Consensus development at OMERACT III. The Journal of rheumatology. 1997;24(4):799-802. PubMed PMID: 9101522.
34. Bellamy N, Buchanan WW, Goldsmith CH, Campbell J, Stitt LW. Validation study of WOMAC: a health status instrument for measuring clinically important patient relevant outcomes to antirheumatic drug therapy in patients with osteoarthritis of the hip or knee. The Journal of rheumatology. 1988;15(12):1833-40. PubMed PMID: 3068365.
35. Pham T, van der Heijde D, Altman RD, Anderson JJ, Bellamy N, Hochberg M, et al. OMERACT-OARSI initiative: Osteoarthritis Research Society International set of responder criteria for osteoarthritis clinical trials revisited. Osteoarthritis and cartilage / OARS, Osteoarthritis Research Society. 2004;12(5):389-99. doi: 10.1016/j.joca.2004.02.001. PubMed PMID: 15094138.
36. Ware J, Jr., Kosinski M, Keller SD. A 12-Item Short-Form Health Survey: construction of scales and preliminary tests of reliability and validity. Medical care. 1996;34(3):220-33. PubMed PMID: 8628042.
37. Conrozier T, Mathieu P, Vignon E, Piperno M, Rinaudo M. Differences in the osteoarthritic synovial fluid composition and rheology between patients with or without flare: a pilot study. Clinical and experimental rheumatology. 2012;30(5):729-34. PubMed PMID: 22935334.
38. Schulz KF, Altman DG, Moher D, Group C. CONSORT 2010 statement: updated guidelines for reporting parallel group randomised trials. Bmj. 2010;340:c332. doi: 10.1136/bmj.c332. PubMed PMID: 20332509; PubMed Central PMCID: PMC2844940.
39. Cottart CH, Nivet-Antoine V, Laguillier-Morizot C, Beaudeux JL. Resveratrol bioavailability and toxicity in humans. Mol Nutr Food Res. 2010;54(1):7-16. doi: 10.1002/mnfr.200900437. PubMed PMID: 20013887.

16 <u>LIST OF ADDENDA</u>

16.1 List of Investigators

Address of the research location	Title	First name Surname	Telephone / e-mail / Fax
Service de Rééducation et de Réadaptation de l'Appareil Locomoteur	MD, PhD	Prof. François Rannou	Tel: +33158412535<br>francois.rannou@aphp.fr

et des Pathologies du Rachis<br>Hôpitaux Universitaires Paris Centre<br>Groupe Hospitalier Cochin<br>27, Rue du Faubourg Saint-Jacques<br>75014 Paris, FRANCE			Fax: +33158412545
Service de Rhumatologie<br>Hôpitaux Universitaires Est Parisien<br>Groupe Hospitalier Saint-Antoine<br>184, Rue du Faubourg Saint-Antoine<br>75012 Paris, FRANCE	MD, PhD	Prof. Francis Berenbaum	Tel: +33149282520<br>francis.berenbaum@aphp.fr<br>Fax: +33149282513
Service de Médecine Physique et de Réadaptation<br>Université d'Auvergne<br>CHU Clermont-Ferrand<br>CHU Hôpital Nord<br>61, Rue de Chateaugay<br>BP 30056, 63118 Cébazat, FRANCE	MD, PhD	Prof. Emmanuel Coudeyre	Tel: +33473750900<br>ecoudeyre@chu-clermontferrand.fr<br>Fax: +33473750901

16.2 Annexes

Annex 1. 11-point self-administered pain numeric rating scale (0, no pain - 100, maximal pain) used at baseline, 3- and 6-month visits

Quelle a été l'intensité moyenne de votre douleur du genou au cours de ces dernières 48 heures ?

Aucune douleur										Douleur maximale
☐	☐	☐	☐	☐	☐	☐	☐	☐	☐	☐
**0**	**10**	**20**	**30**	**40**	**50**	**60**	**70**	**80**	**90**	**100**

Annex 2. French version of the physical function subscore of the WOMAC self-administered questionnaire [34] **used at baseline, 3- and 6-month visits**

Pour chacune des activités suivantes, veuillez préciser les difficultés éprouvées en raison de votre articulation souffrante, **au cours de ces dernières 48 heures**.

SECTION FONCTION						SECTION PERSONNALISÉE
Quelle est l'importance de la difficulté que vous éprouvez à :	**Aucune 0**	**Minime 1**	**Modérée 2**	**Sévère 3**	**Très sévère 4**	**Cochez les 5 items choisis par le patient**
1. Descendre les escaliers	❑	❑	❑	❑	❑	❑
2. Monter les escaliers	❑	❑	❑	❑	❑	❑
3. Vous relever de la position assise	❑	❑	❑	❑	❑	❑
4. Vous tenir debout	❑	❑	❑	❑	❑	❑
5. Vous pencher en avant	❑	❑	❑	❑	❑	❑
6. Marcher en terrain plat	❑	❑	❑	❑	❑	❑
7. Entrer et sortir d'une voiture	❑	❑	❑	❑	❑	❑
8. Faire vos courses	❑	❑	❑	❑	❑	❑
9. Enfiler vos collants ou vos chaussettes	❑	❑	❑	❑	❑	❑
10. Sortir du lit	❑	❑	❑	❑	❑	❑
11. Enlever vos collants ou vos chaussettes	❑	❑	❑	❑	❑	❑
12. Vous étendre sur le lit	❑	❑	❑	❑	❑	❑
13. Entrer ou sortir d'une baignoire	❑	❑	❑	❑	❑	❑
14. Vous asseoir	❑	❑	❑	❑	❑	❑
15. Vous asseoir et vous relever des toilettes	❑	❑	❑	❑	❑	❑
16. Faire le ménage « à fond » de votre domicile	❑	❑	❑	❑	❑	❑
17. Faire l'entretien quotidien de votre domicile	❑	❑	❑	❑	❑	❑
Somme de chaque colonne	**0**	|__|__| entre 0 et 17	|__|__| entre 0 et 34	|__|__| entre 0 et 51	|__|__| entre 0 et 68	|__|__| entre 0 et 20
Total	|__|__| entre 0 et 68					|__|__| entre 0 et 20
Score	= (Total/17)*25 |__|__|__| (0-100)					= (Total/5)*25 |__|__|__| (0-100)

Annex 3. 11-point self-administered numeric rating scale for patient's global assessment (0 worst possible - 100 best possible) used at baseline, 3- and 6-month visits

Quel est votre état de santé global actuel ?

**Le pire possible**										**Le meilleur possible**
☐	☐	☐	☐	☐	☐	☐	☐	☐	☐	☐
**0**	**10**	**20**	**30**	**40**	**50**	**60**	**70**	**80**	**90**	**100**

Annex 4. OARSI-OMERACT set of responder criteria [35], assessed at 3- and 6-months

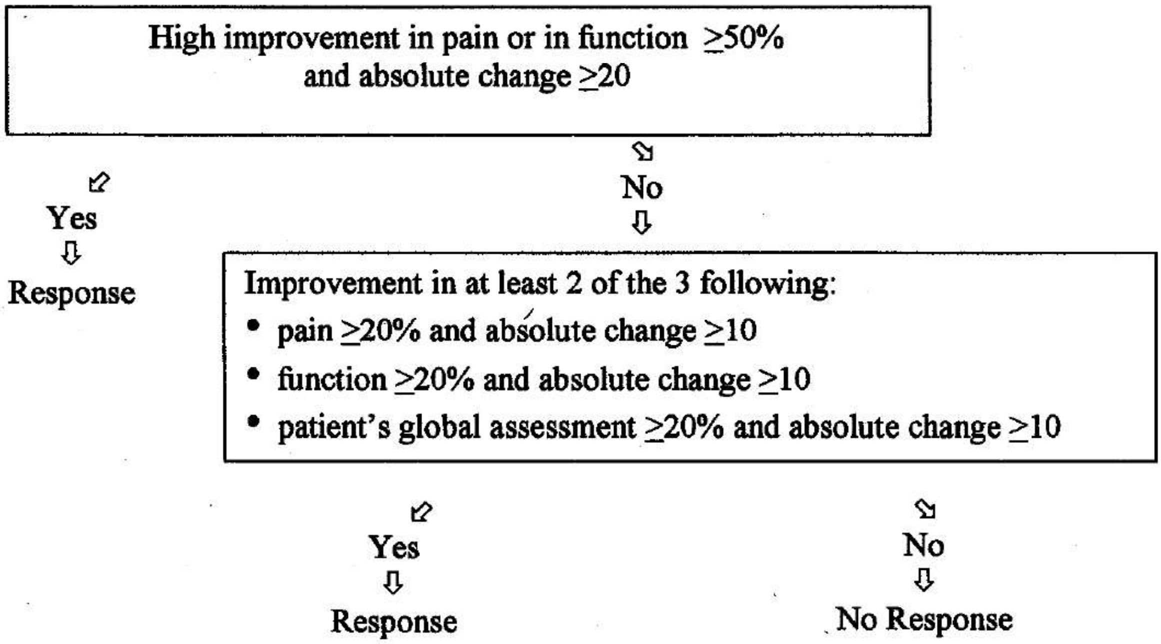

With:

- Pain assessed at baseline, 3- and 6-months using an 11-point self-administered pain NRS
- Function assessed at baseline, 3- and 6-months by the function subscale of the WOMAC self-administered questionnaire
- Patient's global assessment assessed at baseline, 3- and 6-months using an 11-point self-administered global assessment NRS

Annex 5. Self-reported medication recorded at baseline, 3- and 6-months

1. **Combien d'infiltration(s) de corticoïdes et/ou d'acide hyaluronique avez-vous eu dans le genou au cours de ces 3 derniers mois pour le traitement de votre arthrose du genou ?**

☐	☐	☐	☐	☐	☐	☐	☐	☐	☐	☐
**0**	**1**	**2**	**3**	**4**	**5**	**6**	**7**	**8**	**9**	**10**

2. **Quelle quantité moyenne d'antalgiques avez-vous consommés au cours de ces 3 derniers mois pour le traitement de votre arthrose du genou ?**

- **Non morphiniques (par exemple: paracétamol) ?**

☐ **Jamais**
☐ **Plusieurs fois par mois**
☐ **Plusieurs fois par semaine**
☐ **Tous les jours**

- **Opioïdes faibles (par exemple: codéine, dihydrocodéine, tramadol) ?**

☐ **Jamais**
☐ **Plusieurs fois par mois**
☐ **Plusieurs fois par semaine**
☐ **Tous les jours**

- **Opioïdes forts (par exemple: morphine, diamorphine, fentanyl, buprénorphine, oxymorphone, oxycodone, hydromorphone) ?**

☐ **Jamais**
☐ **Plusieurs fois par mois**
☐ **Plusieurs fois par semaine**
☐ **Tous les jours**

3. **Quelle quantité moyenne d'anti-inflammatoires non-stéroïdiens avez-vous consommés au cours de ces 3 derniers mois pour le traitement de votre arthrose du genou ?**

☐ **Jamais**
☐ **Plusieurs fois par mois**
☐ **Plusieurs fois par semaine**
☐ **Tous les jours**

4. Quelle quantité moyenne d'anti-arthrosiques d'action lente (par exemple : chondroïtine sulfate, glucosamine sulfate, insaponifiable d'huile de soja et avocat, diacerhéine) avez-vous consommés au cours de ces 3 derniers mois pour le traitement de votre arthrose du genou ?

☐ **Jamais**
☐ **Plusieurs fois par mois**
☐ **Plusieurs fois par semaine**
☐ **Tous les jours**

Annex 6. Advertising on the internet, in the medias (newspapers, health magazines) and using posters displayed in each investigating centres

Arthrose du genou : l’hôpital Cochin recrute des patients pour une étude clinique

Vous avez une arthrose du genou douloureuse, confirmée par une radiographie, et êtes âgé de plus de 40 ans ? Le Professeur François Rannou, Chef du Service de Rééducation et Réadaptation de l’Appareil Locomoteur et des Pathologies du Rachis (Hôpital Cochin), lance une étude clinique sur l’arthrose du genou. 164 patients doivent être inclus dans cette recherche. Dans le cadre de cette étude, les patients bénéficieront d’une consultation médicale et recevront pendant 6 mois un complément nutritionnel ou un placebo. Si vous souhaitez y participer, envoyez un email à l’adresse de messagerie suivante : etude.arthrol.cch@aphp.fr

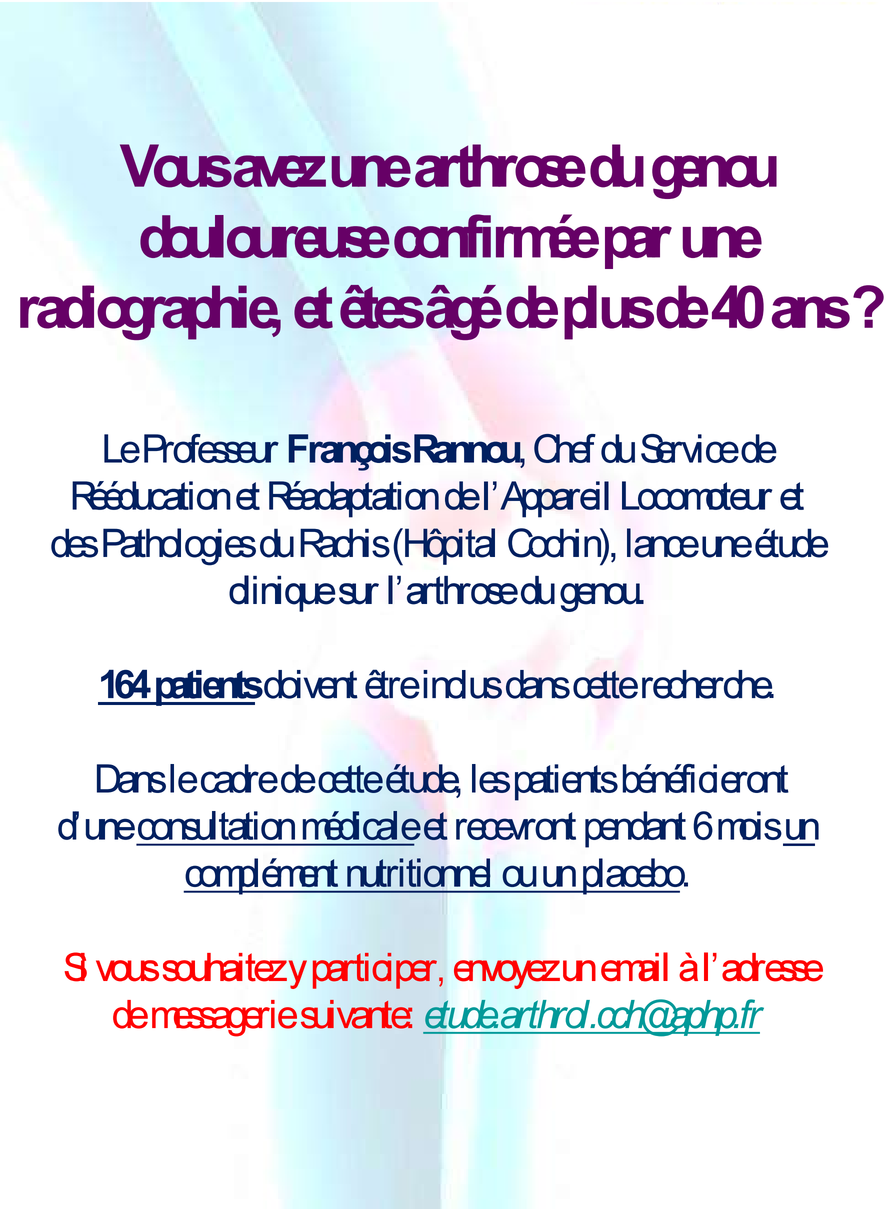

Annex 7. Patient Card

CARTE PATIENT

<u>Merci de garder cette carte en permanence avec vous</u>

Nom :……………………………………. Prénom : …………………………….

Code d'identification : Code d'identification Patient
___ ___ ___ / ___ ___ ___ ___ / ___ ___

N° centre / N° d'inclusion/ Initiales Nom Prénom

Je participe à la recherche : *ARTHROL*
Évaluation des effets du resveratrol per os dans l'arthrose du genou

Dont le promoteur est l'Assistance Publique – Hôpitaux de Paris

Je reçois le traitement suivant : ***Médicament de la recherche* ou *contrôle***

À la dose de : ***2 gélules 2 fois par jour pendant 1 semaine***
1 gélule 2 fois par jour pendant 6 mois

Date de début de traitement : __ __ /__ __ /__ __ __ __

Traitement reçu = Numéro de randomisation attribué° : XXXXXXXXXXXX

CARTE PATIENT

Je suis suivi(e) par le Dr...

À l'Hôpital ....

☎..

En cas de nécessité de connaître votre traitement en urgence, votre médecin peut contacter le Centre Anti-Poison de l'hôpital Fernand Widal, à Paris
☎: 01 40 05 48 48

Annex 8. SAE Form

irection de l'Organisation Médicale<br>: des relations avec les Universités<br>DOMU)<br><br>épartement de la Recherche<br>linique<br>: du Développement (DRCD)	**Formulaire de notification d'un Evènement Indésirable Grave (EIG) survenant au cours d'une Recherche Biomédicale ne portant pas sur un produit de santé**	**PARTIE RESERVEE AU PROMOTEUR**<br><br>**REFERENCE VIGILANCE :**<br><br>Référence GED : REC-DTYP-0271

Dès la prise de connaissance de l'EIG par l'investigateur, ce formulaire doit être dûment complété (3 pages), signé et retourné sans délai au secteur Vigilance de la DRCI par mail (eig-vigilance.drc@aphp.fr). Il est à noter qu'il est possible de transmettre les EIG au secteur Vigilance par télécopie au +33 (0)1 44 84 17 99 uniquement en cas de tentative infructueuse d'envoi des EIG par mail (afin d'éviter les doublons).

Notification initiale ☐ **Suivi d'EIG** ☐ **N° du suivi** |__|__|

**1. Identification de la recherche**	
**Acronyme : ARTHROL**	**Date de notification :** |__|__| |__|__| |_2_|_0_|__|__| (jj mm aaaa)
**Code de la Recherche : P150938**<br>**Risque : A**	**Date de prise de connaissance de l'EIG par l'investigateur :** |__|__| |__|__| |_2_|_0_|__|__| (jj mm aaaa)

Titre complet de la Recherche Biomédicale :

EVOLUTION OF PAIN AT THREE MONTHS BY ORAL RESVERATROL IN PRIMARY KNEE OSTEOARTHRITIS : A MULTICENTER, DOUBLE-BLIND, RANDOMIZED, PLACEBO-CONTROLLED TRIAL

**2. Identification du centre investigateur**	
Nom de l'établissement : ....<br>Ville et code postal : ....<br>Service :	Investigateur (nom/prénom) :<br>............<br>Tél : .... Fax : ....

**3. Identification et antécédents de la personne se prêtant à la recherche**		
Référence de la personne : |__|__|__| - |__|__|__|__|__| - |__| - |__| n° centre - n° ordre de sélection - initiale nom - initiale prénom		**Antécédents médicaux-chirurgicaux/familiaux pertinents pour l'évaluation du cas (joindre un CRH le cas échéant) :**<br>............<br>............<br>............<br>............<br>............<br>............<br>............
Sexe : ☐ M ☐ F<br>Poids : |__|__|__| kg<br>Taille : |__|__|__| cm	Date de naissance :<br>|__|__| |__|__| |__|__|__|__| jj mm aaaa<br>Age : |__|__|__|ans	
Date de signature du consentement :	|__|__| |__|__| |_2_|_0_|__|__| jj mm aaaa	
Date de randomisation :	|__|__| |__|__| |_2_|_0_|__|__| jj mm aaaa	N° traitement ou N° randomisation :

**4. Procédures et actes ajoutés par la recherche** *(ex. : biopsies, IRM … Barrer l'encadré si procédures et actes non réalisés)*	Date de réalisation *(jj/mm/aaaa)*	Chronologie: Avant la survenue de l'EIG	Chronologie: Après la survenue de l'EIG
RESVERATROL ou Placebo	|__|__| |__|__| |_2_|_0_|__|__|	☐	☐
	|__|__| |__|__| |_2_|_0_|__|__|	☐	☐
....	|__|__| |__|__| |_2_|_0_|__|__|	☐	☐
....	|__|__| |__|__| |_2_|_0_|__|__|	☐	☐

5. Médicament(s) concomitant(s) au moment de l'EIG, à l'exclusion de ceux utilisés pour traiter l'événement indésirable

(compléter le tableau ci-après et si nécessaire l'annexe relative aux médicaments concomitants ou barrer l'encadré si non applicable)

➯ **Annexe jointe au présent formulaire :** ☐ Oui ☐ Non

Acronyme : **ARTHROL**

Référence de la personne se prêtant à la recherche : |__|__|__| - |__|__|__|__|__| - |__| - |__|
n° centre - n° ordre de sélection - initiale nom - initiale prénom

PARTIE RESERVEE AU PROMOTEUR

REFERENCE VIGILANCE :

Référence GED : REC-DTYP-0271

Nom commercial (de préférence) ou Dénomination Commune Internationale	Voie(1)	Posologie (préciser l'unité ex : mg/j)	Dates d'administration *(du jj/mm/aa au jj/mm/aa)*	En cours(2)	Indication	***Action prise*** *0 : poursuite sans modification de la posologie 1 : arrêt 2 : diminution de la posologie 3 : augmentation de la posologie 4 : ne sais pas*	***Causalité de l'EIG*** *0 : non lié au médicament 1 : lié au médicament 2 : ne sais pas*
			du |__|__| |__|__| |__|__| au |__|__| |__|__| |__|__|	☐			
			du |__|__| |__|__| |__|__| au |__|__| |__|__| |__|__|	☐			

(1) Voie d'administration : VO=voie orale ; IM=Intramusculaire ; IV=intraveineuse ; SC=sous-cutanée ou autre (à préciser) (2) En cours au moment de la survenue de l'EIG

6. Evènement indésirable grave [EIG]

Diagnostic : ☐ Définitif ☐ Provisoire

....

Date de survenue des premiers symptômes : |__|__| |__|__| |_2_|_0_|__|__|

Préciser lesquels ....

Organe(s) concerné(s) :

....

Date d'apparition de l'EIG :

|__|__| |__|__| |_2_|_0_|__|__|
jj mm aaaa

Heure de survenue : |__||__| hh |__||__| min

☐ *donnée manquante*

Délai entre la date de procédure/acte ajouté par la recherche et la date de survenue de l'EIG :

|__||__| / |__||__| |__||__|
jj hh min

Critères de gravité :

☐ Nécessite ou prolonge l'hospitalisation :

du |__|__| |__|__| |_2_|_0_|__|__|

au |__|__| |__|__| |_2_|_0_|__|__| ☐ *en cours*

☐ Décès

☐ Mise en jeu du pronostic vital

☐ Incapacité ou handicap important ou durable

☐ Anomalie ou malformation congénitale

☐ Autre(s) critère(s) médicalement significatif(s), préciser :

....

Degré de sévérité :

☐ Léger ☐ Modéré ☐ Sévère

L'évènement a-t-il conduit à une interruption de la procédure/acte ajouté par la recherche ?

☐ Non ☐ Oui Date : |__|__| |__|__| |_2_|_0_|__|__| ☐ Non applicable

L'arrêt de la procédure/acte ajouté par la recherche a été :

○ Provisoire ○ Définitif

Le cas échéant, date de reprise de la procédure/acte ajouté par la recherche :

|__|__| |__|__| |_2_|_0_|__|__| ☐ Non applicable

Récidive de l'EIG après reprise de la procédure/acte ajouté par la recherche :

○ Non ○ Oui Date : |__|__| |__|__| |_2_|_0_|__|__| ☐ Non applicable

Des mesures symptomatiques ont-elles été prises ?

☐ Non ☐ Oui Date : |__|__| |__|__| |_2_|_0_|__|__|

Préciser : ....

....

L'évènement a-t-il conduit à une levée d'insu ?

☐ Non ☐ Oui Date : |__|__| |__|__| |_2_|_0_|__|__| Résultat : ……………………………

Evolution de l'événement

☐ **Décès**
○ sans relation avec l'EIG
○ en relation avec l'EIG

Date : |__|__| |__|__| |_2_|_0_|__|__|
jj mm aaaa

☐ **Résolu :**
○ sans séquelles
○ avec séquelles, préciser lesquelles :
....

Date : |__|__| |__|__| |_2_|_0_|__|__|
jj mm aaaa

|__|__| |__|__|
hh min

☐ **Sujet non encore rétabli**, préciser :
○ Etat stable
○ Aggravation
○ Amélioration

☐ **Evolution inconnue**

PARTIE RESERVEE AU PROMOTEUR
REFERENCE VIGILANCE :
Référence GED : REC-DTYP-0271

7. Autre(s) étiologie(s) envisagée(s)

☐ Non ☐ Oui Si oui, préciser : ……………………………………………………………………………
……………………………………………………………………………………………………
……………………………………………………………………………………………………

8. Examen(s) complémentaire(s) réalisé(s)

☐ Non ☐ Oui Si oui, préciser date, nature et résultats : [joindre les bilans anonymisés] ……………………
……………………………………………………………………………………………………
……………………………………………………………………………………………………
……………………………………………………………………………………………………

9. Selon l'investigateur, l'événement indésirable grave est (plusieurs cases possibles)

Lié à la recherche biomédicale :

☐ Oui : ○ **à la (aux) procédure(s)/acte(s) de la recherche biomédicale : resveratrol ou placebo**
☐ Relation certaine ☐ Relation probable ☐ Relation possible ☐ Relation improbable (non exclu)

☐ Non : ○ **à la progression de la maladie faisant l'objet de la recherche :** arthrose du genou
○ **à un (ou plusieurs) médicament(s) concomitant(s) administré(s), le(s)quel(s) :** ……………………
○ **à une maladie intercurrente, laquelle :** ……………………………………………………
○ **autre, préciser :** ……………………………………………………………………

Notificateur	Investigateur	Tampon du service :
Nom et fonction :	Nom :	
Signature :	Signature :	

Downloaded from http://bmjopen.bmj.com/ on September 30, 2017 - Published by group.bmj.com

Open Access Protocol

BMJ Open

Evolution of pain at 3 months by oral resveratrol in knee osteoarthritis (ARTHROL): protocol for a multicentre randomised double-blind placebo-controlled trial

Christelle Nguyen,[1,2,3] Isabelle Boutron,[1,4,5] Gabriel Baron,[1,5] Emmanuel Coudeyre,[6] Francis Berenbaum,[7,8,9] Serge Poiraudeau,[1,2,10,11] François Rannou[1,2,3]

To cite: Nguyen C, Boutron I, Baron G, *et al.* Evolution of pain at 3 months by oral resveratrol in knee osteoarthritis (ARTHROL): protocol for a multicentre randomised double-blind placebo-controlled trial. *BMJ Open* 2017;**7**:e017652. doi:10.1136/bmjopen-2017-017652

► Prepublication history and additional material for this paper are available online. To view these files please visit the journal (http://dx.doi.org/10.1136/bmjopen-2017-017652).

SP deceased.

Received 5 May 2017
Revised 19 July 2017
Accepted 2 August 2017

CrossMark

For numbered affiliations see end of article.

Correspondence to
Dr Christelle Nguyen;
christelle.nguyen2@aphp.fr

ABSTRACT

Introduction Osteoarthritis (OA) pathophysiology is driven in part by joint inflammation. Resveratrol has in vitro anti-inflammatory properties. We aim to assess the efficacy of oral resveratrol for knee pain at 3 months in people with knee OA.

Methods and analysis We will conduct a randomised double-blind placebo-controlled trial. Overall, 164 individuals with knee OA fulfilling 1986 American College of Rheumatology criteria will be recruited in three tertiary care centres in France and randomised to receive oral resveratrol, 40 mg (two caplets) two times per day for 1 week, then 20 mg (one caplet) two times per day or a matching placebo for a total of 6 months. Randomisation will be centralised and stratified by centre. The allocation ratio of assignments will be 1:1. The primary outcome will be the mean change from baseline in knee pain on a self-administered 11-point pain Numeric Rating Scale at 3 months. Secondary outcomes will be the mean change in knee pain at 6 months, the function subscore of the Western Ontario and McMaster Universities Arthritis Index score, patient global assessment, proportion of responders according to the Osteoarthritis Research Society International–Outcome Measures in Rheumatology criteria at 3 and 6 months, and self-reported number of intra-articular injections of corticosteroids or hyaluronic acid and consumption of analgesics and non-steroidal anti-inflammatory drugs since the last contact. Other interventions will be allowed and self-reported. Adherence will be monitored by capsule counts and a booklet and adverse events recorded at 3 and 6 months. Statisticians, treating physicians and participants will be blinded to the allocated treatment.

Ethics and dissemination The oral resveratrol in knee osteoarthritis (ARTHROL) trial has been authorised by the *AgenceNationale de Sécurité du Médicament et des Produits de Santé* and ethics were approved by the *Comité deProtection des Personnes Île-de-France* III. The findings of the study will be published in a peer-reviewed journal and disseminated at conferences. The design of ARTHROL will warrant the translation of its findings into clinical practice.

Trial registration number ClinicalTrials.gov identifier: NCT02905799. Pre-results. First received: 14 September 2016. Last updated: 16 September 2016. Status: not yet recruiting.

Strengths and limitations of this study

- ► First randomised controlled trial to assess the effects of oral resveratrol on pain in knee osteoarthritis.
- ► A design to facilitate the translation of findings into clinical practice.
- ► Innovative new formulation of oral resveratrol to improve its bioavailability.
- ► Selection of primary and secondary efficacy outcomes in accordance with Outcome Measures in Rheumatology recommendations and Core Outcome Measures in Effectiveness Trials initiative for phase III clinical trials in knee osteoarthritis.
- ► Participants will be recruited from tertiary care centres and may not be fully representative of the population with knee osteoarthritis in France.

INTRODUCTION

In the 2015 Global Burden of Disease Study, musculoskeletal disorders were identified among the five main contributors to disability-adjusted life years.[1] Knee osteoarthritis (OA) is one of the most disabling joint disorders in Western countries[2] and OA is the first cause of disability in people over 40 years old in France.[3]

OA pathophysiology is in part driven by local joint inflammation leading to severe tissue damage. No efficient treatment exists for structural changes in OA; the only treatments are for painful symptoms and are mainly acetaminophen, non-steroidal anti-inflammatory drugs (NSAIDs) and weak opioids. Unfortunately, acetaminophen is weakly effective, with a poor effect size of 0.10, and recent data highlighted its potential cardiovascular

Downloaded from http://bmjopen.bmj.com/ on September 30, 2017 - Published by group.bmj.com

adverse effects.[4 5] For NSAIDs, serious cardiovascular and digestive side effects do not support their prescription for long duration. An optimised treatment for OA should be efficient for both pain and inflammation, with minimal adverse effects.

Resveratrol is a molecule of interest because it has in vitro and in vivo anti-inflammatory and chondroprotective properties.[6 7] Resveratrol is available over the counter in France as a dietary supplement. No serious toxicity has been reported. In the field of rheumatic diseases, in vitro evidence supports anti-inflammatory, anticatabolic, anti-apoptotic and antioxidative properties of resveratrol in various articular cell types, along with immunomodulation properties for T and B lymphocytes.[8–20] Consistently, resveratrol administered intra-articularly has shown chondroprotective effects in preclinical models of OA, mediated by decreased production of proinflammatory and prodegradative soluble factors, and modulation of cellular and humoral responses.[21–23] In clinical research, resveratrol has been evaluated in ageing, cancer, neurodegenerative diseases, menopausal conditions, and cardiovascular and liver diseases.[6] The doses used in these trials were variable and not adjusted to the low bioavailability of oral formulations.

New formulations of resveratrol have allowed for an increase in oral resveratrol bioavailability.[24] The plasmatic peak is 10-fold increased and blood concentration remains at significant levels for several hours. We hypothesised that oral resveratrol in a new formulation could reduce knee pain at 3 months as compared with placebo in people with knee OA.

METHODS AND ANALYSIS

Design overview

This is a prospective, parallel-group, double-blind, randomised controlled multicentre study. Duration of follow-up for each participant will be 6 months postrandomisation. The study will be reported according to the Consolidated Standards of Reporting Trials statement.[25]

Setting and participants

Participants will be prospectively recruited among inpatients and outpatients from Rheumatology and Rehabilitation departments of three tertiary care centres in France with expertise in OA management (Cochin and Saint-Antoine Hospitals, Paris, France and Gabriel-Montpied Hospital, Clermont-Ferrand, France), by advertising on the internet and in the media (newspapers and health magazines) and by using posters in each investigating centre. People interested in participating in the study will be invited to contact a biomedical research technician by phone or email. In addition, the computerised medical records of each investigating centre will be searched from 2015 to 2017, and patients with the key words 'knee OA' in the records will be invited to participate in the study by phone or mail by the biomedical research technician. The number of patients treated yearly for knee OA in the participating centres is approximately 2000. The biomedical research technician will check for eligibility criteria, then, if appropriate, set up a face-to-face baseline visit with one of the investigators, a senior specialist in rehabilitation and/or rheumatology. The main eligibility criteria will be knee OA fulfilling 1986 American College of Rheumatology criteria, pain on a self-administered 11-point pain Numeric Rating Scale (NRS) ≥40/100, symptom duration ≥1 month and Kellgren and Lawrence X-ray score 1, 2 or 3. A complete description of the inclusion and non-inclusion criteria is presented in online supplementary appendix 1. Patients excluded for temporary reasons can be rescreened.

Experimental group

40 mg (two caplets) of resveratrol will be administered orally two times per day, 30 min before a meal with a glass of water, for 1 week, then 20 mg (one caplet) two times per day for a total of 6 months. Pharmacokinetics, bioavailability and toxicity of *trans*-resveratrol formulation used in the oral resveratrol in knee osteoarthritis (ARTHROL) trial have been previously described in a phase I clinical trial.[24] Briefly, 15 healthy volunteers received a single dose of 40 mg of oral *trans*-resveratrol in two forms (soluble galenic formulation or dry powder). The single dose of the soluble *trans*-resveratrol was well absorbed and elicited biologically efficient blood levels (0.1–6 µM) for several hours. The soluble formulation led to 8.8-fold higher *trans*-resveratrol levels in plasma versus the powder. We have made substantial modifications to the administration scheme as compared with the one tested in the phase I clinical trial: (1) because *trans*-resveratrol is metabolised into glucuronide and sulfate conjugates coupled to renal elimination, we hypothesised that giving a loading dose for 1 week may allow attaining the drug effect more rapidly, and (2) for the maintenance dose, we chose 40 mg a day as tested in the phase I clinical trial, but in two doses, because the half-life of the soluble galenic formulation of *trans*-resveratrol is 79 min only. Resveratrol will be freely supplied by the Yvery Laboratory (patent number WO 2012/007252, Marseille, France). Resveratrol is considered a dietary supplement and is available over the counter. No marked toxicity has been reported.[26] The caplets used in this study will be exactly the same as those already available on the French market. They will be stored in their original packaging at room temperature, protected from humidity, light and excessive heat. A box containing 7 pillboxes of 60 caplets each of resveratrol will be provided to each person randomised to the experimental group. Individuals will be asked to return the pillboxes for capsule counts at the 3-month and 6-month visits and to self-report adherence by completing a booklet. However, no specific measures will be taken to enhance adherence.

Control group

The Yvery Laboratory will supply the placebo and ensure that it has a similar condition and taste as resveratrol. Two

Downloaded from http://bmjopen.bmj.com/ on September 30, 2017 - Published by group.bmj.com

caplets of placebo will be administered orally two times per day for 1 week, then one capsule two times per day for a total of 6 months. A box containing 7 pillboxes of 60 caplets each of placebo will be provided to each person randomised to the control group and stored under the same conditions as resveratrol.

Co-interventions

Pharmacological and non-pharmacological treatments usually prescribed for knee OA will be authorised. Rescue medications (analgesics and NSAIDs), joint injections (hyaluronic acid and corticosteroids), symptomatic slow acting drugs for OA (SYSADOA) and non-pharmacological co-interventions including brace, insoles, walking aids, physiotherapy, home-based therapeutic exercises and weight loss will be assessed by using a standardised checklist and recorded in the electronic case report form (eCRF).

Outcomes

Primary and secondary efficacy outcomes have been selected in accordance with Outcome Measures in Rheumatology (OMERACT)[27] and Osteoarthritis Research Society International (OARSI) recommendations[28] and the Core Outcome Measures in Effectiveness Trials initiative for phase III clinical trials of knee OA. As recommended, the outcomes include those for pain, physical function and patient global assessment. The primary efficacy outcome is the mean change from baseline in mean knee pain in the previous 48 hours on a self-administered 11-point pain NRS (0, no pain, to 100, maximal pain) at 3 months. The secondary efficacy outcomes are the mean change in mean knee pain on a pain NRS at 6 months, the mean change in the function subscore of the self-administered Western Ontario and McMaster Universities Arthritis Index (WOMAC) questionnaire at 3 and 6 months (the French version of the questionnaire),[29] the mean change in patient global assessment at 3 and 6 months on a self-administered 11-point global assessment NRS (0, worst possible, to 100, best possible), the proportion of responders according to the OARSI–OMERACT at 3 and 6 months,[30] the self-reported number of intra-articular injections of corticosteroids or hyaluronic acid and the self-reported consumption of analgesics (non-opioid, weak and strong opioids) and NSAIDs since the last contact on a self-administered four-class scale (never, several times a month, several times a week, daily) at 3 and 6 months. Information about the WOMAC questionnaire and OARSI–OMERACT response is presented in online supplementary appendix 2. For participants who discontinue or deviate from intervention protocols, the same outcome data will be collected if possible.

Randomisation and allocation concealment

Individuals who meet the inclusion criteria and agree to participate will be randomly assigned to the resveratrol or placebo group at the inclusion visit. The allocation ratio of assignments will be 1:1. Participants, care providers, data collectors, outcome assessors and statisticians will be blinded to the allocated group. The randomisation sequence will be computer generated by a statistician of the *Centre d'Épidémiologie Clinique*. The list will be stratified by centres with variable block sizes. The randomisation process will be centralised at the coordinating office (*Unité de Recherche Clinique*, Cochin Hospital), which will have no involvement in the enrolment, follow-up or assessment of participants. Only the independent statistician of the *Centre d'Épidémiologie Clinique*, the computer programmer at the coordinating office who will implement the sequence assignment in the secure eCRF, and the Yvery Company will have access to the randomisation list. The Yvery Company will label the resveratrol and placebo caplets and provide them with strictly identical presentations to each centre for the whole research duration. In each centre, the investigator will blindly deliver the medication to patients enrolled according to their randomisation number, at once, for the whole research duration. The sequence will be concealed by use of a computer interface implemented in the eCRF. Treatment administration and clinical monitoring of the experimental products will be the same in the experimental and control groups.

Blinding can be broken only if the investigator deems it necessary for the safe management of a specific medical condition of a subject, and whenever possible, the methodologist and sponsor will be consulted before breaking the blind. If the blind is broken for any reason during the study, the moment at which the blind was broken and all other relevant information will be documented by the investigative site and other sponsor designees, as appropriate. The reason for breaking the blind will be indicated and justified in the source documentation and in the eCRF.

Statistical aspects

The sample size is estimated at 164 patients. We have predicted a difference in mean change from baseline of 15 points on the pain NRS between resveratrol and placebo groups, with a SD of 27 points, and a power of 90%, corresponding to 69 patients in each arm. Considering a 15% lost to follow-up, we will need to enrol an estimated 82 patients for each arm. Fifteen points on pain NRS is considered the minimal clinically perceived difference in pain for patients with knee OA. All analyses will be performed on an intent-to-treat basis, in that all patients will be considered in the analysis and will be analysed in the group to which they had been assigned. For descriptive analyses, qualitative variables will be reported with absolute and relative frequencies and quantitative variables with median (IQR). To compare differences in changes in values between the two groups for quantitative variables, a constrained longitudinal data analysis will be used. In this model, both the baseline and postbaseline values will be modelled as dependent variables (the constrained longitudinal data analysis model assumes that both the baseline and postbaseline measurements

Downloaded from http://bmjopen.bmj.com/ on September 30, 2017 - Published by group.bmj.com

are jointly multivariate normally distributed because the baseline value is treated as part of the response vector). The true baseline means will be constrained to be the same for the two treatment groups. This analysis provides an adjustment for the observed baseline difference in estimating the treatment effects. Random effects at patient and centre levels will be added to these models. Results will be expressed as differences in mean change from baseline with 95% CI at 3 and 6 months. The constrained longitudinal data analysis model can include all randomised subjects with a baseline or postbaseline value. Such methods based on maximum likelihood are consistent under the missing-at-random assumption. Qualitative outcomes will be analysed by a mixed logistic regression model with a random effect at centre levels. Data analysis will involve use of SAS V.9.4. Blinded statisticians will perform the statistical analyses at an independent centre (*Centre d'Épidémiologie Clinique*, Paris Descartes, Hôpital Hôtel-Dieu). The statistical analysis will be further detailed in a dedicated Statistical Analysis Plan before any analysis is undertaken.

Participant timeline

Schedule of enrolment, interventions and assessments is shown in Table 1.

Baseline visit

Inclusion and non-inclusion criteria will be validated at baseline by the investigator during a face-to-face visit. The individual will be informed and the written consent collected by the investigator. Then, the participant will be enrolled and randomised. Specific additional clinical examination, laboratory tests or imaging will not be required for the purpose of the study. Information regarding demographics (age, gender, body mass index, education and employment status), medical history (date

Table 1 Schedule of enrolment, interventions and assessments

	**Study period**		
	**Enrolment**	**Postallocation**	**Close-out**
**Timepoints**	**Month 0**	**Month 3**	**Month 6**
Eligibility screen	X	–	–
Informed consent	X	–	–
Allocation	X	–	–
Delivery of the experimental product	X	–	–
Delivery of a participating card	X	–	–
Instructions to keep and return the pillboxes	X	–	–
Interventions			
Oral resveratrol			
Oral placebo			
Assessments			
Baseline variables			
Demographics	X	–	–
Medical history	X	–	–
Outcome measures (analyses planned)			
Knee pain	X	X	X
WOMAC function subscore	X	X	X
Patient global assessment	X	X	X
OARSI–OMERACT response	–	X	X
Analgesics and NSAIDs consumption	X	X	X
Injections of hyaluronic acid and/or corticosteroids	X	X	X
Collected variables (no analyses planned)			
Symptomatic slow acting drugs for OA consumption			
Non-pharmacological co-interventions	X	X	X
Adverse events	–	X	X
Adherence	–	X	X

NSAIDs, non-steroidal anti-inflammatory drugs; OA, osteoarthritis; OARSI, Osteoarthritis Research Society International; OMERACT, Outcome Measures in Rheumatology; WOMAC, Western Ontario and McMaster Universities Arthritis Index.

Downloaded from http://bmjopen.bmj.com/ on September 30, 2017 - Published by group.bmj.com

of diagnosis, symptoms duration, side affected, medical history, surgery and trauma history of the affected knee, X-ray findings including Kellgren and Lawrence grade and OA location (ie, femorotibial medial, femorotibial lateral and/or patellofemoral OA)), medications in the previous 3 months (analgesics, NSAIDs and intra-articular injections of hyaluronic acid and/or corticosteroids and SYSADOA) and current non-pharmacological co-interventions (brace, insoles, walking aids, physiotherapy, home-based therapeutic exercises and weight loss) will be recorded in the eCRF by using a standardised checklist. Baseline values for prespecified assessment criteria will be collected by using printed self-administered questionnaires and data will be entered in the eCRF by a biomedical research technician. The investigator will deliver the experimental product to the participants according to their randomisation number and give them a participating card in a clinical trial. Participants will be asked to keep and return the pillboxes for capsule counts at the 3-month and 6-month visits and to self-report adherence by completing a booklet.

Three-month and 6 month visits

The investigator will assess participants during a face-to-face visit. Values for prespecified assessment criteria will be collected at 3 and 6 months by using printed self-administered questionnaires, and data will be recorded in the eCRF by a biomedical research technician. In addition, the investigator will record adverse events (AEs) since the last contact by asking an open-ended question ("Did you have any adverse events since the last contact?"), count the caplets remaining in the pillboxes, check the self-reported adherence booklet and assess non-pharmacological co-interventions by using a standardised checklist. In the event that the participant missed the appointment, self-administered questionnaires, AEs, capsule counts and non-pharmacological co-interventions will be collected by mail, email or phone by a biomedical research technician and recorded. To reduce the amount of missing data, promote participant retention and complete the follow-up, reminder newsletters will be sent once a month by mail or email to inform participants of the progression of the study.

End of the research

At the end of the research, patients will be advised to continue their usual medical follow-up with their treating physician. Ending a subject's participation will not affect the normal medical management in any way. No exclusion period for another biomedical research will be required. At the end of the study, participants will be informed of the results on request.

Data management

Data collection

Data will be entered into an eCRF, completed by the investigator during each visit. Data from printed self-administered questionnaires will be entered in the eCRF by a biomedical research assistant after the visits. The investigator must give an explanation for each missing data. Changes in the data in the eCRF will be tracked. Discordant data in the eCRF will be corrected by queries.

Data monitoring

In accordance with the French Good Clinical Practices, the sponsor, DRCD, is responsible for obtaining the permission of all parties involved in the research to guarantee direct access to all locations where the research is carried out, the source data, the source documents and the reports, with the goal of quality control and audit by the sponsor. In accordance with the legislative and regulatory provisions in force (Articles L.1121-3 and R.5121-13 of the French Public Health Code), the investigators will make available to those in charge of monitoring, quality control and audit relating to the biomedical research the documents and personal data strictly necessary for these controls. Source documents are defined as any original document or object that can prove the existence or accuracy of a piece of information or a fact recorded during the research.

Quality control

A clinical research associate appointed by the sponsor will be responsible for the proper conduct of the research and for collecting and documenting, recording and reporting the data generated in writing, in accordance with the standard operating procedures applied within the DRCD and in accordance with French Good Clinical Practices as well as with the legislative and regulatory provisions in force. The investigator and the members of the investigator's team agree to make themselves available during quality control visits carried out at regular intervals by the clinical research associate. During these visits, the following elements will be reviewed: written consent, compliance with the research protocol and with the procedures defined therein, quality of the data collected in the eCRF including accuracy, missing data, consistency of the data with the source documents (medical files, appointment books, original copies of laboratory results, among others) and management of the treatments used.

ETHICS AND DISSEMINATION

Ethical considerations

Methods for obtaining information and consent from research participants

In accordance with Article L.1122-1-1 of the French Public Health Code, no biomedical research can be carried out on a person without free and informed consent obtained in writing after the person has been given the information specified in Article L.1122-1 of said code. The investigator or a doctor representing the investigator obtains the free and informed consent, in writing, of the individual before their inclusion in the research. The information sheet and a copy of the consent form signed and dated by the research participant and by the investigator or the doctor

Downloaded from http://bmjopen.bmj.com/ on September 30, 2017 - Published by group.bmj.com

representing the investigator are given to the individual before their participation in the research. In addition, the investigator will specify in the research participant's medical file the methods used for obtaining consent as well as the methods used for providing information with the goal of obtaining consent. The investigator will retain the original signed and dated copy of the participant's consent form.

Data confidentiality

Those responsible for biomedical research quality control (Article L.1121-3 of the French Public Health Code) will take all necessary precautions to ensure the confidentiality of information about the experimental medications, the research, the research subjects and in particular the identity of the participants and the results obtained. Investigators are subject to professional secrecy (in accordance with the conditions set out in Articles 226-13 and 226-14 of the Penal Code). During or after the biomedical research, the data collected for the research participants and sent to the sponsor by the investigators (or any other specialised parties) will be made non-identifying. Anonymisation of the patients will be ensured by using a code number and initials, reported on each needed document for the research, or by erasing nominative data on copies of source documents. Under no circumstances should the names and addresses of the subjects involved be shown. The sponsor will ensure that each research participant has given permission in writing for access to their personal information that is strictly necessary for the quality control of the research. Access to the eCRF will be restricted by an access code and a personal and unique password system for each user. Each investigator will, in addition, have access to a specific profile that attributes or withholds access to certain functions of the system (entering data, or simply viewing the data of the enrolled participant or all the study data, possibility of change and validation by the clinical research associate, etc). Data will be stored on a secure server, with data encrypted during transmission and automatic internal saving of a copy on the server that will host the eCRF. This research falls under the *Méthodologie de référence* according to the provisions of Article 54, paragraph 5 of modified Law no 78-17 of 6 January 1978 relating to information technology, data files and privacy. This change was approved in a decision on 5 January 2006. AP-HP, the research sponsor, has signed a commitment to comply with the *Méthodologie de référence*. Specific documents for biomedical research will be archived by the investigator and the sponsor for 15 years after the end of the research.

Legal obligations

AP-HP is the sponsor of this research and by delegation, the DRCD performs the research's missions in accordance with Article L.1121-1 of the French Public Health Code. For this biomedical research relating to a medication for human use and prior to starting the research, AP-HP has obtained the favourable opinion of the *Comité de Protection des Personnes (CPP) Île-de-France* III, within the scope of its authority and in accordance with the legislative and regulatory provisions in force. AP-HP has also obtained authorisation from the *Agence Nationale de Sécurité du Médicament et des produits de santé* (ANSM) (French Health Products Safety Agency); registration number RCB 2016-A01310-51). AP-HP has signed a commitment to comply with the *Méthodologie de reference*. AP-HP will make a standard declaration to the *Commission Nationale de l'Informatique et des Libertés*.

Modifications to the research

Any substantial modification to the protocol by the coordinating investigator must be sent to the sponsor for approval. After approval is given, the sponsor must obtain, before starting the research, a favourable opinion from the CPP and authorisation from the ANSM within the scope of their respective authorities. The information sheet and the consent form can be revised if necessary, particularly with substantial modification to the research or if adverse reactions occur.

Safety considerations

The investigator will record all serious AEs (SAEs) and non-SAEs since the last contact by asking an open-ended question ("Did you have any adverse events since last contact?") during face-to-face visits at 3 and 6 months. In the event that the participant missed the appointment, AEs will be requested by mail, email or phone by a biomedical research technician and recorded. All SAEs and non-SAEs will be recorded in the 'Adverse events' section of the eCRF by the investigator.

Notification of an SAE

The investigator will notify the sponsor, immediately on the day when he/she becomes aware, of any SAE, except those that are prespecified (see below). The investigator must report all SAEs that occur in research participants on the date of the first administration of an investigational product and throughout the period when the participant is monitored. SAEs that do not require immediate notification to the sponsor are recorded in the 'Adverse events' section of the eCRF. They include events associated with (1) the normal and natural evolution of the pathology including scheduled medical visits for the follow-up of knee OA, scheduled hospitalisations for the routine treatment of knee OA (joint injection and rehabilitation), and not related to a worsening of the condition, and expected symptoms secondary to knee OA worsening such as joint pain, joint effusion, OA flare, walking difficulties or surgical knee joint replacement for OA; (2) special circumstances including hospitalisations for pre-existing conditions, surgery scheduled prior to the research, social or administrative purposes or admission to the emergency room less than 12 hours and (3) AEs likely to be associated with the treatments prescribed as part of the patient's care during the monitoring of the research, including AEs related to rescue medications

Downloaded from http://bmjopen.bmj.com/ on September 30, 2017 - Published by group.bmj.com

(analgesics and NSAIDs) or joint injections (hyaluronic and corticosteroids) that include increased pain, joint swelling, mild joint effusion that can last a few days, skin flush following corticosteroid injections that can last a few hours and exceptionally, septic arthritis or allergic reaction.

Investigation of an SAE

The investigator will document the SAE as thoroughly as possible and provide the medical diagnosis by using a specific SAE form. The investigator will assess the severity of the SAE: (1) mild, tolerated by the participant, does not interfere with daily activities; (2) moderate, sufficiently uncomfortable to affect daily activities and (3) serious, preventing daily activities. The investigator will assess the causality relation between the SAE and the clinical trial. The method the investigator uses will be based on WHO–Uppsala Monitoring Centre method and will include the following four causality terms: (1) certain, (2) probable/likely, (3) possible and (4) unlikely (not excluded). Their comprehensive definition is provided in online supplementary appendix 3. The sponsor represented by the Vigilance department will continuously assess the safety of the clinical trial throughout the trial. The sponsor is responsible for assessing (1) the seriousness of all AEs reported and (2) the causality relation between the SAE and the acts/procedures/tests added by the clinical trial.

Investigation of an AE

All SAEs considered by the investigator and/or the sponsor to be possibly related to the act/procedures/tests/products administered, specific to the clinical trial, can be reasonably considered suspected adverse reactions (SARs). Any SAR whose nature, severity or outcome is not consistent with the information related to the acts/procedures/and or products administered during the clinical study is considered unexpected. The sponsor represented by the Vigilance department will assess the expected/unexpected nature of an SAR according to the information described in online supplementary appendix 4.[26] The sponsor reports any suspected unexpected serious adverse reaction, within the legal deadline, to the ANSM and CPP.

Dissemination plan

We aim to publish the results of ARTHROL trial in a peer-reviewed journal and present the findings to physicians who manage knee OA at national and international conferences. The investigators will be involved in drafting manuscripts, abstracts, press releases and any other publications arising from the trial. Authorship will be determined in accordance with the International Committee of Medical Journal Editors guidelines. There will be no intended use of professional writers. AP-HP is the owner of the data, which cannot be used or disclosed to a third party without prior approval from the AP-HP. The full original protocol in English and the full dataset will be available by contacting the coordinating investigator, Prof François Rannou (francois.rannou@aphp.fr). Statistical codes will be available by contacting the biostatistician of the study, Dr Gabriel Baron (gabriel.baron@aphp.fr).

The ARTHROL study will be the first to assess the clinical effects of oral resveratrol in knee OA. If the results are positive, resveratrol will represent an interesting and safe alternative for treating painful knee OA.

Author affiliations
[1]Faculté de Médecine, Sorbonne Paris Cité, Université Paris Descartes, Paris, France
[2]Service de Rééducation et de Réadaptation de l'Appareil Locomoteur et des Pathologies du Rachis, AP-HP, Hôpitaux Universitaires Paris Centre-Groupe Hospitalier Cochin, Paris, France
[3]INSERM UMR 1124, Laboratoire de Pharmacologie, Toxicologie et Signalisation Cellulaire, Faculté des Sciences Fondamentales et Biomédicales, Centre Universitaire des Saints-Pères, Paris, France
[4]AP-HP, Hôpital Hôtel-Dieu, Centre d'Épidémiologie Clinique, Paris, France
[5]INSERM UMR 1153, Centre de Recherche Épidémiologie et Statistique, Sorbonne Paris Cité, METHODS Team, Paris, France
[6]Service de Médecine Physique et de Réadaptation, Centre Hospitalo-Universitaire de Clermont-Ferrand, INRA, Université Clermont Auvergne, Clermont-Ferrand, France
[7]Université Paris Pierre et Marie-Curie, Sorbonne Paris Cité, Paris, France
[8]Service de Rhumatologie, AP-HP, Hôpital Saint-Antoine, Paris, France
[9]INSERM UMR 938, DHU i2B, Paris, France
[10]INSERM UMR 1153, Centre de Recherche Épidémiologie et Statistique, Sorbonne Paris Cité, ECaMO Team, Paris, France
[11]Institut Fédératif de Recherche sur le Handicap, Paris, France

Acknowledgements The authors thank Alexandra Bruneau from URC-CIC Paris Descartes Necker/Cochin for implementation, monitoring and data management and Laura Smales for professional copyediting.

Contributors Conception and design of the study: CN, IB, GB, EC, FB, SP and FR. Drafting of the original protocol: CN, IB, GB, SP and FR. Coordination of the study: CN and FR. Design of the statistical analysis plan: IB and GB. Drafting of the present manuscript: CN and IB. Final approval: CN, IB, GB, EC, FB and FR.

Funding This work was supported by the French Ministry of Health (Programme Hospitalier de Recherche Clinique 2015, project no 15-15-0234) and sponsored by the Département de la Recherche Clinique et du Développement (DRCD) of the AssistancePublique-Hôpitaux de Paris (AP-HP). The Yvery Laboratory will supply the resveratrol and the placebo. The funding source and the Yvery Laboratory will not be involved in the study design; collection, management, analysis and interpretation of data; or writing of the report; nor decision to publish the results.

Competing interests None declared.

Ethics approval Comité de Protection des Personnes Île-de-France III.

Provenance and peer review Not commissioned; externally peer reviewed.

Open Access This is an Open Access article distributed in accordance with the Creative Commons Attribution Non Commercial (CC BY-NC 4.0) license, which permits others to distribute, remix, adapt, build upon this work non-commercially, and license their derivative works on different terms, provided the original work is properly cited and the use is non-commercial. See: http://creativecommons.org/licenses/by-nc/4.0/

© Article author(s) (or their employer(s) unless otherwise stated in the text of the article) 2017. All rights reserved. No commercial use is permitted unless otherwise expressly granted.

REFERENCES

1. Disease GBD, Injury I, Global PC.GBD 2015 Disease and Injury Incidence and Prevalence Collaborators. Global, regional, and national incidence, prevalence, and years lived with disability for 310 diseases and injuries, 1990-2015: a systematic analysis for the Global Burden of Disease Study 2015. *Lancet* 2016;388:1545–602.
2. Bijlsma JW, Berenbaum F, Lafeber FP. Osteoarthritis: an update with relevance for clinical practice. *Lancet* 2011;377:2115–26.

Downloaded from http://bmjopen.bmj.com/ on September 30, 2017 - Published by group.bmj.com

3. Palazzo C, Ravaud JF, Papelard A, *et al*. The burden of musculoskeletal conditions. *PLoS One* 2014;9:e90633.
4. Richette P, Latourte A, Frazier A. Safety and efficacy of paracetamol and NSAIDs in osteoarthritis: which drug to recommend? *Expert Opin Drug Saf* 2015;14:1259–68.
5. Roberts E, Delgado Nunes V, Buckner S, *et al*. Paracetamol: not as safe as we thought? A systematic literature review of observational studies. *Ann Rheum Dis* 2016;75.
6. Vang O, Ahmad N, Baile CA, *et al*. What is new for an old molecule? Systematic review and recommendations on the use of resveratrol. *PLoS One* 2011;6:e19881.
7. Nguyen C, Savouret JF, Widerak M, *et al*. Resveratrol, potential therapeutic interest in joint disorders: a critical narrative Review. *Nutrients* 2017;9:45.
8. Dave M, Attur M, Palmer G, *et al*. The antioxidant resveratrol protects against chondrocyte apoptosis via effects on mitochondrial polarization and ATP production. *Arthritis Rheum* 2008;58:2786–97.
9. Li X, Phillips FM, An HS, *et al*. The action of resveratrol, a phytoestrogen found in grapes, on the intervertebral disc. *Spine* 2008;33:2586–95.
10. Malemud CJ. Inhibitors of stress-activated protein/mitogen-activated protein kinase pathways. *Curr Opin Pharmacol* 2007;7:339–43.
11. Mengshol JA, Vincenti MP, Coon CI, *et al*. Interleukin-1 induction of collagenase 3 (matrix metalloproteinase 13) gene expression in chondrocytes requires p38, c-Jun N-terminal kinase, and nuclear factor kappaB: differential regulation of collagenase 1 and collagenase 3. *Arthritis Rheum* 2000;43:801–11.
12. Shakibaei M, Mobasheri A, Buhrmann C. Curcumin synergizes with resveratrol to stimulate the MAPK signaling pathway in human articular chondrocytes in vitro. *Genes Nutr* 2011;6:171–9.
13. Csaki C, Mobasheri A, Shakibaei M. Synergistic chondroprotective effects of curcumin and resveratrol in human articular chondrocytes: inhibition of IL-1beta-induced NF-kappaB-mediated inflammation and apoptosis. *Arthritis Res Ther* 2009;11:R165.
14. Lei M, Liu SQ, Liu YL. Resveratrol protects bone marrow mesenchymal stem cell derived chondrocytes cultured on chitosan-gelatin scaffolds from the inhibitory effect of interleukin-1beta. *Acta Pharmacol Sin* 2008;29:1350–6.
15. Liu FC, Hung LF, Wu WL, *et al*. Chondroprotective effects and mechanisms of resveratrol in advanced glycation end products-stimulated chondrocytes. *Arthritis Res Ther* 2010;12:R167.
16. Shakibaei M, Csaki C, Nebrich S, *et al*. Resveratrol suppresses interleukin-1beta-induced inflammatory signaling and apoptosis in human articular chondrocytes: potential for use as a novel nutraceutical for the treatment of osteoarthritis. *Biochem Pharmacol* 2008;76:1426–39.
17. Tian J, Chen JW, Gao JS, *et al*. Resveratrol inhibits TNF-α-induced IL-1β, MMP-3 production in human rheumatoid arthritis fibroblast-like synoviocytes via modulation of PI3kinase/Akt pathway. *Rheumatol Int* 2013;33:1829–35.
18. Byun HS, Song JK, Kim YR, *et al*. Caspase-8 has an essential role in resveratrol-induced apoptosis of rheumatoid fibroblast-like synoviocytes. *Rheumatology* 2008;47:301–8.
19. Ferrero ME, Bertelli AE, Fulgenzi A, *et al*. Activity in vitro of resveratrol on granulocyte and monocyte adhesion to endothelium. *Am J Clin Nutr* 1998;68:1208–14.
20. Vorderstrasse BA, Steppan LB, Silverstone AE, *et al*. Aryl hydrocarbon receptor-deficient mice generate normal immune responses to model antigens and are resistant to TCDD-induced immune suppression. *Toxicol Appl Pharmacol* 2001;171:157–64.
21. Elmali N, Esenkaya I, Harma A, *et al*. Effect of resveratrol in experimental osteoarthritis in rabbits. *Inflamm Res* 2005;54:158–62.
22. Wang J, Gao JS, Chen JW, *et al*. Effect of resveratrol on cartilage protection and apoptosis inhibition in experimental osteoarthritis of rabbit. *Rheumatol Int* 2012;32:1541–8.
23. Li W, Cai L, Zhang Y, *et al*. Intra-articular resveratrol injection prevents osteoarthritis progression in a mouse model by activating SIRT1 and thereby silencing HIF-2α. *J Orthop Res* 2015;33:1061–70.
24. Amiot MJ, Romier B, Dao TM, *et al*. Optimization of trans-Resveratrol bioavailability for human therapy. *Biochimie* 2013;95:1233–8.
25. Schulz KF, Altman DG, Moher D, *et al*. CONSORT 2010 statement: updated guidelines for reporting parallel group randomised trials. *BMJ* 2010;340:c332.
26. Cottart CH, Nivet-Antoine V, Laguillier-Morizot C, *et al*. Resveratrol bioavailability and toxicity in humans. *Mol Nutr Food Res* 2010;54:7–16.
27. Bellamy N, Kirwan J, Boers M, *et al*. Recommendations for a core set of outcome measures for future phase III clinical trials in knee, hip, and hand osteoarthritis. Consensus development at OMERACT III. *J Rheumatol* 1997;24:799–802.
28. McAlindon TE, Driban JB, Henrotin Y, *et al*. OARSI Clinical Trials Recommendations: Design, conduct, and reporting of clinical trials for knee osteoarthritis. *Osteoarthritis Cartilage* 2015;23:747–60.
29. Bellamy N, Buchanan WW, Goldsmith CH, *et al*. Validation study of WOMAC: a health status instrument for measuring clinically important patient relevant outcomes to antirheumatic drug therapy in patients with osteoarthritis of the hip or knee. *J Rheumatol* 1988;15:1833–40.
30. Pham T, van der Heijde D, Altman RD, *et al*. OMERACT-OARSI initiative: Osteoarthritis Research Society International set of responder criteria for osteoarthritis clinical trials revisited. *Osteoarthritis Cartilage* 2004;12:389–99.

Downloaded from http://bmjopen.bmj.com/ on September 30, 2017 - Published by group.bmj.com

BMJ Open

Evolution of pain at 3 months by oral resveratrol in knee osteoarthritis (ARTHROL): protocol for a multicentre randomised double-blind placebo-controlled trial

Christelle Nguyen, Isabelle Boutron, Gabriel Baron, Emmanuel Coudeyre, Francis Berenbaum, Serge Poiraudeau and François Rannou

BMJ Open 2017 7:
doi: 10.1136/bmjopen-2017-017652

Updated information and services can be found at:
http://bmjopen.bmj.com/content/7/9/e017652

These include:

References This article cites 29 articles, 2 of which you can access for free at:
http://bmjopen.bmj.com/content/7/9/e017652#BIBL

Open Access This is an Open Access article distributed in accordance with the Creative Commons Attribution Non Commercial (CC BY-NC 4.0) license, which permits others to distribute, remix, adapt, build upon this work non-commercially, and license their derivative works on different terms, provided the original work is properly cited and the use is non-commercial. See: http://creativecommons.org/licenses/by-nc/4.0/

Email alerting service Receive free email alerts when new articles cite this article. Sign up in the box at the top right corner of the online article.

Notes

To request permissions go to:
http://group.bmj.com/group/rights-licensing/permissions

To order reprints go to:
http://journals.bmj.com/cgi/reprintform

To subscribe to BMJ go to:
http://group.bmj.com/subscribe/
