# Supplementary material for: Oral resveratrol in adults with knee osteoarthritis: A randomized placebo-controlled trial (ARTHROL)
Source: PLoS Med. 2024 Aug 13;21(8):e1004440. doi: 10.1371/journal.pmed.1004440 (PMC11321588; doi:10.1371/journal.pmed.1004440)
Supplement: S2 Method — (PDF) [file pmed.1004440.s008.pdf]

EVOLUTION OF PAIN AT THREE MONTHS BY ORAL RESVERATROL IN PRIMARY KNEE OSTEOARTHRITIS : A MULTICENTER, DOUBLE-BLIND, RANDOMIZED, PLACEBO-CONTROLLED TRIAL

ACRONYM: ARTHROL

Statistical analysis plan

Réf projet: **P150938 /** ANSM: N° **ID RCB : 2016-A01310-51**

Trial registration number ClinicalTrials. gov identifier: NCT02905799.

**Date**	**Version**	**Authors**	**Comments**
03/10/2022	V1	Gabriel Baron-Isabelle Boutron	First version

Coordinating Investigator / Scientific Director
Prof. François RANNOU, M.D., Ph.D.
Service de Rééducation et de Réadaptation de l'Appareil Locomoteur et des Pathologies du Rachis
Hôpitaux Universitaires Paris Centre - Groupe Hospitalier Cochin
Assistance Publique – Hôpitaux de Paris
27, Rue du Faubourg Saint-Jacques, 75014 Paris, FRANCE
Tel +33 1 58 41 25 35
Fax +33 1 58 41 25 45
francois.rannou@aphp.fr

Dr. Christelle NGUYEN, M.D., Ph.D.
Service de Rééducation et de Réadaptation de l'Appareil Locomoteur et des Pathologies du Rachis
Hôpitaux Universitaires Paris Centre - Groupe Hospitalier Cochin
Assistance Publique – Hôpitaux de Paris
27, Rue du Faubourg Saint-Jacques, 75014 Paris, FRANCE
Tel +33 1 58 41 29 45
Fax +33 1 58 41 28 35
christelle.nguyen2@aphp.fr

Methodologist
Prof. Isabelle Boutron
Centre d'Épidémiologie Clinique
Centre de Recherche Épidémiologie et Statistique Sorbonne Paris Cité (CRESS-UMR1153)
INSERM / Université Paris Descartes
Hôpital Hôtel-Dieu, 1 Place du Parvis Notre-Dame, 75004 Paris, FRANCE
Tel :+33 1 42 34 78 33
isabelle.boutron@.aphp.fr

Sponsor
Assistance Publique - Hôpitaux de Paris (AP-HP)
By delegation: Clinical Research and Innovation Department (DRCI)
Hôpital Saint-Louis
1, Avenue Claude Vellefaux, 75010 Paris, France
Chef de Projet : Tiffany MARTIN
Tel + 33 1 44 84 17 92
Fax +33 1 44 84 17 01
tiffany.martin@aphp.fr

Entity responsible for monitoring research
Unité de Recherche Clinique (URC) Paris Descartes Necker Cochin
GH Cochin – Hôpital Tarnier
89 rue d'Assas
75006 PARIS
Chef de Projet: Laetitia PEAUDECERF
Tel +33 1 58 41 12 13
Fax + 33 1 58 41 11 83
laetitia.peaudecerf@aphp.fr

Entity responsible for investigational products circuit :
Département Essais cliniques de l'AGEPS
Pharmacist: Dr Blandine Lehmann
Chef de projets : Kevin CARDET
Tel: +33 1 46 69 14 02

CONTENTS

1 Summary of the protocol 4

2 Analysis population 8

2.1 Flow diagram 8

2.2 Definition of the analysis population 8

2.3 Sample size 8

3 Outcomes 9

4 Statistical analysis 10

1 Summary of the protocol

Title	Evolution of pain at three months by oral resveratrol in primary knee osteoarthritis: a multicenter, double-blind, randomized, placebo-controlled trial
Acronym	ARTHROL
Coordinating Investigator<br><br><br><br><br><br><br><br><br><br><br>Scientific Director	Prof. François RANNOU, M.D., Ph.D.<br>Service de Rééducation et de Réadaptation de l'Appareil Locomoteur et des Pathologies du Rachis<br>Hôpitaux Universitaires Paris Centre - Groupe Hospitalier Cochin<br>Assistance Publique – Hôpitaux de Paris<br>27, Rue du Faubourg Saint-Jacques, 75014 Paris, FRANCE<br>Tel +33 1 58 41 25 35<br>Fax +33 1 58 41 25 45<br>francois.rannou@aphp.fr<br><br>Dr. Christelle NGUYEN, M.D., Ph.D.<br>Service de Rééducation et de Réadaptation de l'Appareil Locomoteur et des Pathologies du Rachis<br>Hôpitaux Universitaires Paris Centre, Groupe Hospitalier Cochin<br>Assistance Publique – Hôpitaux de Paris<br>27, Rue du Faubourg Saint-Jacques, 75014 Paris, FRANCE<br>Tel +33 1 58 41 29 45<br>Fax +33 1 58 41 28 35<br>christelle.nguyen2@aphp.fr
Sponsor	Assistance Publique - Hôpitaux de Paris
Scientific justification	OA is the first cause of handicap in individuals over 40 years-old in France. OA physiopathology is driven by local joint inflammation responsible for pain and joint destruction. Experimental studies have shown that resveratrol could modulate pain and inflammation. We hypothesize that resveratrol, in a new formulation developed by the coordinating investigator and his colleagues (INSERM U1124), improving its bioavailability, will decrease pain in patients presenting with primary knee OA.

Primary objective and assessment criterion	The primary objective of the study is to assess the efficacy on mean knee pain in the previous 48 hrs of oral resveratrol compared to placebo in patients with knee OA at 3 months.<br><br>The assessment criterion is the mean change from baseline in mean knee pain in the previous 48 hours on a self-administered 11-point pain numeric rating scale (NRS, 0 no pain - 100 maximal pain) at 3 months
Secondary objectives and assessment criteria	The secondary objectives of the study are to assess the efficacy of oral resveratrol compared to placebo in patients with knee OA mean knee pain in the previous 48 hrs at 6 months, and function, patient's global assessment, response to treatment and medication (intra-articular injections of corticosteroids or hyaluronic acid, analgesics and non-steroidal anti-inflammatory drugs [NSAIDs]) sparing effect at 3 and 6 months. Assessment criteria are:<br><br>• the mean change from baseline in mean knee pain in the previous 48 hrs on a self-administered 11-point pain NRS at 6 months<br>• the mean change from baseline in the function subscore of the self-administered Western Ontario and McMaster Universities Arthritis Index (WOMAC) questionnaire at 3 and 6 monthsthe mean change from baseline in patient's global assessment at 3 and 6 months on a self-administered 11-point global assessment NRS (0 worst possible - 100 best possible)<br>• the percentage of Osteoarthritis Research Society International (OARSI) - Outcome Measures in Rheumatology (OMERACT) responders at 3 and 6 months<br>• self-reported number of intra-articular injections of corticosteroids or hyaluronic acid since last contact at 3 and 6 months<br>Self-reported consumption of analgesics (non-opioid, weak and strong opioids) since last contact using a self-administered 4-class scale (never; several times a month; several times a week; daily) at 3 and 6 months<br><br>• self-reported consumption of NSAIDs since last contact using a self-administered 4-class scale at 3 and 6 months
Experimental design	Multicenter, double-blind, randomized, placebo-controlled trial
Population involved	Patients presenting with symptomatic primary knee OA, fulfilling 1986 American College of Rheumatology (ACR) classification criteria
• Inclusion criteria	• Age ≥ 40 years-old<br>• Knee OA fulfilling 1986 ACR criteria<br>• Pain on an 11-point NRS ≥ 40/100<br>• Symptom duration ≥ 1 month<br>• 4 > Kellgren and Lawrence X-Ray score ≥ 1<br>• Written consent obtained<br>• Health insurance cover<br>• Patients excluded for temporary reasons can be rescreened.

• Non-inclusion criteria	• History of symptomatic crystal or inflammatory arthritis<br>• Knee surgery ≤ 1 year<br>• Knee trauma ≤ 2 months<br>• Knee intra-articular injections ≤ 2 months<br>• Current use of intramuscular, intravenous or oral corticosteroids<br>• Current use of anticoagulants<br>• Uncontrolled diseases that may require intramuscular, intravenous or oral corticosteroids<br>• Neurologic disorders involving the lower limbs<br>• Inability to speak, write or read French language<br>• Participation to another biomedical research<br>• Contraindication to resveratrol or hypersensitivity to any of its constituents
Investigational product	Resveratrol is a dietary supplement, not a drug. It will be administered orally 2 caplets twice a day for one week then one capsule twice a day for a total duration of 6 months. Resveratrol will be supplied by the industrial partner. The caplets of resveratrol have already been distributed on the French market for several years and the caplets used in this study will be exactly the same as those already available on the French market.
Control group	Placebo of resveratrol will be supplied by the industrial partner. It will present with similar conditioning and taste, and will be administered orally 2 caplets twice a day for one week then one capsule twice a day for a total duration of 6 months. The placebo use in this study will be exactly the same as the one used in a previous PHRC already accepted in neurology (Assistance Publique-Hôpitaux de Paris).
Other procedures added by the research	Not applicable
Risks added by the research	Risk A
Practical procedure	• Recruitment of eligible patients by advertisement and among in- and outpatients of the investigating centers<br>• Day 0: inclusion visit, informed consent, inclusion and randomization<br>• Month 3: follow-up visit<br>• Month 6: follow-up visit and end of the research
Number of subjects chosen	164
Number of centres	3 tertiary care centers located in France<br>• Cochin Hospital, Paris<br>• Saint-Antoine Hospital, Paris<br>• Clermont-Ferrand Hospital
Research period	• Duration of participation for each patient: 6 months<br>• Duration of recruitment: 48 months<br>• Total duration: 54 months

Number of inclusions expected per centre and per month	1.1 patients per centre and per month
Statistical analysis	Analysis will be performed according to the intention-to-treat principle by which each participant will be analyzed in his randomization arm, following a prespecified statistical analytic plan
Funding source	Assistance Publique-Hôpitaux de Paris - Programme Hospitalier de Recherche Clinique en 2015
Data Safety Monitoring Board anticipated	No

2 Analysis population

2.1 Flow diagram

At the final analysis of trial, a flow chart will be constructed according to the CONSORT 2010 reporting guidelines. It will describe:

- The number of eligible patients, randomized patients and the number of patients who have actually followed the study;
- The intervention arm allocated per randomization;
- Early cessation of the intervention and their causes and drop-outs;
- The number of patients excluded from the analysis.

The number of randomized but ineligible patients, if any, will also be reported, as well as the reason for ineligibility.

2.2 Definition of the analysis population

For interim monitoring, the analysis will be carried out according to the intention to treat (ITT) principle, i.e. each randomised participant will be analysed in the group assigned to him/her by randomisation, regardless of the actual treatment received or other protocol deviations. In particular patients randomised while not meeting eligibility criteria will be kept in the analysis.

2.3 Sample size

The sample size was estimated at 164 patients. We have predicted a difference in mean change from baseline of 15 points on the pain NRS between resveratrol and placebo groups, with a SD of 27 points, and a power of 90%, corresponding to 69 patients in each arm. Considering a 15% lost to follow-up, we needed to enrol an estimated 82 patients for each arm. Fifteen points on pain NRS is considered the minimal clinically perceived difference in pain for patients with knee OA.

3 Outcomes

The primary efficacy outcome is the mean change from baseline in mean knee pain in the previous 48hours on a self-administered 11-point pain NRS (0, no pain, to 100, maximal pain) at 3 months.

The secondary efficacy outcomes are:

- the mean change in mean knee pain on a pain NRS at 6 months,
- the mean change in the function subscore of the self-administered Western Ontario and McMaster Universities Arthritis Index (WOMAC) questionnaire at 3 and 6 months (the French version of the questionnaire)
- the mean change in patient global assessment at 3 and 6 months on a self-administered 11-point global assessment NRS (0, worst possible, to 100, best possible),
- the proportion of responders according to the OARSI–OMERACT at 3 and 6 months. Response to treatment will be defined as an improvement in pain (assessed by an 11-point pain NRS) or in function (assessed by the WOMAC function subscore) ≥ 50% and absolute change ≥ 20, or improvement in at least 2 of the 3 following: 1/ pain ≥ 20% and absolute change ≥ 10, 2/ function ≥ 20% and absolute change ≥ 10, 3/ patient's global assessment (assessed by an 11-point global assessment NRS) ≥ 20% and absolute change ≥ 10.
- the self-reported number of intra-articular injections of corticosteroids or hyaluronic acid since the last contact at 3 and 6 months.
- the self-reported consumption of analgesics (non-opioid, weak and strong opioids) since the last contact on a self-administered four-class scale (never, several times a month, several times a week, daily) at 3 and 6 months.
- the self-reported consumption of NSAIDs since the last contact on a self-administered four-class scale (never, several times a month, several times a week, daily) at 3 and 6 months.

4 Statistical analysis

The profile of selected patients and their effective follow-up through the course of the trial will be carried out in accordance with the CONSORT statement, according to a patient flow diagram. Subjects withdrawing from the study early and the reason for this will also undergo a descriptive analysis by group and for the total population. The patient follow-up parameters will be analysed for each treatment group and for the total population:

- Total follow-up duration;
- Treatment duration;
- Number of visits;
- Treatment compliance.

For each group, and at each of the evaluation dates, the qualitative endpoints will be described by their sample size, percentage and data missing by response method, and the quantitative endpoints will be described by their sample size, mean, standard deviation, median and interquartile range (25th percentile - 75th percentile), as well as the minimum and maximum.

The analysis population will involve all patients randomised into their randomisation group except:

1) Patients enrolled by mistake;
2) Patients withdrawing their informed consent and permission to use their data;
3) Patients not having given their consent.

A randomised patient who has not undergone the intervention or has partially undergone it will still be analysed. A description by group of these protocol deviations will be provided.

The primary endpoint is the change in the pain NRS score between the baseline and 3 months. The variable to be studied will therefore be the difference in Pain NRS score between randomisation (D0) and the Month 3 visit: Δ = value at M3 – value at D0. The other differences will also be calculated (between the Month 6 visit and the enrolment visit). Comparison of the differences in Δ between the groups will be studied with a CLDA (Constrained Longitudinal Data Analysis) linear model, taking into account the correlation of repeated measurements in the same subject (random patient effect with an unstructured variance-covariance matrix) under the hypothesis of randomly missing data. In this model, the baseline values are included in the response vector with the only condition that the average of

this baseline must be the same for each group, which is the case in our studies as the subjects are randomly distributed. There is therefore no reason, in theory, to believe that one group would have a higher baseline than another. The CLDA technique is consistent with the intention-to-treat principle provided that all patients have at least one initial value for the endpoint or a post-baseline value, which allows all eligible randomised patients to be included. The results will be expressed in the form of the difference between the average changes from baseline in each of the groups at 3 and 6 months with a 95% confidence interval and p-value of the associated test. Random effects at the patient level and fixed effects at the center level were added to these models. The following criteria will be analysed in the same way as the quantitative primary endpoint: WOMAC function subscore at 3 and 6 months, patient global assessment at 3 and 6 months and the self-reported number of intra-articular injections of corticosteroids or hyaluronic acid at 3 and 6 months (for this variable, a negative binomial regression model will be considered if the distribution of the variable requires it. The results will be presented in the form of mean ratio (with a 95% confidence interval) in this case).

For the proportion of responders according to the OARSI–OMERACT at 3 and 6 months, a Poisson model under GEE framework (adjustment for center will be applied) with log link allows to estimate the percentage differences (with a 95% confidence interval and p-value for the associated test) at 3 and 12 months (as well as the relative risk).

The same model will be used for the self-reported consumption of analgesics (non-opioid, weak and strong opioids) at 3 and 6 months and the self-reported consumption of NSAID at 3 and 6 months after dichotomization (never vs several times a month or several times a week or daily).

The frequency of adverse events (serious, non-serious or both) will be described in each of the 2 groups (no statistical tests are planned).

All the tests will be bilateral and at the 5% threshold. The confidence intervals will be calculated at 95%. The CLDA model (MIXED procedure) will be carried out using SAS 9.4 software (SAS Institute). The other analyses will be carried out using R 3.2.2 software (R Foundation for Statistical Computing). The glm function will be used for the Poisson model and to estimate the confidence interval of the difference between the proportions. The glm.nb 27 and coef.test functions will be used for negative binomial regression (with robust estimation of variance). Other software or other functions may be used if necessary. Blinded statisticians will perform the statistical analyses at an independent centre (Centre d'Épidémiologie Clinique, Paris Descartes, Hôpital Hôtel-Dieu).

EVOLUTION OF PAIN AT THREE MONTHS BY ORAL RESVERATROL IN PRIMARY KNEE OSTEOARTHRITIS : A MULTICENTER, DOUBLE-BLIND, RANDOMIZED, PLACEBO-CONTROLLED TRIAL

ACRONYM: ARTHROL

Statistical analysis plan

Réf projet: **P150938 /** ANSM: N° **ID RCB : 2016-A01310-51**

Trial registration number ClinicalTrials. gov identifier: NCT02905799.

**Date**	**Version**	**Authors**	**Comments**
03/10/2022	V1	Gabriel Baron-Isabelle Boutron	First version
04/10/2022	V2	Gabriel Baron-Isabelle Boutron	Second version

Coordinating Investigator / Scientific Director

Prof. François RANNOU, M.D., Ph.D.
Service de Rééducation et de Réadaptation de l’Appareil Locomoteur et des Pathologies du Rachis
Hôpitaux Universitaires Paris Centre - Groupe Hospitalier Cochin
Assistance Publique – Hôpitaux de Paris
27, Rue du Faubourg Saint-Jacques, 75014 Paris, FRANCE
Tel +33 1 58 41 25 35
Fax +33 1 58 41 25 45
francois.rannou@aphp.fr

Prof. Christelle NGUYEN, M.D., Ph.D.
Service de Rééducation et de Réadaptation de l’Appareil Locomoteur et des Pathologies du Rachis
Hôpitaux Universitaires Paris Centre - Groupe Hospitalier Cochin
Assistance Publique – Hôpitaux de Paris
27, Rue du Faubourg Saint-Jacques, 75014 Paris, FRANCE
Tel +33 1 58 41 29 45
Fax +33 1 58 41 28 35
christelle.nguyen2@aphp.fr

Methodologist

Prof. Isabelle Boutron
Centre d'Épidémiologie Clinique
Centre de Recherche Épidémiologie et Statistique Sorbonne Paris Cité (CRESS-UMR1153)
INSERM / Université Paris Descartes
Hôpital Hôtel-Dieu, 1 Place du Parvis Notre-Dame, 75004 Paris, FRANCE
Tel :+33 1 42 34 78 33
isabelle.boutron@.aphp.fr

Sponsor

Assistance Publique - Hôpitaux de Paris (AP-HP)
By delegation: Clinical Research and Innovation Department (DRCI)
Hôpital Saint-Louis
1, Avenue Claude Vellefaux, 75010 Paris, France
Chef de Projet : Tiffany MARTIN
Tel + 33 1 44 84 17 92
Fax +33 1 44 84 17 01
tiffany.martin@aphp.fr

Entity responsible for monitoring research

Unité de Recherche Clinique (URC) Paris Descartes Necker Cochin
GH Cochin – Hôpital Tarnier
89 rue d’Assas
75006 PARIS
Chef de Projet: Laetitia PEAUDECERF
Tel +33 1 58 41 12 13
Fax + 33 1 58 41 11 83
laetitia.peaudecerf@aphp.fr

Entity responsible for investigational products circuit :

Département Essais cliniques de l’AGEPS
Pharmacist: Dr Blandine Lehmann
Chef de projets : Kevin CARDET
Tel: +33 1 46 69 14 02

CONTENTS

1 Summary of the protocol 4

2 Analysis population 8

2.1 Flow diagram 8

2.2 Definition of the analysis population 8

2.3 Sample size 8

3 Outcomes 9

4 Statistical analysis 10

1 Summary of the protocol

Title	Evolution of pain at three months by oral resveratrol in primary knee osteoarthritis: a multicenter, double-blind, randomized, placebo-controlled trial
Acronym	ARTHROL
Coordinating Investigator<br><br><br><br><br><br><br><br><br><br><br>Scientific Director	Prof. François RANNOU, M.D., Ph.D.<br>Service de Rééducation et de Réadaptation de l'Appareil Locomoteur et des Pathologies du Rachis<br>Hôpitaux Universitaires Paris Centre - Groupe Hospitalier Cochin<br>Assistance Publique – Hôpitaux de Paris<br>27, Rue du Faubourg Saint-Jacques, 75014 Paris, FRANCE<br>Tel +33 1 58 41 25 35<br>Fax +33 1 58 41 25 45<br>francois.rannou@aphp.fr<br><br>Prof. Christelle NGUYEN, M.D., Ph.D.<br>Service de Rééducation et de Réadaptation de l'Appareil Locomoteur et des Pathologies du Rachis<br>Hôpitaux Universitaires Paris Centre, Groupe Hospitalier Cochin<br>Assistance Publique – Hôpitaux de Paris<br>27, Rue du Faubourg Saint-Jacques, 75014 Paris, FRANCE<br>Tel +33 1 58 41 29 45<br>Fax +33 1 58 41 28 35<br>christelle.nguyen2@aphp.fr
Sponsor	Assistance Publique - Hôpitaux de Paris
Scientific justification	OA is the first cause of handicap in individuals over 40 years-old in France. OA physiopathology is driven by local joint inflammation responsible for pain and joint destruction. Experimental studies have shown that resveratrol could modulate pain and inflammation. We hypothesize that resveratrol, in a new formulation developed by the coordinating investigator and his colleagues (INSERM U1124), improving its bioavailability, will decrease pain in patients presenting with primary knee OA.

Primary objective and assessment criterion	The primary objective of the study is to assess the efficacy on mean knee pain in the previous 48 hrs of oral resveratrol compared to placebo in patients with knee OA at 3 months.<br><br>The assessment criterion is the mean change from baseline in mean knee pain in the previous 48 hours on a self-administered 11-point pain numeric rating scale (NRS, 0 no pain - 100 maximal pain) at 3 months
Secondary objectives and assessment criteria	The secondary objectives of the study are to assess the efficacy of oral resveratrol compared to placebo in patients with knee OA mean knee pain in the previous 48 hrs at 6 months, and function, patient's global assessment, response to treatment and medication (intra-articular injections of corticosteroids or hyaluronic acid, analgesics and non-steroidal anti-inflammatory drugs [NSAIDs]) sparing effect at 3 and 6 months. Assessment criteria are:<br><br>• the mean change from baseline in mean knee pain in the previous 48 hrs on a self-administered 11-point pain NRS at 6 months<br>• the mean change from baseline in the function subscore of the self-administered Western Ontario and McMaster Universities Arthritis Index (WOMAC) questionnaire at 3 and 6 monthsthe<br>• the mean change from baseline in patient's global assessment at 3 and 6 months on a self-administered 11-point global assessment NRS (0 worst possible - 100 best possible)<br>• the percentage of Osteoarthritis Research Society International (OARSI) - Outcome Measures in Rheumatology (OMERACT) responders at 3 and 6 months<br>• self-reported number of intra-articular injections of corticosteroids or hyaluronic acid since last contact at 3 and 6 months<br>Self-reported consumption of analgesics (non-opioid, weak and strong opioids) since last contact using a self-administered 4-class scale (never; several times a month; several times a week; daily) at 3 and 6 months<br><br>• self-reported consumption of NSAIDs since last contact using a self-administered 4-class scale at 3 and 6 months
Experimental design	Multicenter, double-blind, randomized, placebo-controlled trial
Population involved	Patients presenting with symptomatic primary knee OA, fulfilling 1986 American College of Rheumatology (ACR) classification criteria
• Inclusion criteria	• Age ≥ 40 years-old<br>• Knee OA fulfilling 1986 ACR criteria<br>• Pain on an 11-point NRS ≥ 40/100<br>• Symptom duration ≥ 1 month<br>• 4 > Kellgren and Lawrence X-Ray score ≥ 1<br>• Written consent obtained<br>• Health insurance cover<br>• Patients excluded for temporary reasons can be rescreened.

• Non-inclusion criteria	• History of symptomatic crystal or inflammatory arthritis<br>• Knee surgery ≤ 1 year<br>• Knee trauma ≤ 2 months<br>• Knee intra-articular injections ≤ 2 months<br>• Current use of intramuscular, intravenous or oral corticosteroids<br>• Current use of anticoagulants<br>• Uncontrolled diseases that may require intramuscular, intravenous or oral corticosteroids<br>• Neurologic disorders involving the lower limbs<br>• Inability to speak, write or read French language<br>• Participation to another biomedical research<br>• Contraindication to resveratrol or hypersensitivity to any of its constituents
Investigational product	Resveratrol is a dietary supplement, not a drug. It will be administered orally 2 caplets twice a day for one week then one capsule twice a day for a total duration of 6 months. Resveratrol will be supplied by the industrial partner. The caplets of resveratrol have already been distributed on the French market for several years and the caplets used in this study will be exactly the same as those already available on the French market.
Control group	Placebo of resveratrol will be supplied by the industrial partner. It will present with similar conditioning and taste, and will be administered orally 2 caplets twice a day for one week then one capsule twice a day for a total duration of 6 months. The placebo use in this study will be exactly the same as the one used in a previous PHRC already accepted in neurology (Assistance Publique-Hôpitaux de Paris).
Other procedures added by the research	Not applicable
Risks added by the research	Risk A
Practical procedure	• Recruitment of eligible patients by advertisement and among in- and outpatients of the investigating centers<br>• Day 0: inclusion visit, informed consent, inclusion and randomization<br>• Month 3: follow-up visit<br>• Month 6: follow-up visit and end of the research
Number of subjects chosen	164
Number of centres	3 tertiary care centers located in France<br>• Cochin Hospital, Paris<br>• Saint-Antoine Hospital, Paris<br>• Clermont-Ferrand Hospital
Research period	• Duration of participation for each patient: 6 months<br>• Duration of recruitment: 48 months<br>• Total duration: 54 months

Number of inclusions expected per centre and per month	1.1 patients per centre and per month
Statistical analysis	Analysis will be performed according to the intention-to-treat principle by which each participant will be analyzed in his randomization arm, following a prespecified statistical analytic plan
Funding source	Assistance Publique-Hôpitaux de Paris - Programme Hospitalier de Recherche Clinique en 2015
Data Safety Monitoring Board anticipated	No

2 Analysis population

2.1 Flow diagram

At the final analysis of trial, a flow chart will be constructed according to the CONSORT 2010 reporting guidelines. It will describe:

- The number of eligible patients, randomized patients and the number of patients who have actually followed the study;
- The intervention arm allocated per randomization;
- Early cessation of the intervention and their causes and drop-outs;
- The number of patients excluded from the analysis.

The number of randomized but ineligible patients, if any, will also be reported, as well as the reason for ineligibility.

2.2 Definition of the analysis population

For interim monitoring, the analysis will be carried out according to the intention to treat (ITT) principle, i.e. each randomised participant will be analysed in the group assigned to him/her by randomisation, regardless of the actual treatment received or other protocol deviations. In particular patients randomised while not meeting eligibility criteria will be kept in the analysis.

2.3 Sample size

The sample size was estimated at 164 patients. We have predicted a difference in mean change from baseline of 15 points on the pain NRS between resveratrol and placebo groups, with a SD of 27 points, and a power of 90%, corresponding to 69 patients in each arm. Considering a 15% lost to follow-up, we needed to enrol an estimated 82 patients for each arm. Fifteen points on pain NRS is considered the minimal clinically perceived difference in pain for patients with knee OA.

3 Outcomes

The primary efficacy outcome is the mean change from baseline in mean knee pain in the previous 48hours on a self-administered 11-point pain NRS (0, no pain, to 100, maximal pain) at 3 months.

The secondary efficacy outcomes are:

- the mean change in mean knee pain on a pain NRS at 6 months,
- the mean change in the function subscore of the self-administered Western Ontario and McMaster Universities Arthritis Index (WOMAC) questionnaire at 3 and 6 months (the French version of the questionnaire)
- the mean change in patient global assessment at 3 and 6 months on a self-administered 11-point global assessment NRS (0, worst possible, to 100, best possible),
- the proportion of responders according to the OARSI–OMERACT at 3 and 6 months. Response to treatment will be defined as an improvement in pain (assessed by an 11-point pain NRS) or in function (assessed by the WOMAC function subscore) ≥ 50% and absolute change ≥ 20, or improvement in at least 2 of the 3 following: 1/ pain ≥ 20% and absolute change ≥ 10, 2/ function ≥ 20% and absolute change ≥ 10, 3/ patient's global assessment (assessed by an 11-point global assessment NRS) ≥ 20% and absolute change ≥ 10.
- the self-reported number of intra-articular injections of corticosteroids or hyaluronic acid since the last contact at 3 and 6 months.
- the self-reported consumption of analgesics (non-opioid, weak and strong opioids) since the last contact on a self-administered four-class scale (never, several times a month, several times a week, daily) at 3 and 6 months.
- the self-reported consumption of NSAIDs since the last contact on a self-administered four-class scale (never, several times a month, several times a week, daily) at 3 and 6 months.

4 Statistical analysis

The profile of selected patients and their effective follow-up through the course of the trial will be carried out in accordance with the CONSORT statement, according to a patient flow diagram. Subjects withdrawing from the study early and the reason for this will also undergo a descriptive analysis by group and for the total population. The patient follow-up parameters will be analysed for each treatment group and for the total population:

- Total follow-up duration;
- Treatment duration;
- Number of visits;
- Treatment compliance.

For each group, and at each of the evaluation dates, the qualitative endpoints will be described by their sample size, percentage and data missing by response method, and the quantitative endpoints will be described by their sample size, mean, standard deviation, median and interquartile range (25th percentile - 75th percentile), as well as the minimum and maximum.
The analysis population will involve all patients randomised into their randomisation group except:

2) Patients withdrawing their informed consent and permission to use their data;
3) Patients not having given their consent.

A randomised patient who has not undergone the intervention or has partially undergone it will still be analysed. A description by group of these protocol deviations will be provided.

The primary endpoint is the change in the pain NRS score between the baseline and 3 months. The variable to be studied will therefore be the difference in Pain NRS score between randomisation (D0) and the Month 3 visit: Δ = value at M3 – value at D0. The other differences will also be calculated (between the Month 6 visit and the enrolment visit). Comparison of the differences in Δ between the groups will be studied with a CLDA (Constrained Longitudinal Data Analysis) linear model, taking into account the correlation of repeated measurements in the same subject (random patient effect with an unstructured variance-covariance matrix) under the hypothesis of randomly missing data. In this model, the baseline values are included in the response vector with the only condition that the average of this baseline must be the same for each group, which is the case in our studies as the subjects

are randomly distributed. There is therefore no reason, in theory, to believe that one group would have a higher baseline than another. The CLDA technique is consistent with the intention-to-treat principle provided that all patients have at least one initial value for the endpoint or a post-baseline value, which allows all eligible randomised patients to be included. The results will be expressed in the form of the difference between the average changes from baseline in each of the groups at 3 and 6 months with a 95% confidence interval and p-value of the associated test. Random effects at the patient level and fixed effects at the center level were added to these models. The following criteria will be analysed in the same way as the quantitative primary endpoint: WOMAC function subscore at 3 and 6 months, patient global assessment at 3 and 6 months and the self-reported number of intra-articular injections of corticosteroids or hyaluronic acid at 3 and 6 months (for this variable, a negative binomial regression model will be considered if the distribution of the variable requires it. The results will be presented in the form of mean ratio (with a 95% confidence interval) in this case).

For the proportion of responders according to the OARSI–OMERACT at 3 and 6 months, a Poisson model under GEE framework (adjustment for center will be applied) with log link allows to estimate the percentage differences (with a 95% confidence interval and p-value for the associated test) at 3 and 12 months (as well as the relative risk).

The same model will be used for the self-reported consumption of analgesics (non-opioid, weak and strong opioids) at 3 and 6 months and the self-reported consumption of NSAID at 3 and 6 months after dichotomization (never vs several times a month or several times a week or daily).

The frequency of adverse events (serious, non-serious or both) will be described in each of the 2 groups (no statistical tests are planned).

All the tests will be bilateral and at the 5% threshold. The confidence intervals will be calculated at 95%. The CLDA model (MIXED procedure) will be carried out using SAS 9.4 software (SAS Institute). The other analyses will be carried out using R 3.2.2 software (R Foundation for Statistical Computing). The glm function will be used for the Poisson model and to estimate the confidence interval of the difference between the proportions. The glm.nb 27 and coef.test functions will be used for negative binomial regression (with robust estimation of variance). Other software or other functions may be used if necessary. Blinded statisticians will perform the statistical analyses at an independent centre (Centre d'Épidémiologie Clinique, Paris Descartes, Hôpital Hôtel-Dieu).
