# Supplementary material for: Oral resveratrol in adults with knee osteoarthritis: A randomized placebo-controlled trial (ARTHROL)
Source: PLoS Med. 2024 Aug 13;21(8):e1004440. doi: 10.1371/journal.pmed.1004440 (PMC11321588; doi:10.1371/journal.pmed.1004440)
Supplement: Attachment — Submitted filename: (01) Response to reviewers.docx [file pmed.1004440.s009.docx]

|  | **Comments** | **Response to reviewer #1** | **Tracked**  **changes** |
| --- | --- | --- | --- |
| **Reviewer #1** | *The authors report the results of a placebo-controlled randomized clinical trial of oral resveratrol in adults with osteoarthritis (OA) of the knee. The study was designed appropriately and the manuscript is clearly written. I have no doubt that the results are valid. My only request is that the authors delete P-values in Table 2 for the secondary outcomes. It is not appropriate to perform hypothesis tests on secondary outcomes if the primary outcome does not achieve significance at the 0.05 level.* | As requested, we now deleted all p-values in Table 2 for the secondary outcomes. | **Table 2** |

|  | **Comments** | **Response to reviewer #2** | **Tracked**  **changes** |
| --- | --- | --- | --- |
| **Reviewer #2** | *This manuscript presents the results of a randomised placebo-controlled trial assessing the effect of oral resveratrol in patients with knee osteoarthritis. My comments relate to the statistical aspects of this manuscript. For the most part, the statistical analysis presented in the manuscript reflects that planned in the SAP, however I have a few questions and comments about the analysis and the presentation of results.*  *What is meant by "first cause of disability"? Is this the most common cause of disability in this group?* | Thank you. We now rephrased the sentence, as suggested. | **Line 90** |
|  | *The "French paradox" is mentioned: the observation of low rates of death from coronary heart disease despite high intakes of cholesterol and saturated fat.* *Such a paradox, like the "obesity paradox", is likely to be, at least partially, a result of collider stratification bias. See, for example PMID: 37286200; PMID: 24270963. If this paradox is mentioned, that there is a likely selection bias explanation of this paradox must also be mentioned.* | Thank you. We now mentioned the selection bias explanation and the two references PMID: 37286200; PMID: 24270963, as suggested. | **Lines 113 to 114, references 28 and 29** |
|  | *When the Hussain et al paper is mentioned in the Introduction, the changes within each randomised group are mentioned. It would be more appropriate to describe the change between groups that Hussain et al found when describing this paper, since change within groups is not what randomised trials are designed to detect.* | Thank you. We now described the change between groups reported in Hussain et al. paper. | **Lines 121 to 123** |
|  | *The conditional longitudinal data analysis model applied by the authors has been the subject of criticism in the statistical literature - as pointed out in PMID: 20527014. A key criticism holds for the analysis presented here if restricted maximum likelihood (REML) has not been applied: if REML was applied, please state this. If not, please revise your analysis with REML applied.* | Thank you for this comment. We now clarified that we used REML method. cLDA model is now recognized as a method of choice when analyzing longitudinal data in RCTs (Coffman CJ, Edelman D, Woolson RF. To condition or not condition? Analysing 'change' in longitudinal randomised controlled trials. BMJ Open. 2016 Dec 30;6(12):e013096. doi: 10.1136/bmjopen-2016-013096. PMID: 28039292; PMCID: PMC5223669.) | **Line 247** |
|  | *When differences in mean change from baseline were calculated given the results of the cLDA model, were these derived as linear combinations of the estimated parameters from the cLDA model? Please provide a bit more detail in the analysis section.* | We added details on the estimated parameters from the clDA model. | **Lines 258 to 268** |
|  | *In the SAP it was mentioned that GEE-based analyses were applied for binary outcomes, but this is not mentioned in the main paper. Please clarify if the planned GEE approach was used and specify what working correlation matrix was assumed. If the planned GEE approach cannot be applied please explain why.* | Thank you for this comment. We fully agree that this point needs clarification. The planned GEE approach was not used because we failed to estimate marginal variance of difference in proportions between groups when “center” is considered as fixed effect. Moreover, our aim was to use the same model to estimate both the difference in proportions and the relative risk. That's why we've changed our method of analysis and we have used a Poisson model with log link under regression standardization framework to produce correct marginal estimates and variances. | **Lines 277 to 278** |
|  | *Instead of stating that there was no difference between the resveratrol and placebo groups, it is more appropriate to say that* *the estimated differences between groups were small* *with wide confidence intervals. A difference between groups was observed - it was just not clinically meaningful was not estimated with a high degree of precision.* | Thank you. We now revised our interpretation throughout the manuscript, as suggested. | **Lines 303 to 304, 319 to 320 and 417 to 418** |
|  | *Were patients expected to take all supplied caplets?* | No, they were not. Overall, 392 caplets were necessary for the whole duration of the study, but more caplets were supplied (i.e, 420). We now mentioned this in the methods. | **Lines 196 to 197** |
|  | *Please provide a comparison of the baseline characteristics of participants who provided the primary outcome compared to those who did not. The implications of the drop out of patients on the results should be included in the Discussion. Why was only a complete case analysis pre-specified?* | We agree that it is important to provide characteristics of patients with available primary outcome (n=135) vs patients with missing primary outcome (n=7). We added this description in Appendix 4. As suggested, we added implications of drop out of patients in the discussion.  We used a cLDA model as a main analysis. This analysis differs from the analysis of complete cases because all patients with at least one assessment of the outcome (e.g. one baseline or one post-baseline assessment) are included in the analysis. In our study, all patients have at least one measure of the primary outcome and all patients contributed to estimating the treatment effect. Furthermore, this mixed model makes the hypothesis that missing data can be characterized by observed quantities. In fact, for many longitudinal RCTs is likely that the probability of missing data is related to some observable characteristics (eg, treatment arm, available outcome assessments, time of assessment). Under this reasonable assumption, unbiased estimates can be achieved by cLDA models without the need for imputation methods. | **Lines 410 to 416 and Appendix 4** |
|  | *In the flow chart, it seems that patients were assessed for eligibility on certain characteristics after been randomly allocated to groups. Is this correct? What happened with the further participation of these patients?* | This is partially correct. When these individuals were formally assessed for inclusion criteria, they mentioned a pain intensity score of at least 40 on a self-administered 11-point pain numeric rating scale in 10-point increments, so that they were eligible to enter the study and could be randomized. However, when they completed their self-administered questionnaires, on the same day as randomization, they actually indicated a pain intensity score lower than 40. The flow chart aimed at reflecting these inconsistencies in participants’ responses.  These patients were included, followed-up for 6 months and analyzed. We now clarified this point in the figure footnote. | **Figure 1 footnote** |

|  | **Comments** | **Response to reviewer #3** | **Tracked**  **changes** |
| --- | --- | --- | --- |
| **Reviewer #3** | *In this study, the authors aim to determine whether resveratrol supplementation as an add-on therapy to usual care, could reduce knee pain at 3 months in individuals with painful knee osteoarthritis.*  *The study holds clear scientific interest as numerous in vitro and animal studies demonstrate a significant effect of this molecule on inflammation. As the authors note, however, there are no clinical studies that have confirmed these findings. Importantly, the authors have modified the galenic form of the molecule, overcoming the low digestive absorption of trans-resveratrol by using it as a dry powder.*  *The clinical trial design is appropriate and aligns perfectly with the study objectives. It was a double-blind, randomized, placebo-controlled, phase 3 trial conducted in 3 tertiary care centers.*  *Despite the study's limitations, such as the lengthy recruitment period, the lack of control over other treatments received by the patients or the inability to recruit the anticipated number of patients, the data from the study are interesting because these compounds need to be seriously studied in clinical setting. Indeed, there is very limited information available on this matter.* | We would like to thank reviewer #3 for his/her kind comments | **Not applicable** |
|  | *Minor point:*  *"but pain intensity reduction was minimal in the placebo group". Was it a real placebo or a meloxicam group?* | It was a real placebo not a meloxicam group | **Not applicable** |

|  | **Comments** | **Response to reviewer #4** | **Tracked**  **changes** |
| --- | --- | --- | --- |
| **Reviewer #4** | *My thanks for the invite to review this thoughtful, well conducted and reported randomised trial of resveratrol vs matched placebo for knee osteoarthritis.*  *Generally speaking, I find this paper to be of very high quality, and the thoroughness of the research team is apparent in the good study design, appropriate outcomes, and inclusion of additional documentation that help allow scrutiny and therefore boost transparency of this project - the teams' efforts are certainly appreciated. I have several comments on the paper, but would not hesitate to recommend this paper be published in PLoS Medicine following authors' response to the following (minor) queries/comments:*  *- This trial is preregistered, and the statistical analysis plan and protocol are included as appendices, which is commendable and something I would like to highlight as good practice.* | We would like to thank reviewer #3 for his kind comments | **Not applicable** |
|  | *- The choice of methodology (double blind, randomised, placebo controlled with permuted block randomisation) is a very strong methodology for asserting causal inference, which is also notable.* | Thank you | **Not applicable** |
|  | *- The 'Evidence in context' section whereby the team undertake a 'mini' systematic review to find similar trials is in line with best practice, very good, and in my view, not seen often enough in trials.* | Thank you | **Not applicable** |
|  | *- It appears that the 'conversion rate',* *the number of patients screened vs. those randomised appears quite low, despite the inclusion/exclusion criteria being, in my view, relatively relaxed (K-L grades 1-3 includes a wide range of disease severity).* | We agree. We now mentioned this as a limitation of our study. | **Lines 409 to 410** |
|  | *- Why did the team opt to include this range - K-L grade 1 is often considered borderline OA, and grade 3 are relatively severe (as the authors note), which perhaps might be considered too severe for a food supplement-type intervention which is typically used as an add-on therapy, implying the effect size might normally be considered quite modest (notwithstanding the teams' assumed effect size of 0.55 used in the sample size calculation - something the team themselves acknowledge)* | Thank you for this comment. We chose to include Kellgren and Lawrence grades 1, 2 or 3 on X-rays because: 1/ we wanted to reflect the use of dietary supplements that are offered indepently of structural damages, 2/ our therapeutic target was pain and not structure, and 3/ there is no consistent correlation between Kellgren and Lawrence grades grades and pain intensity. We now clarified this design choice in the methods. We also amended Table 1, to simplify the description of Kellgren and Lawrence grades in our population. | **Lines 160 to 164 and Table 1** |
|  | *- I feel there is a* *question over whether the dose formulation might be considered large enough to produce a therapeutic effect - the evidence cited suggested that the dose used did remain in the bloodstream of patients, however there is no comment on efficacy. Some other studies cited used doses over 10 times that used in this trial, and in line 183 - the term 'biologically efficient' does not make clear whether this dose was sufficient to produce the anti-inflammatory effect assumed to be the active effect of this drug.* | We agree. In the pharmacokinetics study of the resveratrol formulation used in ARTHROL trial (ref 31), the dose formulation was sufficient to increase anti-inflammatory markers production in three different organs at the level of mRNA expression (i.e. liver IL-10, colon HO-1 and hypothalamus PAI-1) in the model of HFD-fed C57Bl/6J wild-type mice. However, whether these results may be translated in human inflammatory conditions has not been assessed yet. We now enhanced this point in the discussion. | **Lines 356 to 365** |
|  | *- The constrained longitudinal data analysis (cLDA) is an appropriate choice of analysis methodology which makes use of the 2 post-baseline observations simultaneously, making the best use of the available data. However, using the 6-month data in this way 'weights' the 3-month estimate by the 6-month, as this estimate is now taken from part of a linear trajectory between the two time points. As the primary outcome is principally concerned only with the 3-month estimate, as a sensitivity analysis, to avoid making assumptions about this linear trajectory over time, an alternative analysis method which avoids any assumptions about longitudinal trajectories would be to use the baseline-as-a-covariate (ANCOVA) method, whereby the 3 month observation would be the outcome variable in a linear regression model, and the baseline observation would be a predictor (covariate). I appreciate, however, that the lack of power in this study would not likely make an appreciable difference to the conclusions of the analysis in this case.* | Thank you for this comment. As a sensitivity analysis, we added both results from cLDA when considering only baseline and 3 months data (line 2 of the table) and results from classical ANCOVA (line 3 of the table). Estimation of treatment effect were very closed to the main results (line 1) | **Appendix 5** |
|  | *- Missing data methods. The primary cLDA model used makes no special consideration for the missing observations, and while the analysis included all participants, there was no special treatment for the observations lost to follow-up, implying that the analysis was done under a MCAR (complete case analysis) assumption. Perhaps a sensitivity analysis that considers a different missingness assumptions might be useful to present alongside this (e.g. using multiple imputation to estimate plausible missing values), to assess robustness of the findings.* | The cLDA model makes the hypothesis that missing data can be characterized by observed quantities (Missing At Random (MAR) assumption). In fact, for many longitudinal RCTs is likely that the probability of missing data is related to some observable characteristics (eg, treatment arm, available outcome assessments, time of assessment). Under this reasonable assumption, unbiased estimates can be achieved by cLDA models without the need for imputation methods.  As suggested, we also performed a sensitivity analysis by imputing missing data of primary outcome in the context of ANCOVA model. We have considered Worst-case scenario and Best-case scenario. Worst-case scenario assumes missing NRS knee pain values at 3 months in Resveratrol group had the worst possible value (=100) and those in control group had the best possible value (=0). Best-case scenario assumes missing NRS knee pain values at 3 months in Resveratrol group had the best possible value (=0) and those in control group had the worst possible value (=100). The results confirm (line 4 and 5 of the table) that there is no evidence of difference between arms for primary outcome. | **Appendix 5** |
|  | *- Lines 191-197 explain that* *concomitant analgesia was unlimited and prescribed as necessary by the participating physicians: I guess* *one alternative explanation for the negligible between-groups effect observed is that many participants are 'masking' the effect of resveratrol with their concomitant analgesics - I note that in table 2 over half the sample were taking at least one analgesic at 6 months.* | Thank you. We agree this may be another plausible explanation. We now mentioned it in our discussion. | **Lines 383 to 387** |
|  | *- As the team note, the anticipated effect size of 0.55 is substantially greater than expected by many treatment effects produced by treatments for osteoarthritis, and certainly larger than many other food supplements. This, combined with the unfortunate truncation of the trial due to the COVID-19 pandemic in essence, leaves the trial 'doubly underpowered' to detect effects of the magnitude observed, which is unfortunate.* | Thank you. We extensively discussed this as a limitation of our trial. | **Lines 401 to 409** |
|  | *- Again, while there are several comments here, they are intended to be suggestions to better strengthen the paper - overall, I think this is really excellent work, and the additional detail provided by the team have allowed me to get into the level of detail required to properly scrutinise this trial, which again is very good, and the team have clearly thought carefully about the design of the trial and the implications of the decisions on the interpretation of the results.* | Again, we would like to thank reviewer #3 for his kind comments | **Not applicable** |
