# Supplementary material for: Oral resveratrol in adults with knee osteoarthritis: A randomized placebo-controlled trial (ARTHROL)
Source: PLoS Med. 2024 Aug 13;21(8):e1004440. doi: 10.1371/journal.pmed.1004440 (PMC11321588; doi:10.1371/journal.pmed.1004440)
Supplement: Attachment — Submitted filename: (01) Response to reviewers.docx [file pmed.1004440.s010.docx]

|  | **Comments** | **Response to editors** | **Tracked**  **changes** |
| --- | --- | --- | --- |
| **Editors** | *The Editors agree with Reviewer #4 on the reinstatement of the p-values for the secondary outcomes. Please revise accordingly.* | We now re-include p-values from Table 2 | **Table 2** |
|  | *Please cite the reference numbers in square brackets. Citations should be preceding punctuation.* | Amended | **References** |
|  | *Please add this statement to the manuscript's Competing Interests: "CN is an Academic Editor on PLOS Medicine's editorial board."* | Amended | **Competing interests** |
|  | *The Data Availability Statement (DAS) requires revision. Please note that a study author cannot be the contact person for the data.* | We now revised the DAS accordingly | **DAS** |
|  | *l.59: Please define ‘SD’ at first use.* | We now defined ‘SD’ at first use | **Line 59** |
|  | *l.61: Please define ‘CI’ at first use.* | We now defined ‘CI’ at first use | **Line 62** |
|  | *In the last sentence of the Abstract Methods and Findings section, please describe the main limitation(s) of the study's methodology.* | We now described the main limitations of the study’s methodology | **Line 65 and 66** |
|  | *Please change the sub-heading ‘Interpretation’ to ‘Conclusions’.* | Amended | **Line 67** |
|  | *Abstract Conclusions: Please change to "**In this study, we observed that compared with placebo, oral resveratrol did not reduce knee pain in people with painful knee osteoarthritis". You may add a short sentence about the implications for future clinical research.* | Amended | **Line 67 to 69** |
|  | *ll.69-87: Please remove the section ‘Research in context’ and provide the Author Summary.* | We now provided the author summary | **Lines 72 to 91** |
|  | *l.88: Please provide a heading for the Introduction section.* | Amended | **Line 92** |
|  | *l.154: We suggest introducing the abbrevation ‘NRS’ here (i.e., “…11-point pain numeric rating scale (NRS)..”).* | We now introduced ‘NRS’ | **Lines 159 and 160** |
|  | *2) ll.213-217: Please include the two links provided here as references and remove the links from the main text.* | Amended | **Citations and references 40 and 41** |
|  | *3) l.232: Please define ‘WHO’ at first use.* | We now defined ‘WHO’ at first use | **Line 238** |
|  | *4) l.237: Please define ‘SD’ at first use.* | We now defined ‘SD’ at first use | **Line 243** |
|  | *5) ll.257-258: Please write ‘cLDA’ and ‘ANCOVA’ in full (“As a sensitivity analysis, we also analyzed primary outcome with cLDA (**constrained longitudinal data analysis) model when considering only baseline and 3 months data and with classical ANCOVA (**analysis of covariance).”).* | We now wrote ‘cLDA’ and ‘ANCOVA’ in full | **Lines 263 to 265** |
|  | *6) l.272: Please write ‘GEE’ in full.* | We now wrote ‘GEE’ in full | **Line 279** |
|  | *7) ll.281-283: Please remove the funding statement from the main text. The information should only be included in the metadata of the online submission form.* | Amended | **Lines 290 to 292** |
|  | *1) l.353: Please define ‘HFD’.* | We now defined ‘HFD’ | **Line 362** |
|  | *2) ll.354-355: Please write 'IL-10' and 'HO-1' and 'PAI-1' in full or add the definition in parentheses after the abbreviation.* | We now wrote ‘IL-10’, ‘HO-1’ and ‘PAI-1’ in full | **Lines 364 and 365** |
|  | *3) l.398, please change to: “…COVID-19…”* | Amended | **Line 409** |
|  | *PLOS uses the numbered citation (citation-sequence) method and first six authors, et al. Please revise accordingly.* | We now revised the citations accordingly | **Citations and references** |
|  | *Figure 2: Please consider avoiding the use of red and green in order to make your figure more accessible to those with color blindness. Also, please write ‘NRS’ in full.* | We now revised figure 2 accordingly | **Figure 2** |
|  | *1) Thank you for providing the CONSORT checklist. Please replace the page numbers with paragraph numbers per section (e.g. "Methods, paragraph 1"), since the page numbers of the final published paper may be different from the page numbers in the current manuscript.* | Amended | **Appendix 1** |
|  | *2) Appendix 5: Please define ‘ANCOVA’* | We now defined ‘ANCOVA’ | **Appendix 5 footnote** |

|  | **Comments** | **Response to reviewer #2** | **Tracked**  **changes** |
| --- | --- | --- | --- |
| **Reviewer #2** | *Reviewer #2: I thank the authors for their responses to my comments on the previous version of this manuscript. I have only two follow-up comments.*  *1. I don't understand what is meant by "a failure to marginalize variance of differences in proportions" when describing why the pre-specified GEE-based approach was not used. Was the issue that random effects for only 3 centers were included? Please provide a clarification of this.* | We agree that this point needs clarification. We've gone back over the entire history and can better explain why we preferred not to use the GEE method. In fact, we should never have planned a GEE method when we only had 3 centers in the study. In these circumstances, it’s recommended to consider centers as fixed effects (Kahan: Accounting for centre-effects in multicentre trials with a binary outcome – when, why, and how?. BMC Medical Research Methodology 2014 14:20.). Following this choice, we encountered some difficulties in obtaining a reliable estimate of the variance of the marginal difference between proportion from the conditional model with centers as fixed effects; it was solved thanks to Poisson model with log link under regression standardization framework. We have modified the manuscript explain the original reason for the change | **Lines 279 to 284 and reference 48** |
|  | *2. Page 19, line 414: I suggest changing "have low effect on the estimate…" to "would have a minimal impact on the estimate…". Further, I suggest changing "Moreover sensitivity analysis on impact of missing data confirmed that there is no evidence of difference between arms for primary outcome." To "Moreover,* *our worst case and best case sensitivity analyses indicate that our conclusions are robust to a wide range of assumptions regarding this missing data. "* | We now revised these sentences accordingly | **Lines 417 to 421** |

|  | **Comments** | **Response to reviewer #4** | **Tracked**  **changes** |
| --- | --- | --- | --- |
| **Reviewer #4** | *The only minor comment I have for this revised version would be as follows:*  *Reviewer #1 states "My only request is that the authors delete P-values in Table 2 for the secondary outcomes. It is not appropriate to perform hypothesis tests on secondary outcomes if the primary outcome does not achieve significance at the 0.05 level." - I'd strongly disagree with this assertion. Please could the authors *re-include* the p-values from table 2.* | We now re-include p-values from Table 2 | **Table 2** |
